# Supplementary material for: Phosphorylation of P-stalk proteins defines the ribosomal state for interaction with auxiliary protein factors
Source: EMBO Rep. 2024 Oct 28;25(12):5478–506. doi: 10.1038/s44319-024-00297-1 (PMC11624264; doi:10.1038/s44319-024-00297-1)
Supplement: Supplementary file 1 — Appendix [file 44319_2024_297_MOESM1_ESM.pdf]

Appendix for

Phosphorylation of P-stalk proteins defines the ribosomal state for interaction with auxiliary protein factors

†Kamil Filipek and †Sandra Blanchet, et al.

†Contributed equally

*Corresponding authors

Kyle W. Cunningham - kwc@jhu.edu
Rachel Green - ragreen@jhmi.edu
Marina V. Rodnina - rodnina@mpinat.mpg.de
Marek Tchórzewski - marek.tchorzewski@mail.umcs.pl

Table of Contents

Appendix Figures ------ #3 - 22
- Fig. S1 ------ #3
- Fig. S2 ------ #4
- Fig. S3 ------ #5
- Fig. S4 ------ #6
- Fig. S5 ------ #8-7
- Fig. S6 ------ #9
- Fig. S7 ------ #10
- Fig. S8 ------ #11-12
- Fig. S9 ------ #13
- Fig. S10 ------ #14
- Fig. S11 ------ #15
- Fig. S12 ------ #16
- Fig. S13 ------ #17-18
- Fig. S14 ------ #19-20
- Fig. S15 ------ #21
- Fig. S16 ------ #22

Appendix Table
- Table S1 ------ #23 - 26

Appendix Figure S1

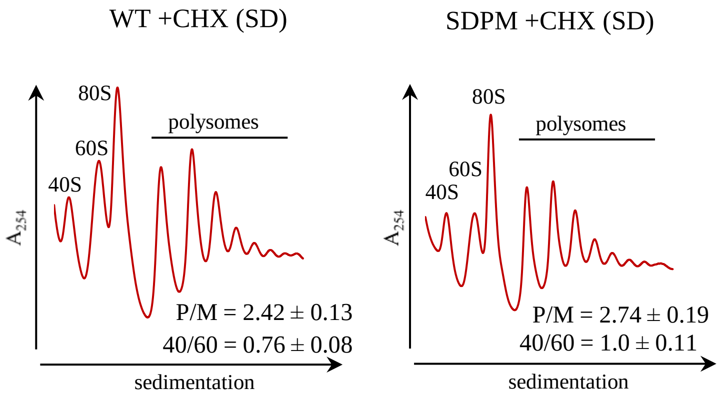

Appendix Figure S1. Polysome profile analyses from the WT and SDPM yeast strains grown on SD minimal medium suplemented with full set of amino acids.

The polysome-to-monosome (P/M) ratio was calculated for each profile by dividing the area of the first four polysomal peaks by the area of the peak for the 80S monosome. P/M value is presented as means ± SD (n = 3). The sedimentation vector of the ribosomal fractions is indicated by a horizontal arrow, and the optical density value at 254 nm is shown on the y-axis; the positions of individual ribosomal subunits are indicated.

Appendix Figure S2

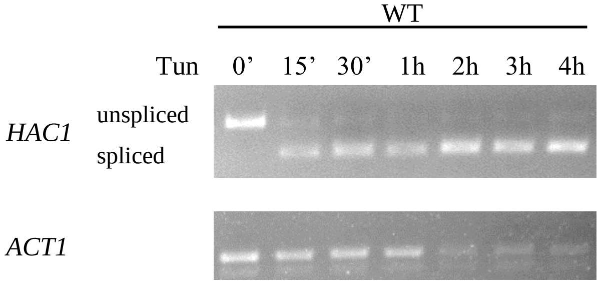

Appendix Figure S2. PCR analysis of HAC1 mRNA splicing . The time course of *HAC1* mRNA splicing was analyzed by PCR. The analysis was performed in the presence of tunicamycin (Tun) at a concentration of 2.5 μg/ml. 0' - control, without Tun, and the analysis was performer after 15, 30 minutes and 1, 2, 3 and 4 hours. *ACT1* - reference, analysis of the mRNA level for actin. Brifly, the cells were grown in YPD medium (1% yeast extract, 2% peptone, 2% dextrose) to an OD600 of 0.5 and treated with Tun to the final concentration, 2,5 μg/ml for 15 min, 30 min, 1h, 2h, 3h, 4h and then harvested by centrifugation at 3000 xg and subsequently flash-frozen in liquid nitrogen for storage. Total RNA was extracted from the cells following hot phenol method. The RNA samples obtained were subjected to DNase treatment with a TURBO DNA-free kit according to the manufacturer’s protocol (Ambion). 200 ng of RNA were used as a template for cDNA synthesis reaction which was set up in the volume of 20 μl using 200 U of SuperScript IV Reverse Transcriptase (Invitrogen). 10x Reverse Transcripton Random Primers (Applied Biosystems) were used in the reaction. cDNA was used as a template for PCR reaction and products were analyzed on 1% agarose gel. *HAC1* was amplified using the primers 5’-CACTCGTCGTCTGATACG-3’ and 5’-CATTCAATTCAAATGAATTCAAACCTG-3’, results in products 577 bp long for unspliced *HAC1* mRNA and 325 bp long for spliced mRNA. *ACT1* was amplified with primers 5’-CTGGTATGTTCTAGCGCTTG-3’ and 5’-GATACCTTGGTGTCTTGGTC-3’ results in products 431 bp long.

Appendix Figure. S3

Human - *Homo sapiens*
(uL10 - #P05388, P1 - #P05386, P2 - #P05387)

P1 - K^{1}VEAKKEE**S**EE**S**DDDMGFGLFD^{22}
P2 - K^{1}KDEKKEE**S**EE**S**DDDMGFGLFD^{22}
uL10 - A^{1}KVEAKEE**S**EE**S**DEDMGFGLFD^{22}

Yeast - *Saccharomyces cerevisiae*
(uL10 - #P05317, P1A - #P05318, P1B - #P10622, P2A - #P05319, P2B - #P02400)

P1A - A^{1}EKEEEEAKEE**S**DDDMGFGLFD^{22}
P2B - E^{1}EEKEEEAKEE**S**DDDMGFGLFD^{22}

P1B - E^{1}EEKEEEAAEE**S**DDDMGFGLFD^{22}
P2A - E^{1}EEKEEEAAEE**S**DDDMGFGLFD^{22}

uL10 - A^{1}EEAAAEEEEE**S**DDDMGFGLFD^{22}

Archaea - *Haloarcula marismortui*
(uL10 - #P15825, P1 - #P15772)

P1 - G^{1}GDDDDDEDDEASGEGLGELFG^{22}
uL10 - A^{1}EEADDDDDDDEDAGDALGAMF^{22}

Appendix Figure S3. The C-terminal polypeptide used for the MD simulation. The UniProt numbers for each P-stalk protein are given in parentheses. Phosphorylated serine residues are shown in bold; superscript numbers indicate the position of each residue within the peptide.

Appendix Figure. S4

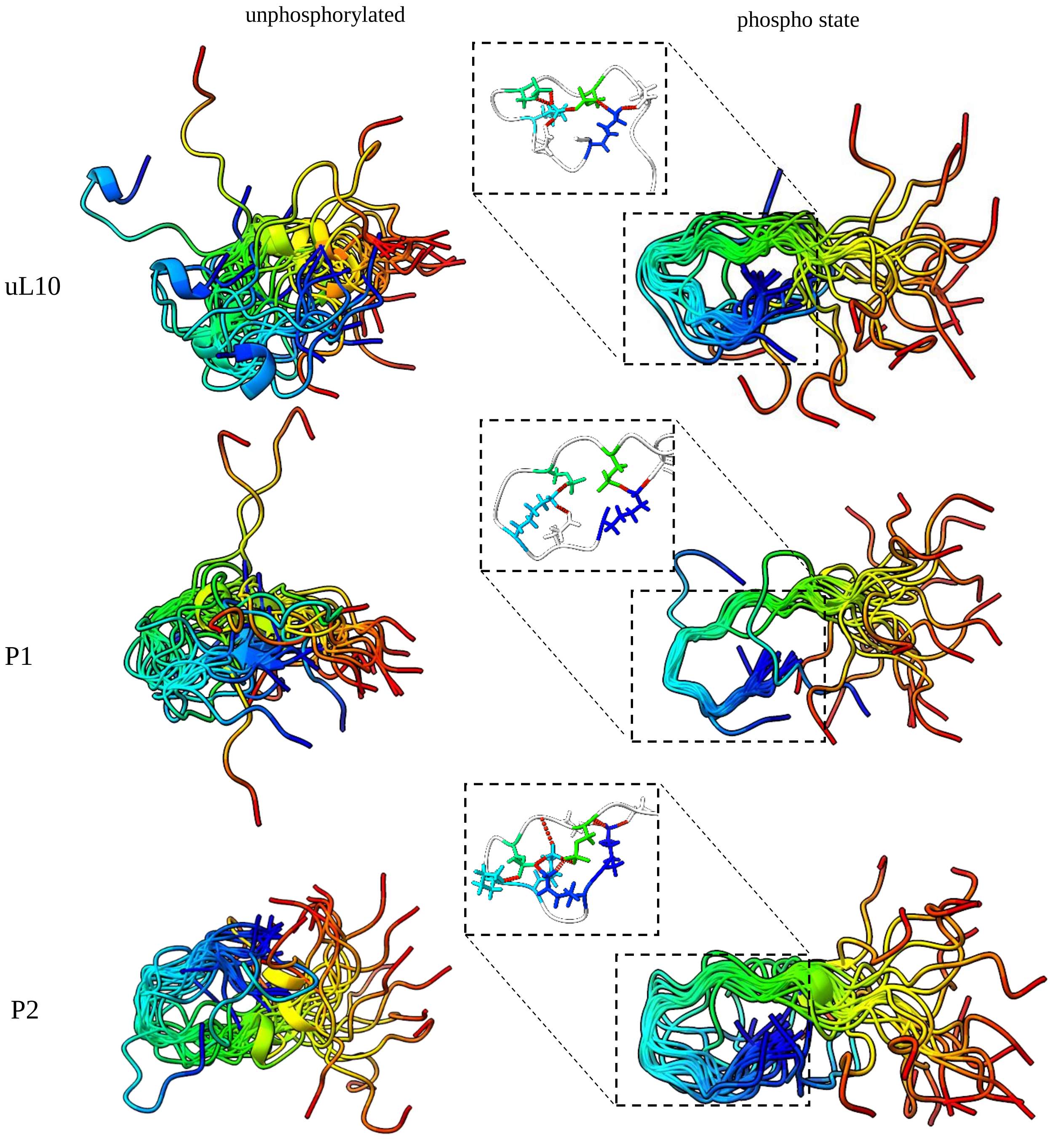

Figure S4. Molecular dynamics simulation of ribosomal P-stalk CTDs belonging to human uL10, P1 and P2 proteins as unphosphorylated and phosphorylated peptides. Medoids of the 20 highest scoring structures obtained for each type of CTD are shown. Insets - the peptide was shown with two phosphorylated serine residues (green) and lysine residues (light and dark blue); hydrogen bonds - red.

Appendix Figure. S5

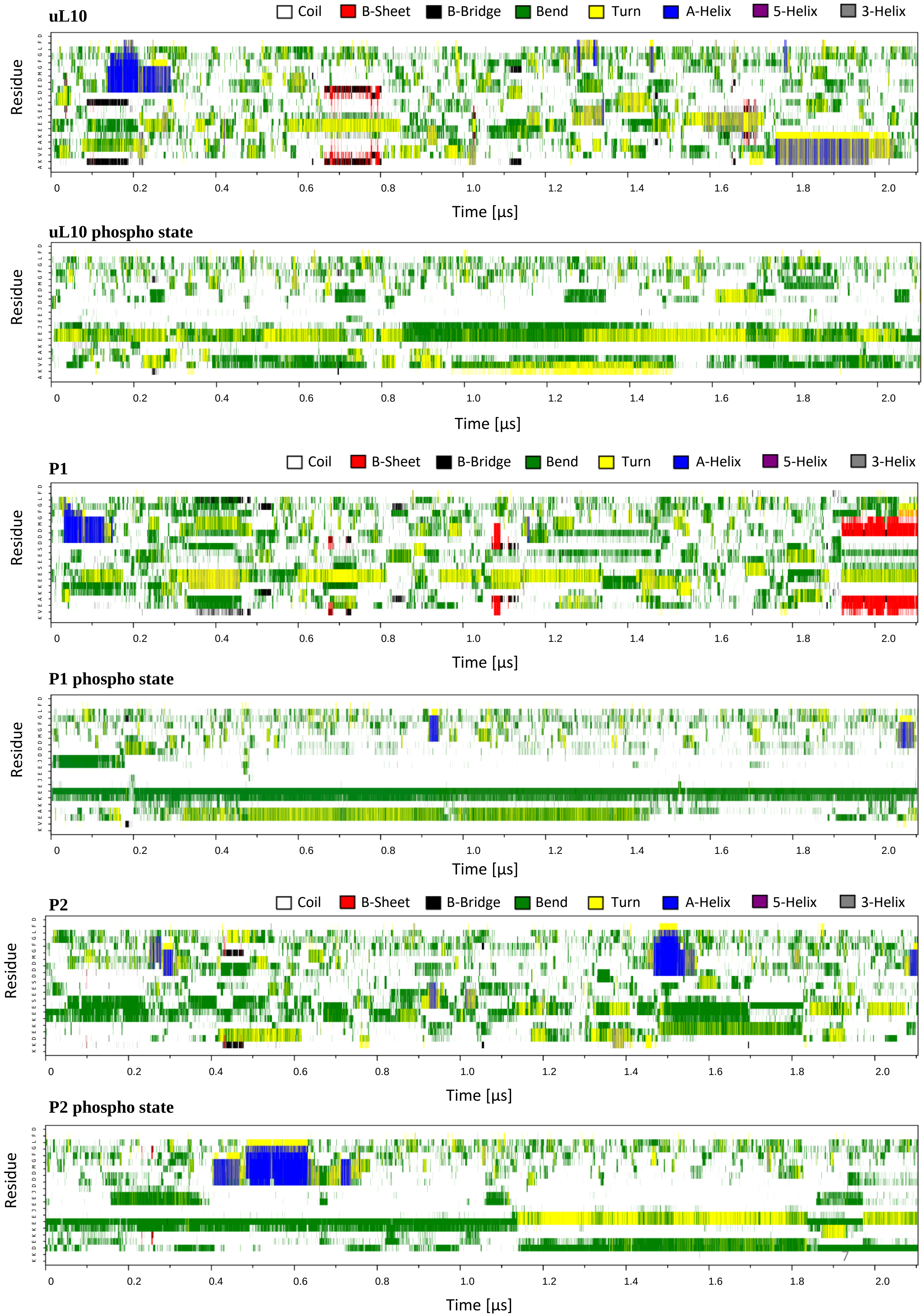

Appendix Figure S5. MD simulation of the linear polypeptide chain. The graph shows the occurrence of secondary structures versus time for the C-terminal peptides of human P proteins (uL10, P1, P2) folding in unphosphorylated and phospho residues. Secondary structure presented for individual peptide residues (vertical axis) vs. simulation time (horizontal axis) plot for unphosphorylated and phosphorylated human P-protein C-termini peptide folding. The structures were marked with individual color as marked in the legend. On the X - time of simulation, Y - position of individual amino acid residues, with phosphoserine marked as J.

Appendix Figure. S6

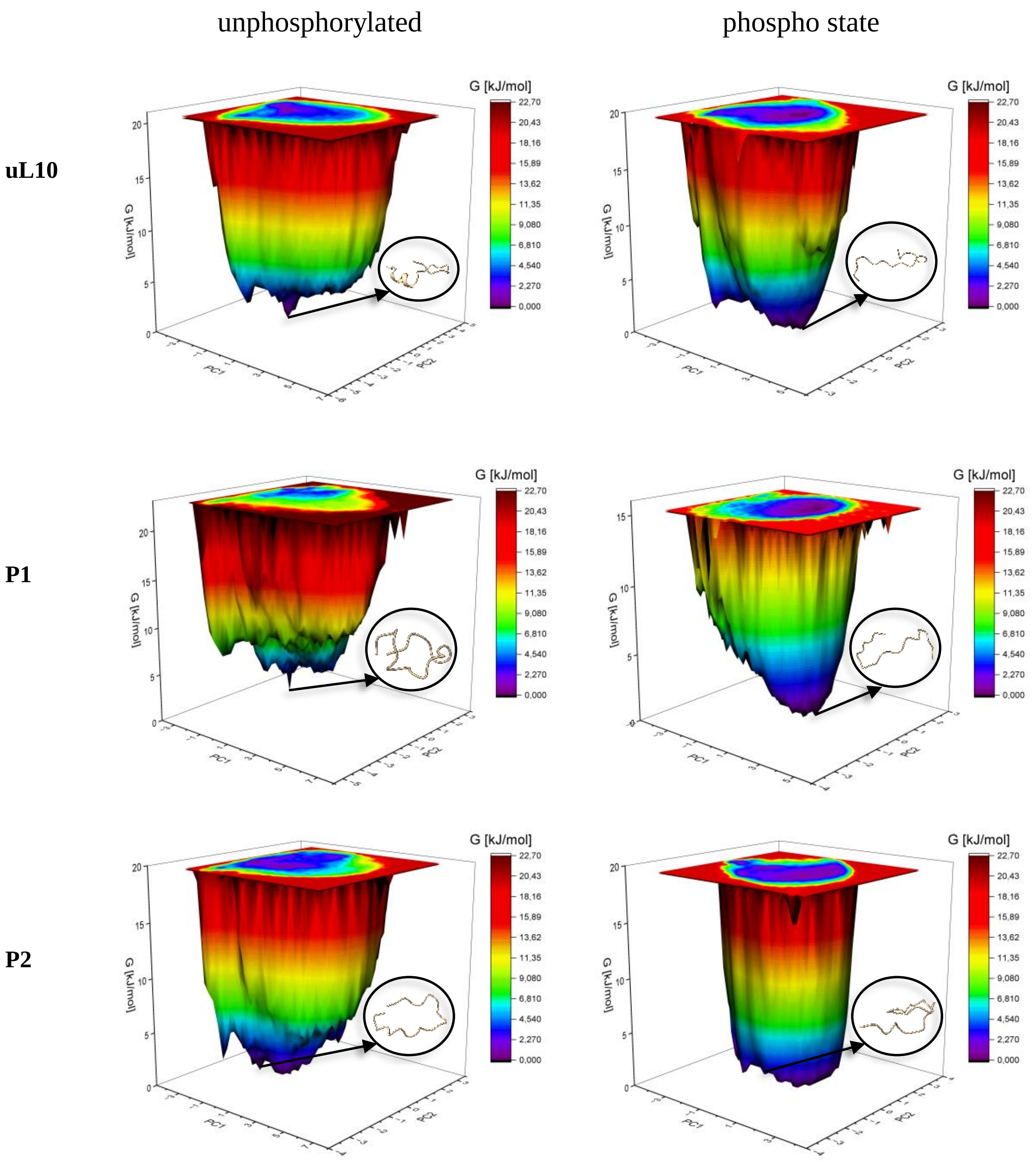

Appendix Figure S6. Free energy landscapes mapping of possible states of P-proteins (ul10, P1, P2) C-termini peptides folding in wild type (left side) and phospho state (right side) from *H.sapiens;* instet – representative lowest energy structures sampled during MD simulation.

Appendix Figure. S7

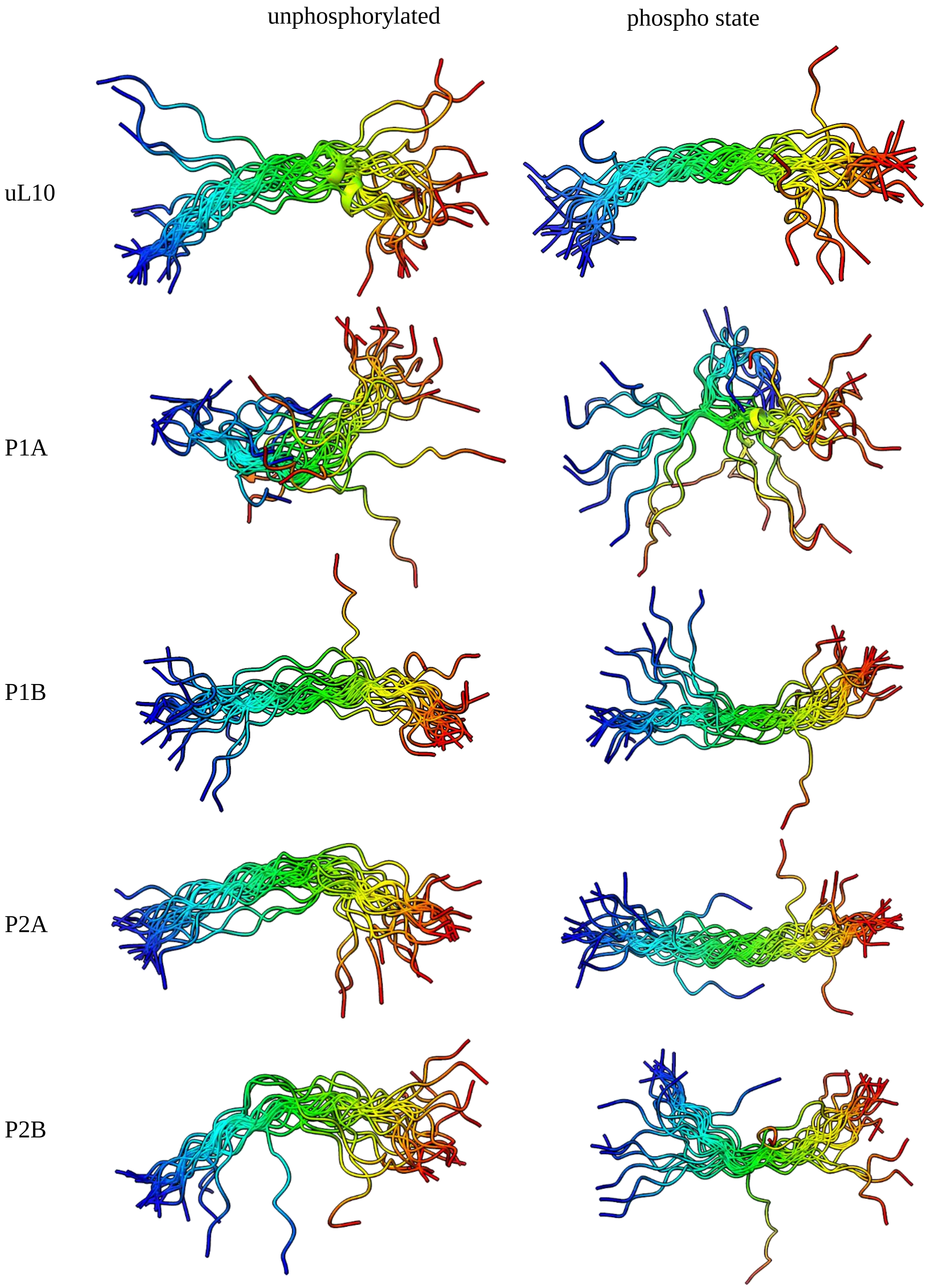

Appendix Figure S7. MD simulation of ribosomal P-stalk CTDs belonging to yeast P-stalk proteins. Representative structures of the most populated clusters from the MD simulations of the C-termini of yeast P-proteins (uL10, P1A, P1B, P2A and P2B) - wild-type (left side) and phospho-state (right side).

Appendix Figure. S8

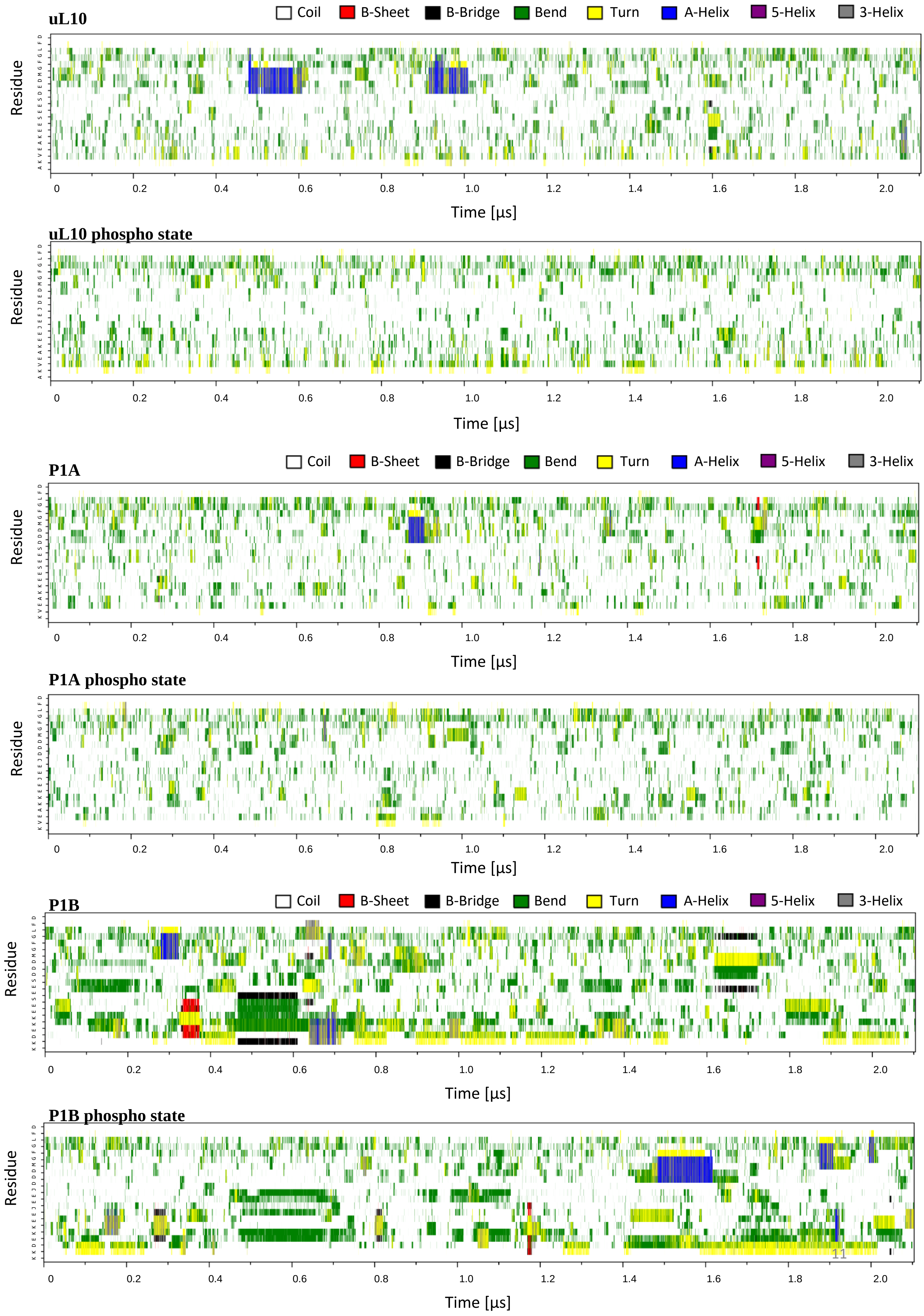

Appendix Figure. S8

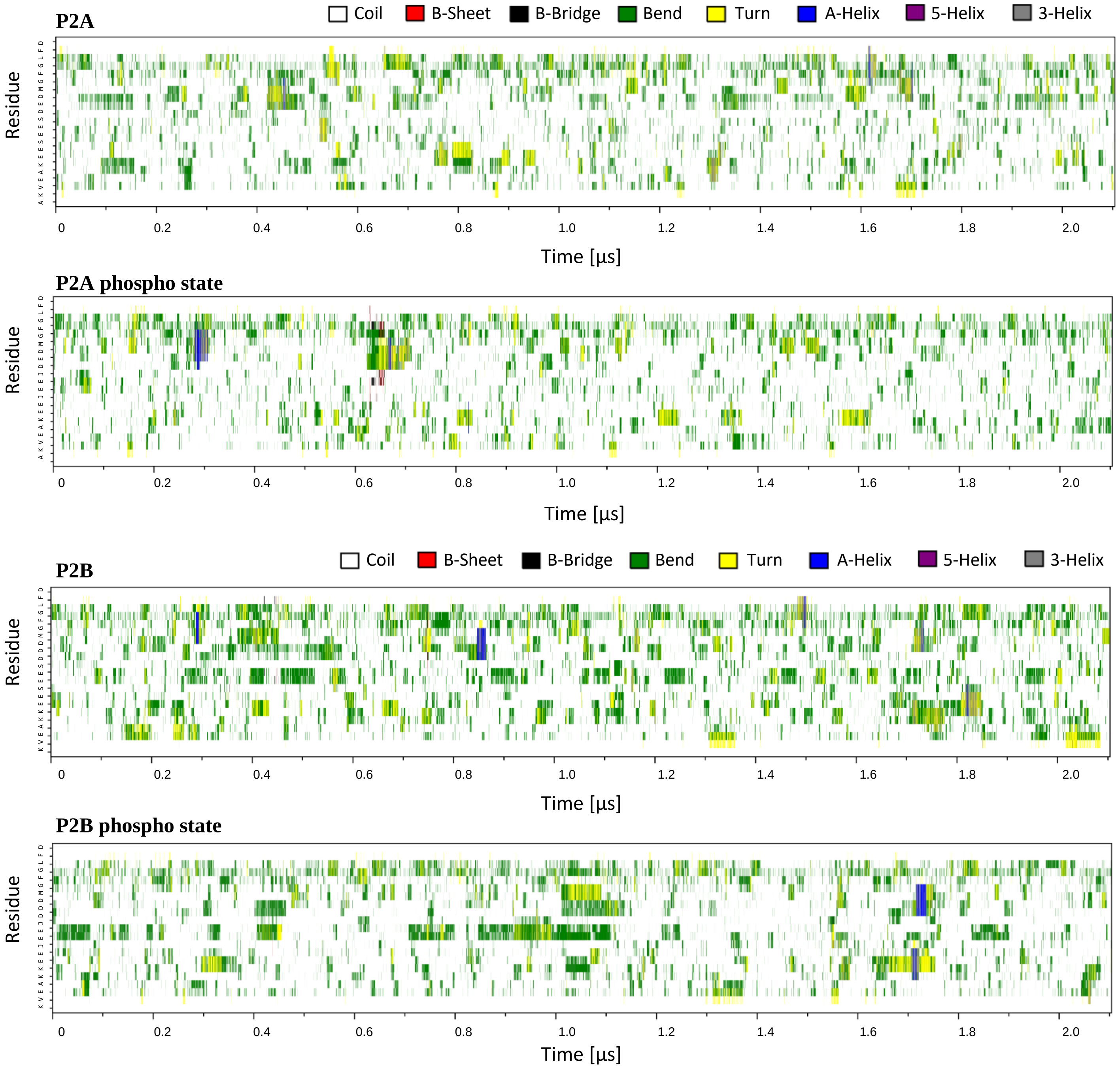

Figure S8. MD simulation of the linear polypeptide chain. The graph shows the occurrence of secondary structures versus time for the C-terminal peptides of the yeast P-proteins (uL10, P1A, P1B, P2A, P2B) folding for the unphosphorylated and phospho states. The structures have been coloured as indicated in the legend above. X - time of simulation, Y - position of individual amino acid residues.

Appendix Figure. S9

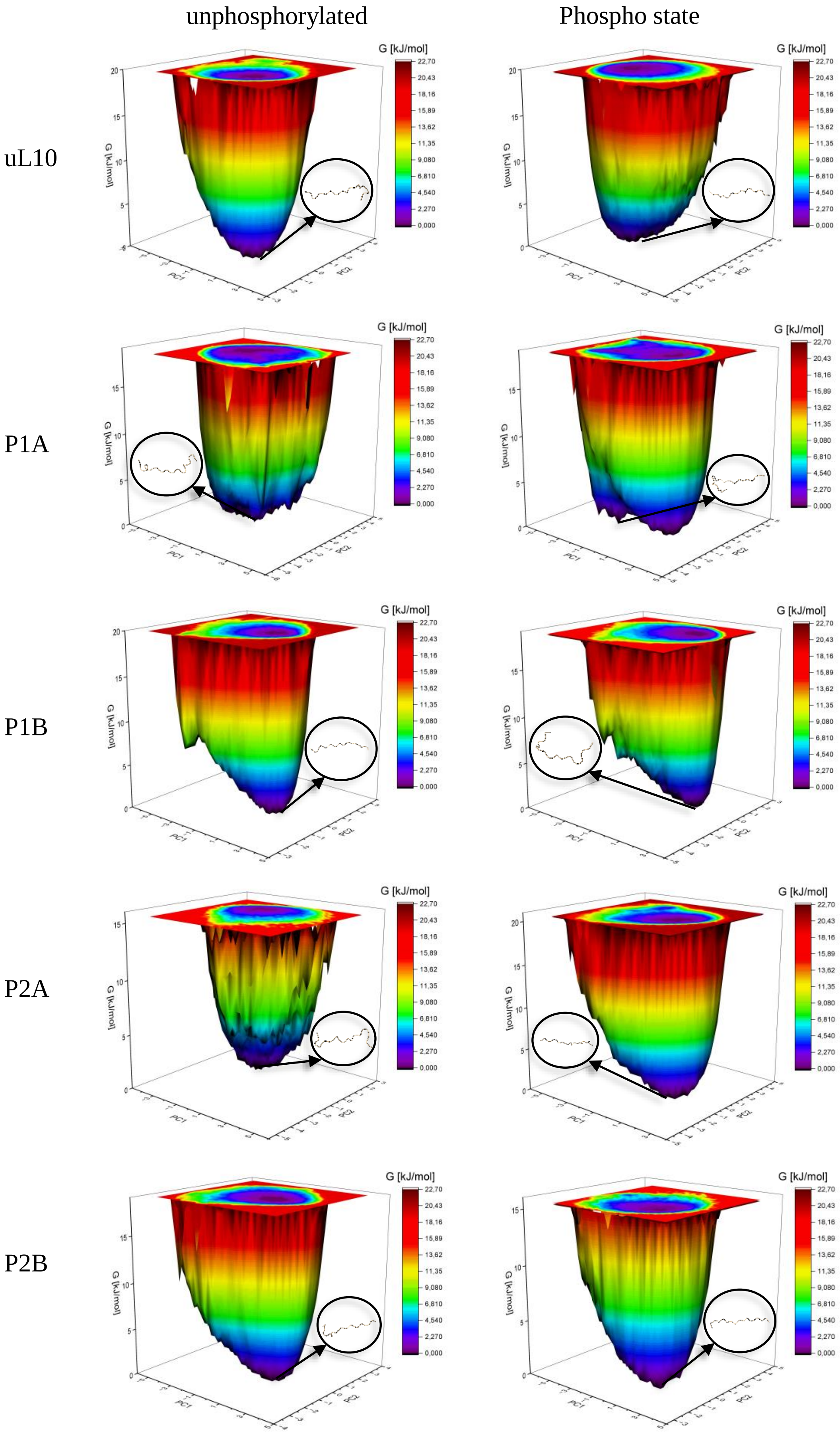

Appendix Figure S9. Mapping of the free energy landscapes of the yeast P-proteins (ul10, P1A, P1B, P2A, P2B) C-terminal peptides. Wild type (left) and phospho state (right) from S. cerevisiae; inset - representative lowest-energy structures sampled during MD simulation.

Appendix Figure. S10

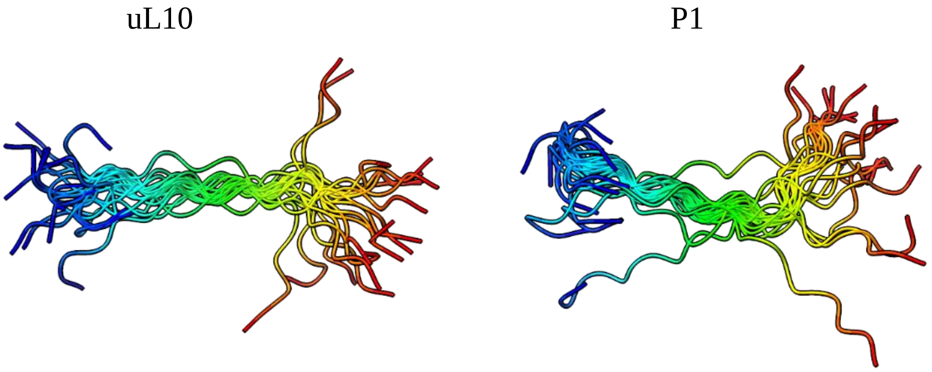

Appendix Figure S10. MD simulation of archaeal ribosomal P-stalk CTDs. Representative structures of the most populated clusters from the MD simulations of the C-termini of archaeal P proteins (uL10, P1). Rainbow colours represent the position of the amino acid within the analysed C-terminal polypeptide chain: N-terminal (blue) to C-terminal (red).

Appendix Figure. S11

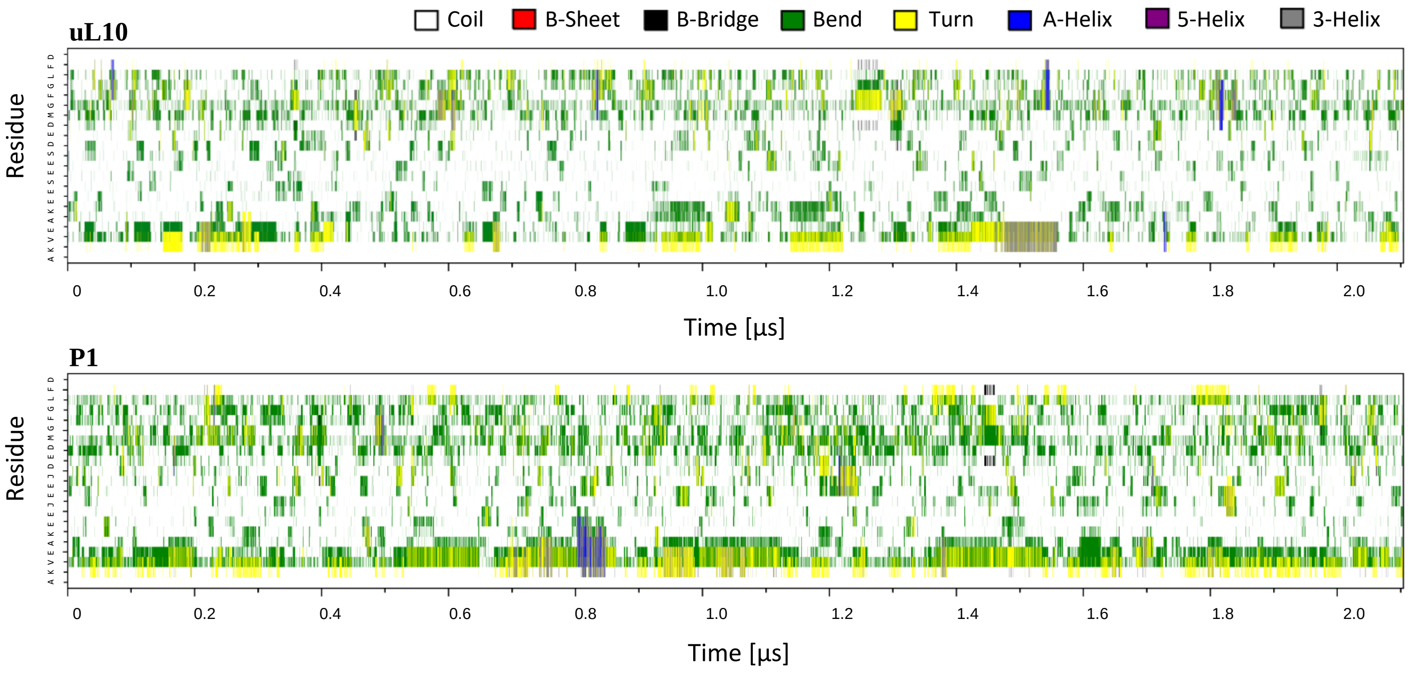

Appendix Figure S11. MD simulation of linear polypeptide chains for Archea proteins. The graph shows the occurrence of secondary structures versus time for the folding of the C-terminal peptides of the archaeal P-proteins (uL10, P1). The structures have been marked with individual colours as indicated in the legend above. X - time of simulation, Y - position of individual amino acid residues.

Appendix Figure. S12

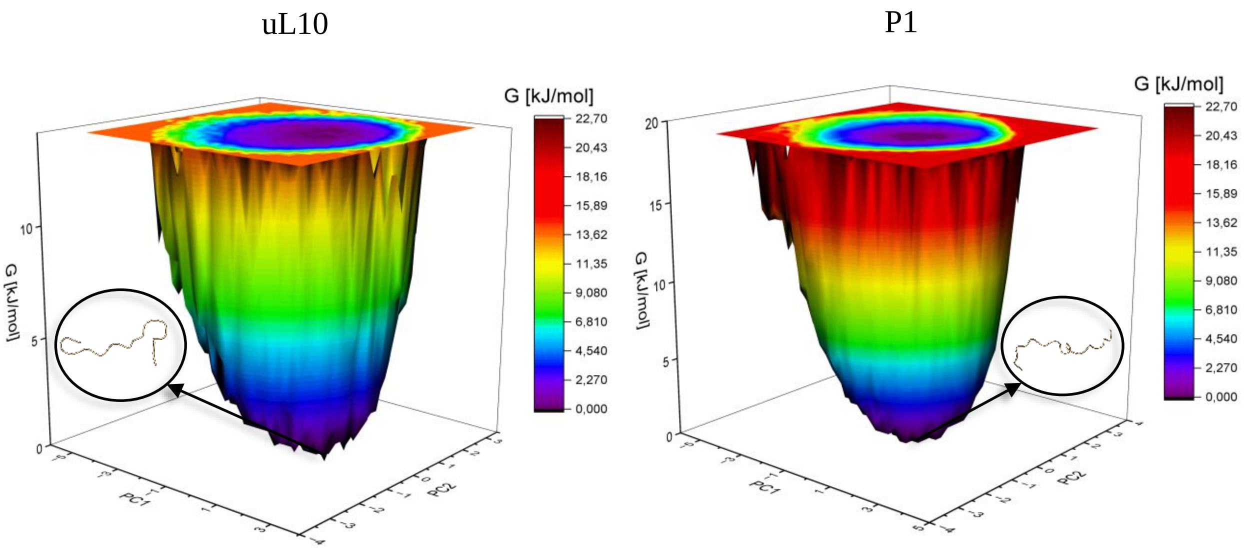

Appendix Figure S12. Free energy landscape mapping of the C-terminal peptides of Archaea P-proteins (uL10, P1). Inset - representative lowest-energy structures sampled during MD simulation.

Appendix Figure. S13

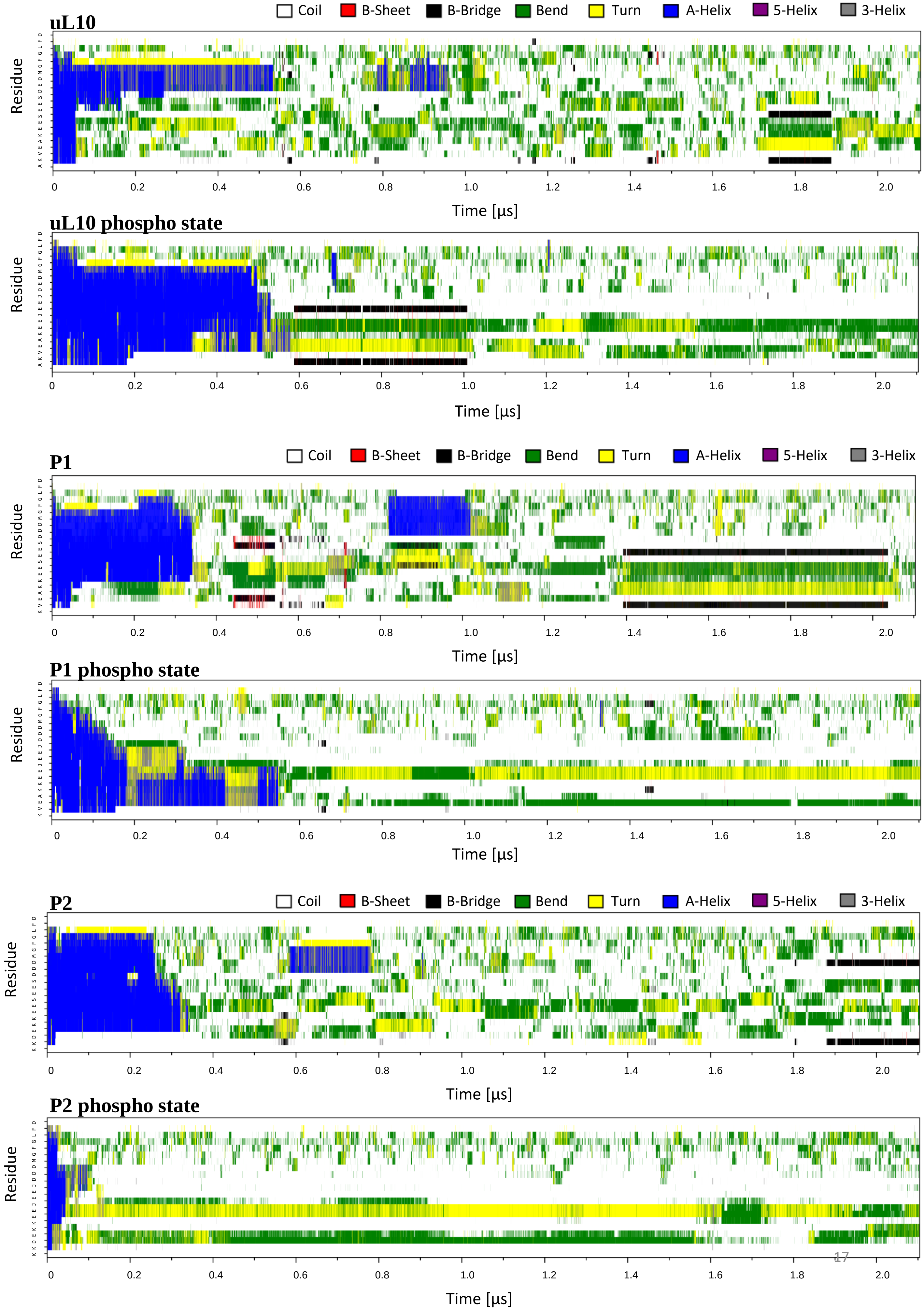

Appendix Figure S13. MD simulation of α-helix stability structures of the human P-proteins (uL10, P1, P2) C-termini peptides in dephospho and phospho state. The structures were marked with individual colour as marked in the presented legend. On the X – time of simulation, Y – position of individual amino acid residues.

Appendix Figure. S14

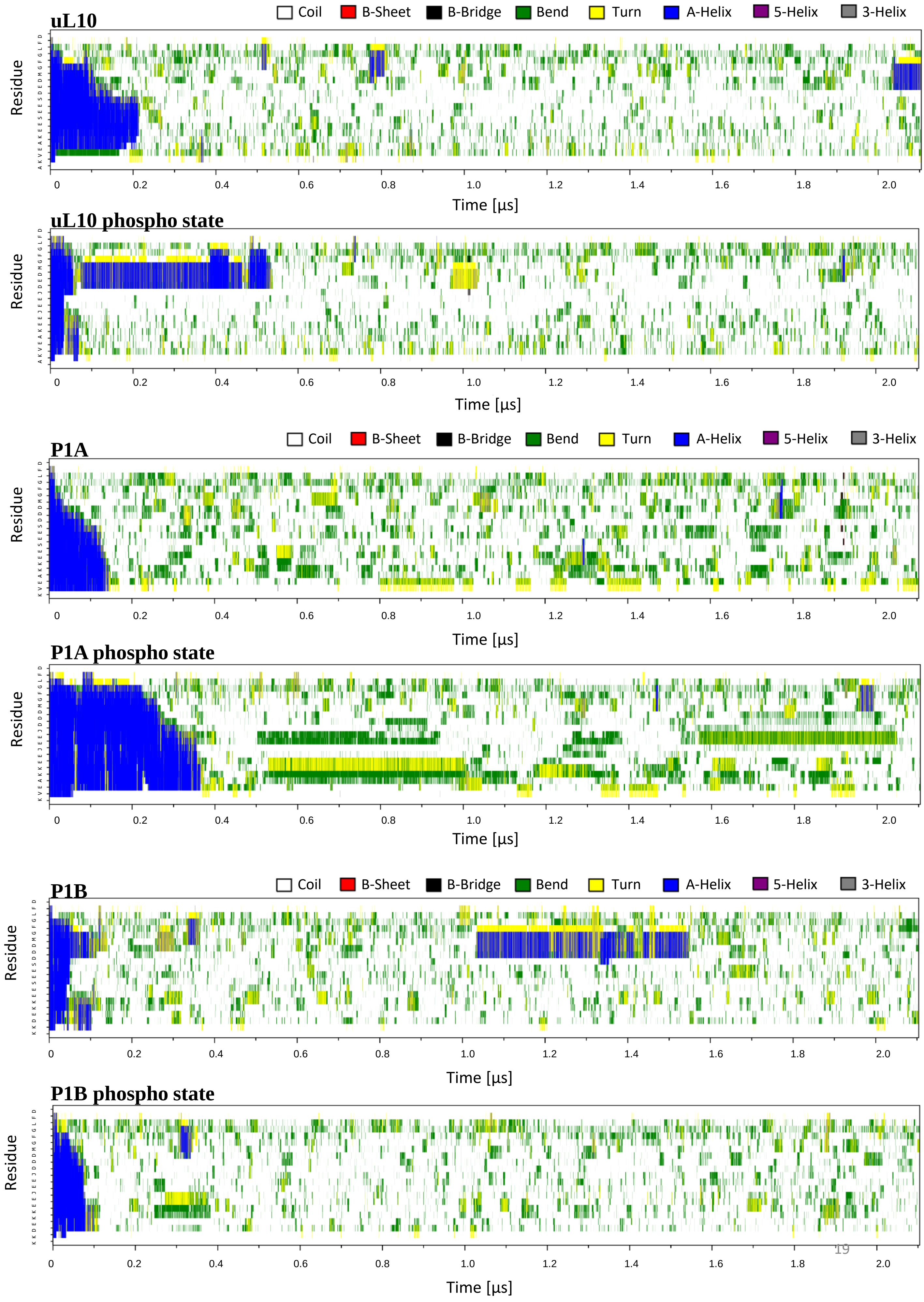

Appendix Figure. S14

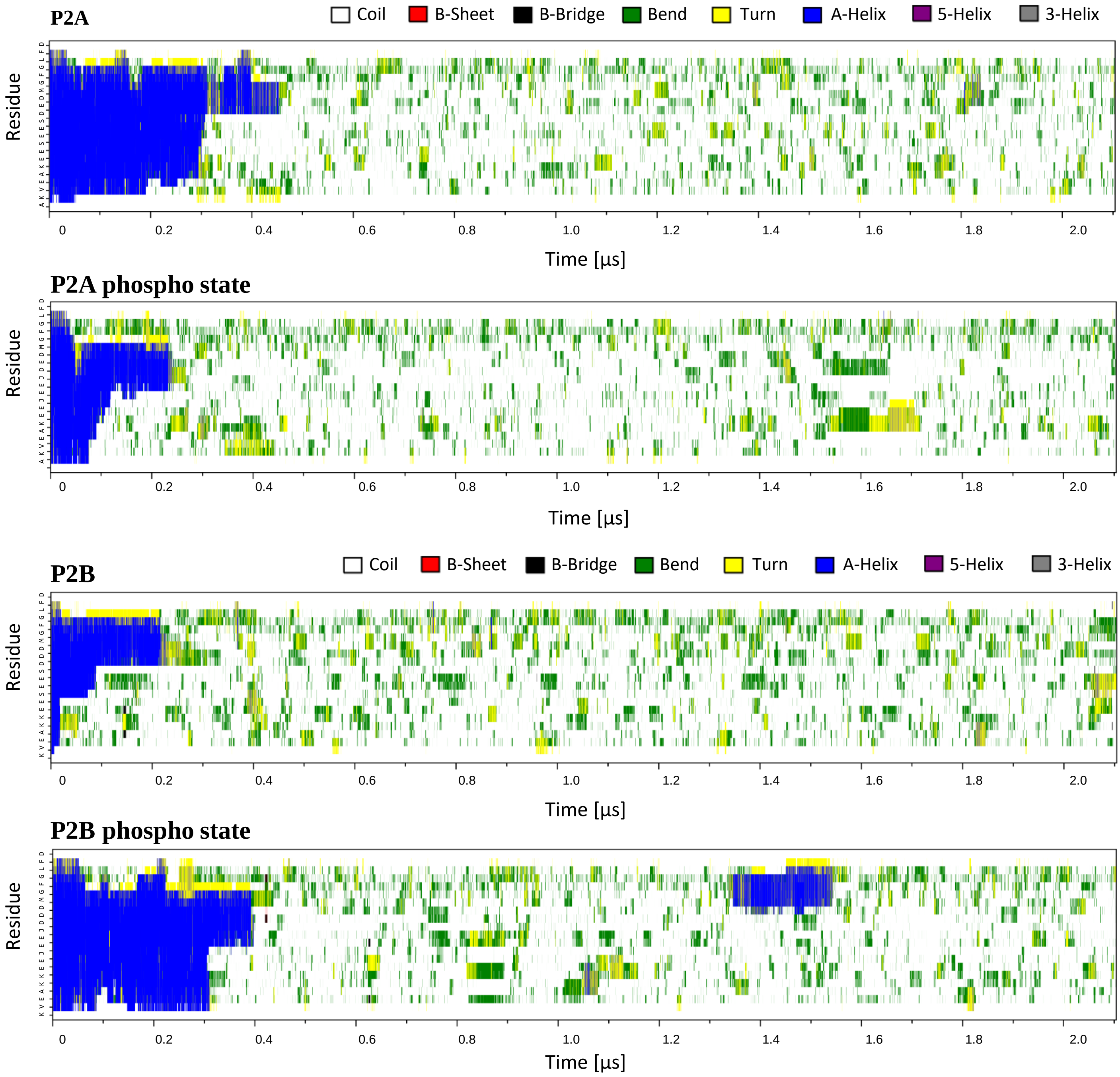

Appendix Figure S14. MD simulation of α-helix stability structures of P-proteins from yeast (uL10, P1A, P1B, P2A, P2B) C-termini peptides in dephospho and phospho state; the structures were marked with individual color as marked in the presented legend. On the X – time of simulation, Y – position of individual amino acid residues.

Appendix Figure. S15

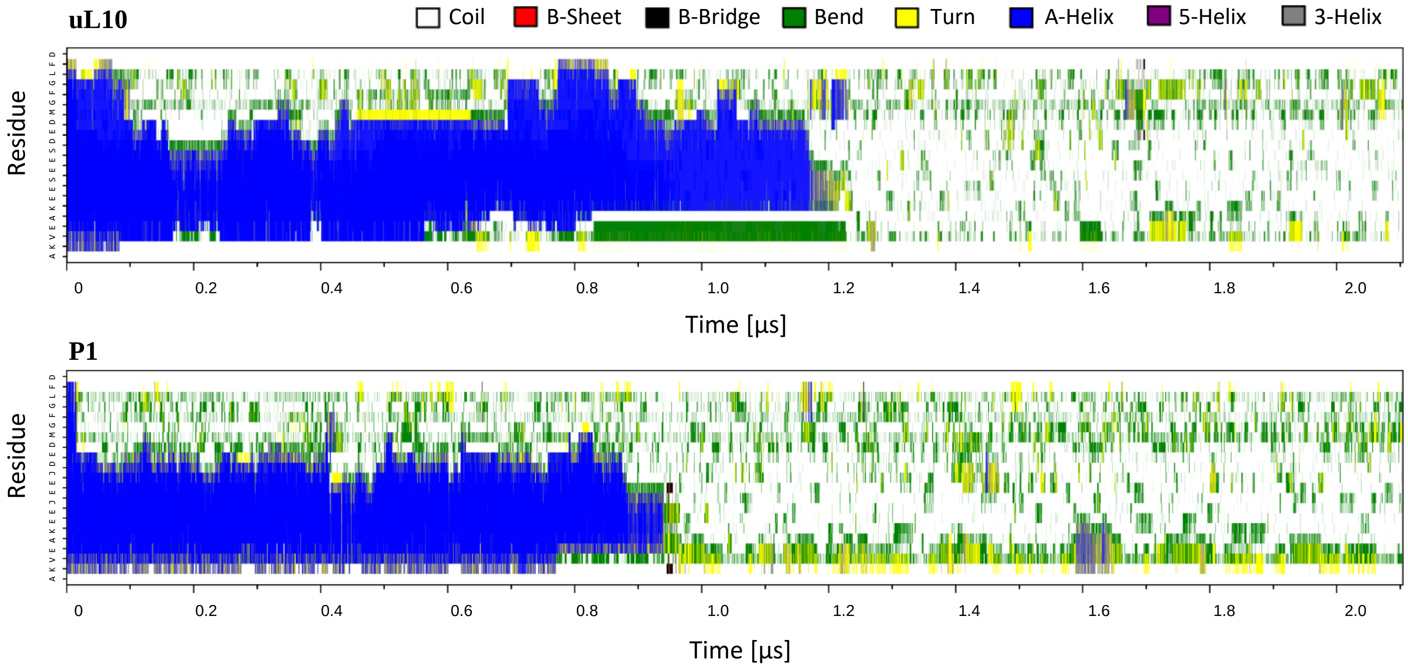

Appendix Figure S15. MD simulation of α-helix stability structures of P-proteins from Archaea (uL10, P1) C-termini peptides; the structures were marked with individual color as marked in the legend presented. On the X – time of simulation, Y – position of individual amino acid residues.

Appendix Figure. S16

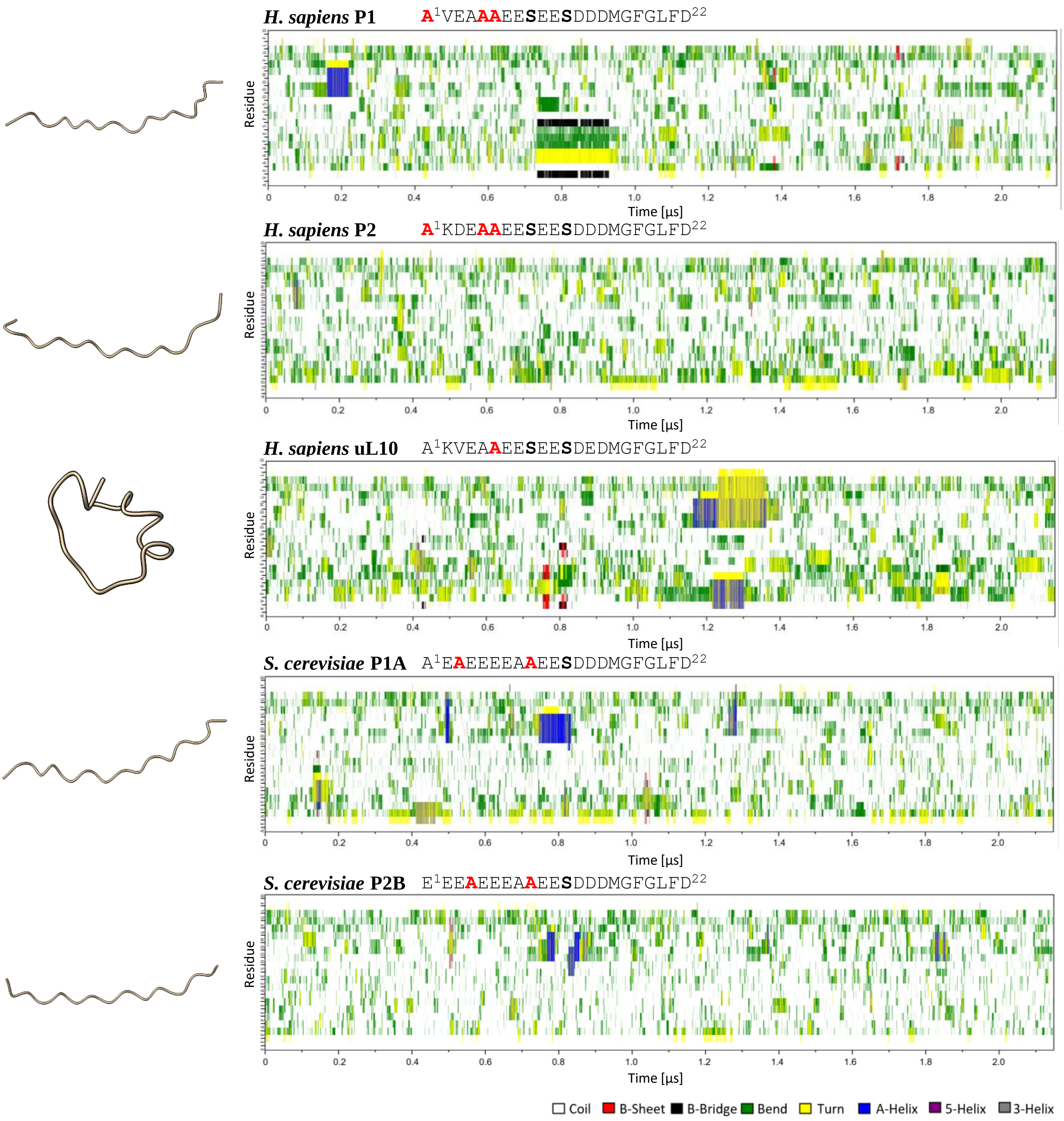

Appendix Figure S16. MD simulations of lysine-to-alanine mutant forms of the P-stalk C-terminal peptides. Left: the medoid structures of the most populated cluster for each MD simulation, identified using k-medoids clustering with a 3 Å cutoff, are shown. These medoid structures represent the central conformations of the major conformational states sampled during the simulations, highlighting the predominant structural motifs and variations within the mutant ensemble. Right: plots displaying the secondary structure evolution of the P-stalk proteins over the MD simulation time course, following the substitution of lysine residues with alanine. The plots show the structural stability and dynamics of the mutant peptides. Above, the amino acid sequences of the mutant peptides with alanine (in red) are shown, representing the substitution K to A.

Appendix Table. S1

Apendix Table S1. List of the proteins detected by mass spectrometry in WT (left) and SDPM (right) ribosomal samples. Proteins are sorted according to their numbering in the ribosomal nomenclature (60S and 40S). For each of the detected protein, theoretical molecular weight and length of protein are indicated. The mass spectrometry results are presented with the identification probability, exclusive unique spectrum counts and percentage of sequence coverage.							
Biological sample name	Protein name	Alternate IDs	Protein molecular weight (Da)	Protein identification probability	Exclusive unique spectrum count	Percentage sequence coverage	Length (residues)
WT Ribosomes (BY4741)	60S ribosomal protein L1-A OS=Saccharomyces cerevisiae (strain ATCC 204508 / S288c) GN=RPL1A PE=1 SV=1	RPL1A,RPL1B	24 486,3	100.00%	25	55.30%	217
WT Ribosomes (BY4741)	60S ribosomal protein L2-A OS=Saccharomyces cerevisiae (strain ATCC 204508 / S288c) GN=RPL2A PE=1 SV=1	RPL2A,RPL2B	27 408,9	100.00%	35	66.90%	254
WT Ribosomes (BY4741)	60S ribosomal protein L3 OS=Saccharomyces cerevisiae (strain ATCC 204508 / S288c) GN=RPL3 PE=1 SV=4	RPL3	43 757,9	100.00%	55	65.60%	387
WT Ribosomes (BY4741)	60S ribosomal protein L4-A OS=Saccharomyces cerevisiae (strain ATCC 204508 / S288c) GN=RPL4A PE=1 SV=4	RPL4A	39 092,0	100.00%	38	65.20%	362
WT Ribosomes (BY4741)	60S ribosomal protein L5 OS=Saccharomyces cerevisiae (strain ATCC 204508 / S288c) GN=RPL5 PE=1 SV=4	RPL5	33 716,1	100.00%	23	50.50%	297
WT Ribosomes (BY4741)	60S ribosomal protein L6-A OS=Saccharomyces cerevisiae (strain ATCC 204508 / S288c) GN=RPL6A PE=1 SV=2	RPL6A	19 962,1	100.00%	33	78.40%	176
WT Ribosomes (BY4741)	60S ribosomal protein L6-B OS=Saccharomyces cerevisiae (strain ATCC 204508 / S288c) GN=RPL6B PE=1 SV=4	RPL6B	19 986,9	100.00%	11	77.30%	176
WT Ribosomes (BY4741)	60S ribosomal protein L7-B OS=Saccharomyces cerevisiae (strain ATCC 204508 / S288c) GN=RPL7B PE=1 SV=3	RPL7B	27 697,8	100.00%	25	52.90%	244
WT Ribosomes (BY4741)	60S ribosomal protein L8-B OS=Saccharomyces cerevisiae (strain ATCC 204508 / S288c) GN=RPL8B PE=1 SV=3	RPL8B	28 112,8	100.00%	39	71.90%	256
WT Ribosomes (BY4741)	60S ribosomal protein L8-A OS=Saccharomyces cerevisiae (strain ATCC 204508 / S288c) GN=RPL8A PE=1 SV=4	RPL8A	28 125,5	100.00%	6	67.60%	256
WT Ribosomes (BY4741)	60S ribosomal protein L9-A OS=Saccharomyces cerevisiae (strain ATCC 204508 / S288c) GN=RPL9A PE=1 SV=2	RPL9A	21 568,8	100.00%	20	55.00%	191
WT Ribosomes (BY4741)	60S ribosomal protein L10 OS=Saccharomyces cerevisiae (strain ATCC 204508 / S288c) GN=RPL10 PE=1 SV=1	RPL10	25 362,1	100.00%	35	72.90%	221
WT Ribosomes (BY4741)	60S ribosomal protein L11-A OS=Saccharomyces cerevisiae (strain ATCC 204508 / S288c) GN=RPL11A PE=1 SV=2	RPL11A,RPL11B	19 750,0	100.00%	13	39.70%	174
WT Ribosomes (BY4741)	60S ribosomal protein L12-A OS=Saccharomyces cerevisiae (strain ATCC 204508 / S288c) GN=RPL12A PE=1 SV=1	RPL12A,RPL12B	17 823,2	100.00%	17	77.00%	165
WT Ribosomes (BY4741)	60S ribosomal protein L13-A OS=Saccharomyces cerevisiae (strain ATCC 204508 / S288c) GN=RPL13A PE=1 SV=1	RPL13A	22 554,5	100.00%	29	61.30%	199
WT Ribosomes (BY4741)	60S ribosomal protein L14-A OS=Saccharomyces cerevisiae (strain ATCC 204508 / S288c) GN=RPL14A PE=1 SV=1	RPL14A	15 167,2	100.00%	24	66.70%	138
WT Ribosomes (BY4741)	60S ribosomal protein L14-B OS=Saccharomyces cerevisiae (strain ATCC 204508 / S288c) GN=RPL14B PE=1 SV=1	RPL14B	15 153,2	100.00%	2	65.90%	138
WT Ribosomes (BY4741)	60S ribosomal protein L15-A OS=Saccharomyces cerevisiae (strain ATCC 204508 / S288c) GN=RPL15A PE=1 SV=3	RPL15A	24 422,6	100.00%	18	52.50%	204
WT Ribosomes (BY4741)	60S ribosomal protein L16-B OS=Saccharomyces cerevisiae (strain ATCC 204508 / S288c) GN=RPL16B PE=1 SV=3	RPL16B	22 249,9	100.00%	17	56.10%	198
WT Ribosomes (BY4741)	60S ribosomal protein L16-A OS=Saccharomyces cerevisiae (strain ATCC 204508 / S288c) GN=RPL16A PE=1 SV=3	RPL16A	22 201,6	100.00%	30	57.30%	199
WT Ribosomes (BY4741)	60S ribosomal protein L17-B OS=Saccharomyces cerevisiae (strain ATCC 204508 / S288c) GN=RPL17B PE=1 SV=2	RPL17B	20 551,6	100.00%	27	65.20%	184
WT Ribosomes (BY4741)	60S ribosomal protein L18-A OS=Saccharomyces cerevisiae (strain ATCC 204508 / S288c) GN=RPL18A PE=1 SV=1	RPL18A,RPL18B	20 563,7	100.00%	15	48.40%	186
WT Ribosomes (BY4741)	60S ribosomal protein L19-A OS=Saccharomyces cerevisiae (strain ATCC 204508 / S288c) GN=RPL19A PE=1 SV=1	RPL19A,RPL19B	21 704,6	100.00%	30	52.40%	189
WT Ribosomes (BY4741)	60S ribosomal protein L20-A OS=Saccharomyces cerevisiae (strain ATCC 204508 / S288c) GN=RPL20A PE=1 SV=1	RPL20A,RPL20B	20 437,1	100.00%	29	73.80%	172
WT Ribosomes (BY4741)	60S ribosomal protein L21-A OS=Saccharomyces cerevisiae (strain ATCC 204508 / S288c) GN=RPL21A PE=1 SV=1	RPL21A	18 242,0	100.00%	24	72.50%	160
WT Ribosomes (BY4741)	60S ribosomal protein L22-A OS=Saccharomyces cerevisiae (strain ATCC 204508 / S288c) GN=RPL22A PE=1 SV=3	RPL22A	13 693,4	100.00%	5	54.50%	121
WT Ribosomes (BY4741)	60S ribosomal protein L23-A OS=Saccharomyces cerevisiae (strain ATCC 204508 / S288c) GN=RPL23A PE=1 SV=1	RPL23A,RPL23B	14 473,1	100.00%	17	56.20%	137
WT Ribosomes (BY4741)	60S ribosomal protein L24-A OS=Saccharomyces cerevisiae (strain ATCC 204508 / S288c) GN=RPL24A PE=1 SV=1	RPL24A	17 614,4	100.00%	17	42.60%	155
WT Ribosomes (BY4741)	60S ribosomal protein L25 OS=Saccharomyces cerevisiae (strain ATCC 204508 / S288c) GN=RPL25 PE=1 SV=4	RPL25	15 758,2	100.00%	20	67.60%	142
WT Ribosomes (BY4741)	60S ribosomal protein L26-A OS=Saccharomyces cerevisiae (strain ATCC 204508 / S288c) GN=RPL26A PE=1 SV=3	RPL26A	14 234,3	100.00%	4	69.30%	127
WT Ribosomes (BY4741)	60S ribosomal protein L26-B OS=Saccharomyces cerevisiae (strain ATCC 204508 / S288c) GN=RPL26B PE=1 SV=2	RPL26B	14 235,3	100.00%	33	69.30%	127
WT Ribosomes (BY4741)	60S ribosomal protein L27-A OS=Saccharomyces cerevisiae (strain ATCC 204508 / S288c) GN=RPL27A PE=1 SV=1	RPL27A	15 531,2	100.00%	30	70.60%	136
WT Ribosomes (BY4741)	60S ribosomal protein L28 OS=Saccharomyces cerevisiae (strain ATCC 204508 / S288c) GN=RPL28 PE=1 SV=3	RPL28	16 721,9	100.00%	12	38.30%	149
WT Ribosomes (BY4741)	60S ribosomal protein L30 OS=Saccharomyces cerevisiae (strain ATCC 204508 / S288c) GN=RPL30 PE=1 SV=3	RPL30	11 416,0	100.00%	25	78.10%	105
WT Ribosomes (BY4741)	60S ribosomal protein L31-A OS=Saccharomyces cerevisiae (strain ATCC 204508 / S288c) GN=RPL31A PE=1 SV=1	RPL31A	12 953,2	100.00%	18	55.80%	113
WT Ribosomes (BY4741)	60S ribosomal protein L32 OS=Saccharomyces cerevisiae (strain ATCC 204508 / S288c) GN=RPL32 PE=1 SV=1	RPL32	14 771,8	100.00%	17	54.60%	130
WT Ribosomes (BY4741)	60S ribosomal protein L33-A OS=Saccharomyces cerevisiae (strain ATCC 204508 / S288c) GN=RPL33A PE=1 SV=3	RPL33A	12 154,2	100.00%	13	57.00%	107
WT Ribosomes (BY4741)	60S ribosomal protein L33-B OS=Saccharomyces cerevisiae (strain ATCC 204508 / S288c) GN=RPL33B PE=1 SV=2	RPL33B	12 168,2	100.00%	5	57.00%	107
WT Ribosomes (BY4741)	60S ribosomal protein L34-B OS=Saccharomyces cerevisiae (strain ATCC 204508 / S288c) GN=RPL34B PE=1 SV=1	RPL34A,RPL34B	13 639,2	100.00%	4	24.00%	121
WT Ribosomes (BY4741)	60S ribosomal protein L35-A OS=Saccharomyces cerevisiae (strain ATCC 204508 / S288c) GN=RPL35A PE=1 SV=1	RPL35A,RPL35B	13 910,2	100.00%	28	61.70%	120
WT Ribosomes (BY4741)	60S ribosomal protein L36-B OS=Saccharomyces cerevisiae (strain ATCC 204508 / S288c) GN=RPL36B PE=1 SV=3	RPL36B	11 135,9	100.00%	11	50.00%	100
WT Ribosomes (BY4741)	60S ribosomal protein L36-A OS=Saccharomyces cerevisiae (strain ATCC 204508 / S288c) GN=RPL36A PE=1 SV=3	RPL36A	11 124,9	100.00%	3	50.00%	100
WT Ribosomes (BY4741)	60S ribosomal protein L37-A OS=Saccharomyces cerevisiae (strain ATCC 204508 / S288c) GN=RPL37A PE=1 SV=2	RPL37A	9 850,4	100.00%	10	47.70%	88
WT Ribosomes (BY4741)	60S ribosomal protein L37-B OS=Saccharomyces cerevisiae (strain ATCC 204508 / S288c) GN=RPL37B PE=1 SV=2	RPL37B	9 868,3	100.00%	5	46.60%	88

WT Ribosomes (BY4741)	60S ribosomal protein L38 OS=Saccharomyces cerevisiae (strain ATCC 204508 / S288c) GN=RPL38 PE=1 SV=1	RPL38	8 827,2	100.00%	19	62.80%	78
WT Ribosomes (BY4741)	60S ribosomal protein L39 OS=Saccharomyces cerevisiae (strain ATCC 204508 / S288c) GN=RPL39 PE=1 SV=3	RPL39	6 342,0	100.00%	4	21.60%	51
WT Ribosomes (BY4741)	Ubiquitin-60S ribosomal protein L40 OS=Saccharomyces cerevisiae (strain ATCC 204508 / S288c) GN=RPL40A PE=1 SV=1	RPL40A,RPL40B	14 554,8	100.00%	5	35.90%	128
WT Ribosomes (BY4741)	60S ribosomal protein L42-A OS=Saccharomyces cerevisiae (strain ATCC 204508 / S288c) GN=RPL42A PE=1 SV=1	RPL42A,RPL42B	12 211,5	100.00%	13	44.30%	106
WT Ribosomes (BY4741)	60S ribosomal protein L43-A OS=Saccharomyces cerevisiae (strain ATCC 204508 / S288c) GN=RPL43A PE=1 SV=1	RPL43A,RPL43B	10 090,8	100.00%	15	59.80%	92
WT Ribosomes (BY4741)	60S acidic ribosomal protein P1-alpha OS=Saccharomyces cerevisiae (strain ATCC 204508 / S288c) GN=RPP1A PE=1 SV=4	RPP1A	10 908,3	100.00%	2	28.30%	106
WT Ribosomes (BY4741)	60S acidic ribosomal protein P2-beta OS=Saccharomyces cerevisiae (strain ATCC 204508 / S288c) GN=RPP2B PE=1 SV=2	RPP2B	11 050,3	100.00%	16	83.60%	110
WT Ribosomes (BY4741)	60S acidic ribosomal protein P2-alpha OS=Saccharomyces cerevisiae (strain ATCC 204508 / S288c) GN=RPP2A PE=1 SV=1	RPP2A	10 746,2	100.00%	10	56.60%	106
WT Ribosomes (BY4741)	60S acidic ribosomal protein P1-beta OS=Saccharomyces cerevisiae (strain ATCC 204508 / S288c) GN=RPP1B PE=1 SV=3	RPP1B	10 668,1	100.00%	2	64.20%	106
WT Ribosomes (BY4741)	60S acidic ribosomal protein P0 OS=Saccharomyces cerevisiae (strain ATCC 204508 / S288c) GN=RPP0 PE=1 SV=2	RPP0	33 717,4	100.00%	11	34.00%	312
WT Ribosomes (BY4741)	40S ribosomal protein S0-A OS=Saccharomyces cerevisiae (strain YJM789) GN=RPS0A PE=3 SV=1	RPS0A	28 024,2	100.00%	19	61.10%	252
WT Ribosomes (BY4741)	40S ribosomal protein S1-B OS=Saccharomyces cerevisiae (strain YJM789) GN=RPS1B PE=3 SV=1	RPS1B	28 812,9	100.00%	37	69.80%	255
WT Ribosomes (BY4741)	40S ribosomal protein S1-A OS=Saccharomyces cerevisiae (strain YJM789) GN=RPS1A PE=3 SV=1	RPS1A	28 743,8	100.00%	3	60.80%	255
WT Ribosomes (BY4741)	40S ribosomal protein S2 OS=Saccharomyces cerevisiae (strain ATCC 204508 / S288c) GN=RPS2 PE=1 SV=3	RPS2	27 450,2	100.00%	26	57.90%	254
WT Ribosomes (BY4741)	40S ribosomal protein S3 OS=Saccharomyces cerevisiae (strain ATCC 204508 / S288c) GN=RPS3 PE=1 SV=5	RPS3	26 503,0	100.00%	41	70.40%	240
WT Ribosomes (BY4741)	40S ribosomal protein S4-A OS=Saccharomyces cerevisiae (strain ATCC 204508 / S288c) GN=RPS4A PE=1 SV=1	RPS4A,RPS4B	29 411,2	100.00%	40	60.90%	261
WT Ribosomes (BY4741)	40S ribosomal protein S5 OS=Saccharomyces cerevisiae (strain ATCC 204508 / S288c) GN=RPS5 PE=1 SV=3	RPS5	25 038,5	100.00%	31	44.00%	225
WT Ribosomes (BY4741)	40S ribosomal protein S6-A OS=Saccharomyces cerevisiae (strain ATCC 204508 / S288c) GN=RPS6A PE=1 SV=1	RPS6A,RPS6B	26 997,2	100.00%	36	61.00%	236
WT Ribosomes (BY4741)	40S ribosomal protein S7-B OS=Saccharomyces cerevisiae (strain ATCC 204508 / S288c) GN=RPS7B PE=1 SV=1	RPS7B	21 634,7	100.00%	38	75.30%	190
WT Ribosomes (BY4741)	40S ribosomal protein S7-A OS=Saccharomyces cerevisiae (strain ATCC 204508 / S288c) GN=RPS7A PE=1 SV=4	RPS7A	21 622,7	100.00%	18	83.20%	190
WT Ribosomes (BY4741)	40S ribosomal protein S8-A OS=Saccharomyces cerevisiae (strain ATCC 204508 / S288c) GN=RPS8A PE=1 SV=1	RPS8A,RPS8B	22 490,4	100.00%	21	50.00%	200
WT Ribosomes (BY4741)	40S ribosomal protein S9-B OS=Saccharomyces cerevisiae (strain ATCC 204508 / S288c) GN=RPS9B PE=1 SV=4	RPS9B	22 299,7	100.00%	27	54.90%	195
WT Ribosomes (BY4741)	40S ribosomal protein S10-B OS=Saccharomyces cerevisiae (strain ATCC 204508 / S288c) GN=RPS10B PE=1 SV=1	RPS10B	12 738,7	100.00%	18	62.90%	105
WT Ribosomes (BY4741)	40S ribosomal protein S11-A OS=Saccharomyces cerevisiae (strain ATCC 204508 / S288c) GN=RPS11A PE=1 SV=1	RPS11A,RPS11B	17 748,8	100.00%	25	69.20%	156
WT Ribosomes (BY4741)	40S ribosomal protein S12 OS=Saccharomyces cerevisiae (strain ATCC 204508 / S288c) GN=RPS12 PE=1 SV=1	RPS12	15 470,9	100.00%	13	46.90%	143
WT Ribosomes (BY4741)	40S ribosomal protein S13 OS=Saccharomyces cerevisiae (strain ATCC 204508 / S288c) GN=RPS13 PE=1 SV=3	RPS13	17 029,8	100.00%	12	56.30%	151
WT Ribosomes (BY4741)	40S ribosomal protein S14-B OS=Saccharomyces cerevisiae (strain ATCC 204508 / S288c) GN=RPS14B PE=1 SV=2	RPS14B	14 649,8	100.00%	29	76.80%	138
WT Ribosomes (BY4741)	40S ribosomal protein S15 OS=Saccharomyces cerevisiae (strain ATCC 204508 / S288c) GN=RPS15 PE=1 SV=1	RPS15	16 002,2	100.00%	17	45.10%	142
WT Ribosomes (BY4741)	40S ribosomal protein S16-A OS=Saccharomyces cerevisiae (strain ATCC 204508 / S288c) GN=RPS16A PE=1 SV=1	RPS16A,RPS16B	15 847,9	100.00%	23	75.50%	143
WT Ribosomes (BY4741)	40S ribosomal protein S17-A OS=Saccharomyces cerevisiae (strain ATCC 204508 / S288c) GN=RPS17A PE=1 SV=1	RPS17A,RPS17B	15 788,9	100.00%	34	75.00%	136
WT Ribosomes (BY4741)	40S ribosomal protein S18-A OS=Saccharomyces cerevisiae (strain ATCC 204508 / S288c) GN=RPS18A PE=1 SV=1	RPS18A,RPS18B	17 037,9	100.00%	28	71.90%	146
WT Ribosomes (BY4741)	40S ribosomal protein S19-B OS=Saccharomyces cerevisiae (strain ATCC 204508 / S288c) GN=RPS19B PE=1 SV=2	RPS19B	15 891,3	100.00%	17	59.00%	144
WT Ribosomes (BY4741)	40S ribosomal protein S20 OS=Saccharomyces cerevisiae (strain ATCC 204508 / S288c) GN=RPS20 PE=1 SV=3	RPS20	13 906,8	100.00%	18	71.90%	121
WT Ribosomes (BY4741)	40S ribosomal protein S21-B OS=Saccharomyces cerevisiae (strain ATCC 204508 / S288c) GN=RPS21B PE=1 SV=1	RPS21B	9 760,0	100.00%	8	75.90%	87
WT Ribosomes (BY4741)	40S ribosomal protein S22-A OS=Saccharomyces cerevisiae (strain ATCC 204508 / S288c) GN=RPS22A PE=1 SV=2	RPS22A,RPS22B	14 626,5	100.00%	18	78.50%	130
WT Ribosomes (BY4741)	40S ribosomal protein S23-A OS=Saccharomyces cerevisiae (strain ATCC 204508 / S288c) GN=RPS23A PE=1 SV=1	RPS23,RPS23A,RPS2	16 038,3	100.00%	16	51.70%	145
WT Ribosomes (BY4741)	40S ribosomal protein S24-A OS=Saccharomyces cerevisiae (strain ATCC 204508 / S288c) GN=RPS24A PE=1 SV=1	RPS24A,RPS24B	15 329,0	100.00%	26	62.20%	135
WT Ribosomes (BY4741)	40S ribosomal protein S25-B OS=Saccharomyces cerevisiae (strain ATCC 204508 / S288c) GN=RPS25B PE=1 SV=1	RPS25A,RPS25B	12 009,9	100.00%	18	58.30%	108
WT Ribosomes (BY4741)	40S ribosomal protein S26-A OS=Saccharomyces cerevisiae (strain ATCC 204508 / S288c) GN=RPS26A PE=1 SV=1	RPS26A	13 505,0	100.00%	7	33.60%	119
WT Ribosomes (BY4741)	40S ribosomal protein S27-A OS=Saccharomyces cerevisiae (strain ATCC 204508 / S288c) GN=RPS27A PE=1 SV=1	RPS27A,RPS27B	8 879,3	100.00%	8	43.90%	82
WT Ribosomes (BY4741)	40S ribosomal protein S28-A OS=Saccharomyces cerevisiae (strain ATCC 204508 / S288c) GN=RPS28A PE=1 SV=1	RPS28A	7 591,7	100.00%	14	67.20%	67
WT Ribosomes (BY4741)	40S ribosomal protein S29-B OS=Saccharomyces cerevisiae (strain ATCC 204508 / S288c) GN=RPS29B PE=1 SV=3	RPS29B	6 727,6	100.00%	7	69.60%	56
WT Ribosomes (BY4741)	40S ribosomal protein S29-A OS=Saccharomyces cerevisiae (strain ATCC 204508 / S288c) GN=RPS29A PE=1 SV=3	RPS29A	6 660,7	100.00%	9	69.60%	56
WT Ribosomes (BY4741)	40S ribosomal protein S30-A OS=Saccharomyces cerevisiae (strain ATCC 204508 / S288c) GN=RPS30A PE=1 SV=1	RPS30A,RPS30B	7 118,5	100.00%	8	33.30%	63
WT Ribosomes (BY4741)	Ubiquitin-40S ribosomal protein S31 OS=Saccharomyces cerevisiae (strain ATCC 204508 / S288c) GN=RPS31 PE=1 SV=3	RPS31	17 216,6	100.00%	18	59.20%	152

WT Ribosomes (BY4741)	Guanine nucleotide-binding protein subunit beta-like protein OS=Saccharomyces cerevisiae (strain ATCC 204508 / S288c) GN=	ASC1	34 805,9	100.00%	28	67.40%	319
SDPM Ribosomes	60S ribosomal protein L1-A OS=Saccharomyces cerevisiae (strain ATCC 204508 / S288c) GN=RPL1A PE=1 SV=1	RPL1A,RPL1B	24 486,3	100.00%	18	53.90%	217
SDPM Ribosomes	60S ribosomal protein L2-A OS=Saccharomyces cerevisiae (strain ATCC 204508 / S288c) GN=RPL2A PE=1 SV=1	RPL2A,RPL2B	27 408,9	100.00%	28	55.10%	254
SDPM Ribosomes	60S ribosomal protein L3 OS=Saccharomyces cerevisiae (strain ATCC 204508 / S288c) GN=RPL3 PE=1 SV=4	RPL3	43 757,9	100.00%	38	58.10%	387
SDPM Ribosomes	60S ribosomal protein L4-A OS=Saccharomyces cerevisiae (strain ATCC 204508 / S288c) GN=RPL4A PE=1 SV=4	RPL4A	39 092,0	100.00%	20	50.60%	362
SDPM Ribosomes	60S ribosomal protein L5 OS=Saccharomyces cerevisiae (strain ATCC 204508 / S288c) GN=RPL5 PE=1 SV=4	RPL5	33 716,1	100.00%	19	60.30%	297
SDPM Ribosomes	60S ribosomal protein L6-A OS=Saccharomyces cerevisiae (strain ATCC 204508 / S288c) GN=RPL6A PE=1 SV=2	RPL6A	19 962,1	100.00%	7	72.70%	176
SDPM Ribosomes	60S ribosomal protein L6-B OS=Saccharomyces cerevisiae (strain ATCC 204508 / S288c) GN=RPL6B PE=1 SV=4	RPL6B	19 986,9	100.00%	18	72.70%	176
SDPM Ribosomes	60S ribosomal protein L7-A OS=Saccharomyces cerevisiae (strain ATCC 204508 / S288c) GN=RPL7A PE=1 SV=3	RPL7A	27 639,6	100.00%	22	60.70%	244
SDPM Ribosomes	60S ribosomal protein L8-B OS=Saccharomyces cerevisiae (strain ATCC 204508 / S288c) GN=RPL8B PE=1 SV=3	RPL8B	28 112,8	100.00%	29	71.90%	256
SDPM Ribosomes	60S ribosomal protein L8-A OS=Saccharomyces cerevisiae (strain ATCC 204508 / S288c) GN=RPL8A PE=1 SV=4	RPL8A	28 125,5	100.00%	7	71.90%	256
SDPM Ribosomes	60S ribosomal protein L9-A OS=Saccharomyces cerevisiae (strain ATCC 204508 / S288c) GN=RPL9A PE=1 SV=2	RPL9A	21 568,8	100.00%	18	63.90%	191
SDPM Ribosomes	60S ribosomal protein L10 OS=Saccharomyces cerevisiae (strain ATCC 204508 / S288c) GN=RPL10 PE=1 SV=1	RPL10	25 362,1	100.00%	25	63.80%	221
SDPM Ribosomes	60S ribosomal protein L11-B OS=Saccharomyces cerevisiae (strain ATCC 204508 / S288c) GN=RPL11B PE=1 SV=3	RPL11B	19 750,0	100.00%	8	51.70%	174
SDPM Ribosomes	60S ribosomal protein L12-A OS=Saccharomyces cerevisiae (strain ATCC 204508 / S288c) GN=RPL12A PE=1 SV=1	RPL12A,RPL12B	17 823,2	100.00%	17	71.50%	165
SDPM Ribosomes	60S ribosomal protein L13-B OS=Saccharomyces cerevisiae (strain ATCC 204508 / S288c) GN=RPL13B PE=1 SV=1	RPL13B	22 525,5	100.00%	12	53.30%	199
SDPM Ribosomes	60S ribosomal protein L14-A OS=Saccharomyces cerevisiae (strain ATCC 204508 / S288c) GN=RPL14A PE=1 SV=1	RPL14A	15 167,2	100.00%	9	47.10%	138
SDPM Ribosomes	60S ribosomal protein L14-B OS=Saccharomyces cerevisiae (strain ATCC 204508 / S288c) GN=RPL14B PE=1 SV=1	RPL14B	15 153,2	90.00%	1	41.30%	138
SDPM Ribosomes	60S ribosomal protein L15-A OS=Saccharomyces cerevisiae (strain ATCC 204508 / S288c) GN=RPL15A PE=1 SV=3	RPL15A	24 422,6	100.00%	7	31.40%	204
SDPM Ribosomes	60S ribosomal protein L16-B OS=Saccharomyces cerevisiae (strain ATCC 204508 / S288c) GN=RPL16B PE=1 SV=3	RPL16B	22 249,9	100.00%	9	50.50%	198
SDPM Ribosomes	60S ribosomal protein L16-A OS=Saccharomyces cerevisiae (strain ATCC 204508 / S288c) GN=RPL16A PE=1 SV=3	RPL16A	22 201,6	100.00%	13	46.70%	199
SDPM Ribosomes	60S ribosomal protein L17-A OS=Saccharomyces cerevisiae (strain ATCC 204508 / S288c) GN=RPL17A PE=1 SV=4	RPL17A	20 549,6	100.00%	18	52.20%	184
SDPM Ribosomes	60S ribosomal protein L18-A OS=Saccharomyces cerevisiae (strain ATCC 204508 / S288c) GN=RPL18A PE=1 SV=1	RPL18A,RPL18B	20 563,7	100.00%	11	38.70%	186
SDPM Ribosomes	60S ribosomal protein L19-A OS=Saccharomyces cerevisiae (strain ATCC 204508 / S288c) GN=RPL19A PE=1 SV=1	RPL19A,RPL19B	21 704,6	100.00%	20	41.80%	189
SDPM Ribosomes	60S ribosomal protein L20-A OS=Saccharomyces cerevisiae (strain ATCC 204508 / S288c) GN=RPL20A PE=1 SV=1	RPL20A,RPL20B	20 437,1	100.00%	10	43.00%	172
SDPM Ribosomes	60S ribosomal protein L21-A OS=Saccharomyces cerevisiae (strain ATCC 204508 / S288c) GN=RPL21A PE=1 SV=1	RPL21A	18 242,0	100.00%	7	24.40%	160
SDPM Ribosomes	60S ribosomal protein L22-A OS=Saccharomyces cerevisiae (strain ATCC 204508 / S288c) GN=RPL22A PE=1 SV=3	RPL22A	13 693,4	100.00%	4	58.70%	121
SDPM Ribosomes	60S ribosomal protein L23-A OS=Saccharomyces cerevisiae (strain ATCC 204508 / S288c) GN=RPL23A PE=1 SV=1	RPL23A,RPL23B	14 473,1	100.00%	12	43.10%	137
SDPM Ribosomes	60S ribosomal protein L24-B OS=Saccharomyces cerevisiae (strain ATCC 204508 / S288c) GN=RPL24B PE=1 SV=1	RPL24B	17 548,1	100.00%	10	29.70%	155
SDPM Ribosomes	60S ribosomal protein L25 OS=Saccharomyces cerevisiae (strain ATCC 204508 / S288c) GN=RPL25 PE=1 SV=4	RPL25	15 758,2	100.00%	9	47.20%	142
SDPM Ribosomes	60S ribosomal protein L26-B OS=Saccharomyces cerevisiae (strain ATCC 204508 / S288c) GN=RPL26B PE=1 SV=2	RPL26B	14 235,3	100.00%	13	55.10%	127
SDPM Ribosomes	60S ribosomal protein L26-A OS=Saccharomyces cerevisiae (strain ATCC 204508 / S288c) GN=RPL26A PE=1 SV=3	RPL26A	14 234,3	100.00%	2	55.10%	127
SDPM Ribosomes	60S ribosomal protein L27-A OS=Saccharomyces cerevisiae (strain ATCC 204508 / S288c) GN=RPL27A PE=1 SV=1	RPL27A	15 531,2	100.00%	24	59.60%	136
SDPM Ribosomes	60S ribosomal protein L28 OS=Saccharomyces cerevisiae (strain ATCC 204508 / S288c) GN=RPL28 PE=1 SV=3	RPL28	16 721,9	100.00%	4	35.60%	149
SDPM Ribosomes	60S ribosomal protein L30 OS=Saccharomyces cerevisiae (strain ATCC 204508 / S288c) GN=RPL30 PE=1 SV=3	RPL30	11 416,0	100.00%	15	60.00%	105
SDPM Ribosomes	60S ribosomal protein L31-A OS=Saccharomyces cerevisiae (strain ATCC 204508 / S288c) GN=RPL31A PE=1 SV=1	RPL31A	12 953,2	100.00%	9	55.80%	113
SDPM Ribosomes	60S ribosomal protein L32 OS=Saccharomyces cerevisiae (strain ATCC 204508 / S288c) GN=RPL32 PE=1 SV=1	RPL32	14 771,8	100.00%	13	31.50%	130
SDPM Ribosomes	60S ribosomal protein L33-A OS=Saccharomyces cerevisiae (strain ATCC 204508 / S288c) GN=RPL33A PE=1 SV=3	RPL33A	12 154,2	100.00%	2	25.20%	107
SDPM Ribosomes	60S ribosomal protein L33-B OS=Saccharomyces cerevisiae (strain ATCC 204508 / S288c) GN=RPL33B PE=1 SV=2	RPL33B	12 168,2	100.00%	1	25.20%	107
SDPM Ribosomes	60S ribosomal protein L34-A OS=Saccharomyces cerevisiae (strain ATCC 204508 / S288c) GN=RPL34A PE=1 SV=1	RPL34A,RPL34B	13 639,2	100.00%	3	20.70%	121
SDPM Ribosomes	60S ribosomal protein L35-A OS=Saccharomyces cerevisiae (strain ATCC 204508 / S288c) GN=RPL35A PE=1 SV=1	RPL35A,RPL35B	13 910,2	100.00%	10	45.00%	120
SDPM Ribosomes	60S ribosomal protein L36-B OS=Saccharomyces cerevisiae (strain ATCC 204508 / S288c) GN=RPL36B PE=1 SV=3	RPL36B	11 135,9	100.00%	1	23.00%	100
SDPM Ribosomes	60S ribosomal protein L36-A OS=Saccharomyces cerevisiae (strain ATCC 204508 / S288c) GN=RPL36A PE=1 SV=3	RPL36A	11 124,9	100.00%	3	14.00%	100
SDPM Ribosomes	60S ribosomal protein L37-A OS=Saccharomyces cerevisiae (strain ATCC 204508 / S288c) GN=RPL37A PE=1 SV=2	RPL37A	9 850,4	100.00%	5	38.60%	88
SDPM Ribosomes	60S ribosomal protein L37-B OS=Saccharomyces cerevisiae (strain ATCC 204508 / S288c) GN=RPL37B PE=1 SV=2	RPL37B	9 868,3	48.00%	0	11.40%	88
SDPM Ribosomes	60S ribosomal protein L38 OS=Saccharomyces cerevisiae (strain ATCC 204508 / S288c) GN=RPL38 PE=1 SV=1	RPL38	8 827,2	100.00%	8	62.80%	78

SDPM Ribosomes	60S ribosomal protein L39 OS=Saccharomyces cerevisiae (strain ATCC 204508 / S288c) GN=RPL39 PE=1 SV=3	RPL39	6 342,0	100.00%	4	21.60%	51
SDPM Ribosomes	Ubiquitin-60S ribosomal protein L40 OS=Saccharomyces cerevisiae (strain ATCC 204508 / S288c) GN=RPL40A PE=1 SV=1	RPL40A,RPL40B	14 554,8	99.50%	1	15.60%	128
SDPM Ribosomes	60S ribosomal protein L42-A OS=Saccharomyces cerevisiae (strain ATCC 204508 / S288c) GN=RPL42A PE=1 SV=1	RPL42A,RPL42B	12 211,5	100.00%	6	42.50%	106
SDPM Ribosomes	60S ribosomal protein L43-A OS=Saccharomyces cerevisiae (strain ATCC 204508 / S288c) GN=RPL43A PE=1 SV=1	RPL43A,RPL43B	10 090,8	100.00%	7	54.30%	92
SDPM Ribosomes	60S acidic ribosomal protein P1-alpha OS=Saccharomyces cerevisiae (strain ATCC 204508 / S288c) GN=RPP1A PE=1 SV=4	RPP1A	10 908,3	100.00%	3	91.50%	106
SDPM Ribosomes	60S acidic ribosomal protein P2-beta OS=Saccharomyces cerevisiae (strain ATCC 204508 / S288c) GN=RPP2B PE=1 SV=2	RPP2B	11 050,3	100.00%	8	90.00%	110
SDPM Ribosomes	60S acidic ribosomal protein P2-alpha OS=Saccharomyces cerevisiae (strain ATCC 204508 / S288c) GN=RPP2A PE=1 SV=1	RPP2A	10 746,2	100.00%	8	88.70%	106
SDPM Ribosomes	60S acidic ribosomal protein P1-beta OS=Saccharomyces cerevisiae (strain ATCC 204508 / S288c) GN=RPP1B PE=1 SV=3	RPP1B	10 668,1	100.00%	7	67.00%	106
SDPM Ribosomes	60S acidic ribosomal protein P0 OS=Saccharomyces cerevisiae (strain ATCC 204508 / S288c) GN=RPP0 PE=1 SV=2	RPP0	33 717,4	100.00%	10	46.80%	312
SDPM Ribosomes	40S ribosomal protein S0-B OS=Saccharomyces cerevisiae (strain RM11-1a) GN=RPS0B PE=3 SV=1	RPS0B	27 962,3	100.00%	18	79.00%	252
SDPM Ribosomes	40S ribosomal protein S1-B OS=Saccharomyces cerevisiae (strain RM11-1a) GN=RPS1B PE=3 SV=1	RPS1B	28 812,9	100.00%	27	65.50%	255
SDPM Ribosomes	40S ribosomal protein S1-A OS=Saccharomyces cerevisiae (strain RM11-1a) GN=RPS1A PE=3 SV=1	RPS1A	28 743,8	100.00%	3	62.00%	255
SDPM Ribosomes	40S ribosomal protein S2 OS=Saccharomyces cerevisiae (strain ATCC 204508 / S288c) GN=RPS2 PE=1 SV=3	RPS2	27 450,2	100.00%	21	68.90%	254
SDPM Ribosomes	40S ribosomal protein S3 OS=Saccharomyces cerevisiae (strain ATCC 204508 / S288c) GN=RPS3 PE=1 SV=5	RPS3	26 503,0	100.00%	21	60.80%	240
SDPM Ribosomes	40S ribosomal protein S4-A OS=Saccharomyces cerevisiae (strain ATCC 204508 / S288c) GN=RPS4A PE=1 SV=1	RPS4A,RPS4B	29 411,2	100.00%	34	65.50%	261
SDPM Ribosomes	40S ribosomal protein S5 OS=Saccharomyces cerevisiae (strain ATCC 204508 / S288c) GN=RPS5 PE=1 SV=3	RPS5	25 038,5	100.00%	33	78.70%	225
SDPM Ribosomes	40S ribosomal protein S6-A OS=Saccharomyces cerevisiae (strain ATCC 204508 / S288c) GN=RPS6A PE=1 SV=1	RPS6A,RPS6B	26 997,2	100.00%	28	63.10%	236
SDPM Ribosomes	40S ribosomal protein S7-B OS=Saccharomyces cerevisiae (strain ATCC 204508 / S288c) GN=RPS7B PE=1 SV=1	RPS7B	21 634,7	100.00%	21	63.70%	190
SDPM Ribosomes	40S ribosomal protein S7-A OS=Saccharomyces cerevisiae (strain ATCC 204508 / S288c) GN=RPS7A PE=1 SV=4	RPS7A	21 622,7	100.00%	10	63.70%	190
SDPM Ribosomes	40S ribosomal protein S8-A OS=Saccharomyces cerevisiae (strain ATCC 204508 / S288c) GN=RPS8A PE=1 SV=1	RPS8A,RPS8B	22 490,4	100.00%	14	55.50%	200
SDPM Ribosomes	40S ribosomal protein S9-B OS=Saccharomyces cerevisiae (strain ATCC 204508 / S288c) GN=RPS9B PE=1 SV=4	RPS9B	22 299,7	100.00%	17	50.80%	195
SDPM Ribosomes	40S ribosomal protein S10-B OS=Saccharomyces cerevisiae (strain ATCC 204508 / S288c) GN=RPS10B PE=1 SV=1	RPS10B	12 738,7	100.00%	11	62.90%	105
SDPM Ribosomes	40S ribosomal protein S11-A OS=Saccharomyces cerevisiae (strain ATCC 204508 / S288c) GN=RPS11A PE=1 SV=1	RPS11A,RPS11B	17 748,8	100.00%	17	56.40%	156
SDPM Ribosomes	40S ribosomal protein S12 OS=Saccharomyces cerevisiae (strain ATCC 204508 / S288c) GN=RPS12 PE=1 SV=1	RPS12	15 470,9	100.00%	10	65.00%	143
SDPM Ribosomes	40S ribosomal protein S13 OS=Saccharomyces cerevisiae (strain ATCC 204508 / S288c) GN=RPS13 PE=1 SV=3	RPS13	17 029,8	100.00%	10	50.30%	151
SDPM Ribosomes	40S ribosomal protein S14-B OS=Saccharomyces cerevisiae (strain ATCC 204508 / S288c) GN=RPS14B PE=1 SV=2	RPS14B	14 649,8	100.00%	14	63.80%	138
SDPM Ribosomes	40S ribosomal protein S15 OS=Saccharomyces cerevisiae (strain ATCC 204508 / S288c) GN=RPS15 PE=1 SV=1	RPS15	16 002,2	100.00%	12	57.70%	142
SDPM Ribosomes	40S ribosomal protein S16-A OS=Saccharomyces cerevisiae (strain ATCC 204508 / S288c) GN=RPS16A PE=1 SV=1	RPS16A,RPS16B	15 847,9	100.00%	14	62.90%	143
SDPM Ribosomes	40S ribosomal protein S17-A OS=Saccharomyces cerevisiae (strain ATCC 204508 / S288c) GN=RPS17A PE=1 SV=1	RPS17A,RPS17B	15 788,9	100.00%	14	50.70%	136
SDPM Ribosomes	40S ribosomal protein S18-A OS=Saccharomyces cerevisiae (strain ATCC 204508 / S288c) GN=RPS18A PE=1 SV=1	RPS18A,RPS18B	17 037,9	100.00%	21	69.20%	146
SDPM Ribosomes	40S ribosomal protein S19-A OS=Saccharomyces cerevisiae (strain ATCC 204508 / S288c) GN=RPS19A PE=1 SV=2	RPS19A	15 917,4	100.00%	15	83.30%	144
SDPM Ribosomes	40S ribosomal protein S20 OS=Saccharomyces cerevisiae (strain ATCC 204508 / S288c) GN=RPS20 PE=1 SV=3	RPS20	13 906,8	100.00%	9	54.50%	121
SDPM Ribosomes	40S ribosomal protein S21-B OS=Saccharomyces cerevisiae (strain ATCC 204508 / S288c) GN=RPS21B PE=1 SV=1	RPS21B	9 760,0	100.00%	6	72.40%	87
SDPM Ribosomes	40S ribosomal protein S22-A OS=Saccharomyces cerevisiae (strain ATCC 204508 / S288c) GN=RPS22A PE=1 SV=2	RPS22A,RPS22B	14 626,5	100.00%	19	82.30%	130
SDPM Ribosomes	40S ribosomal protein S23-A OS=Naumovozyma castellii (strain ATCC 76901 / CBS 4309 / NBRC 1992 / NRRL Y-12630) GN=	RPS23,RPS23A,RPS2	16 038,3	100.00%	13	39.30%	145
SDPM Ribosomes	40S ribosomal protein S24-A OS=Saccharomyces cerevisiae (strain ATCC 204508 / S288c) GN=RPS24A PE=1 SV=1	RPS24A,RPS24B	15 329,0	100.00%	12	44.40%	135
SDPM Ribosomes	40S ribosomal protein S25-A OS=Saccharomyces cerevisiae (strain ATCC 204508 / S288c) GN=RPS25A PE=1 SV=1	RPS25A,RPS25B	12 009,9	100.00%	13	57.40%	108
SDPM Ribosomes	40S ribosomal protein S26-A OS=Saccharomyces cerevisiae (strain ATCC 204508 / S288c) GN=RPS26A PE=1 SV=1	RPS26A,RPS26B	13 505,0	100.00%	5	33.60%	119
SDPM Ribosomes	40S ribosomal protein S27-B OS=Saccharomyces cerevisiae (strain ATCC 204508 / S288c) GN=RPS27B PE=1 SV=1	RPS27B	8 865,2	100.00%	4	62.20%	82
SDPM Ribosomes	40S ribosomal protein S28-A OS=Saccharomyces cerevisiae (strain ATCC 204508 / S288c) GN=RPS28A PE=1 SV=1	RPS28A,RPS28B	7 591,7	100.00%	8	53.70%	67
SDPM Ribosomes	40S ribosomal protein S29-B OS=Saccharomyces cerevisiae (strain ATCC 204508 / S288c) GN=RPS29B PE=1 SV=3	RPS29B	6 727,6	100.00%	3	69.60%	56
SDPM Ribosomes	40S ribosomal protein S29-A OS=Saccharomyces cerevisiae (strain ATCC 204508 / S288c) GN=RPS29A PE=1 SV=3	RPS29A	6 660,7	100.00%	3	51.80%	56
SDPM Ribosomes	40S ribosomal protein S30-A OS=Saccharomyces cerevisiae (strain ATCC 204508 / S288c) GN=RPS30A PE=1 SV=1	RPS30A,RPS30B	7 118,5	100.00%	3	36.50%	63
SDPM Ribosomes	Ubiquitin-40S ribosomal protein S31 OS=Saccharomyces cerevisiae (strain ATCC 204508 / S288c) GN=RPS31 PE=1 SV=3	RPS31	17 216,6	100.00%	11	38.20%	152
SDPM Ribosomes	Guanine nucleotide-binding protein subunit beta-like protein OS=Saccharomyces cerevisiae (strain ATCC 204508 / S288c) GN=A	ASC1	34 805,9	100.00%	30	79.00%	319

Mass spectrometry analysis was performed on purified ribosomal samples (20 pmole of WT and SDPM ribosomes). Samples were subjected to trypsin digestion and peptides were further identified by tandem mass spectrometry (MS/MS). Peptide and protein identifications were performed using the MASCOT search engine against a uniprot-yeast database. Database searches were performed using trypsin cleavage specificity with two possible missed cleavages. Carbamidomethylation of cysteines was set as fixed modification and oxidation of methionines as variable modifications. Peptide and fragment tolerances were set at 10 ppm and 0.6 Da respectively. Only ions with a score higher than the identity threshold and a false-positive discovery rate (FDR) of less than 1% (Mascot decoy option) were considered.
