# Supplementary figures and images for: Phosphorylation of P-stalk proteins defines the ribosomal state for interaction with auxiliary protein factors
Source: EMBO Rep. 2024 Oct 28;25(12):5478–506. doi: 10.1038/s44319-024-00297-1 (PMC11624264; doi:10.1038/s44319-024-00297-1)

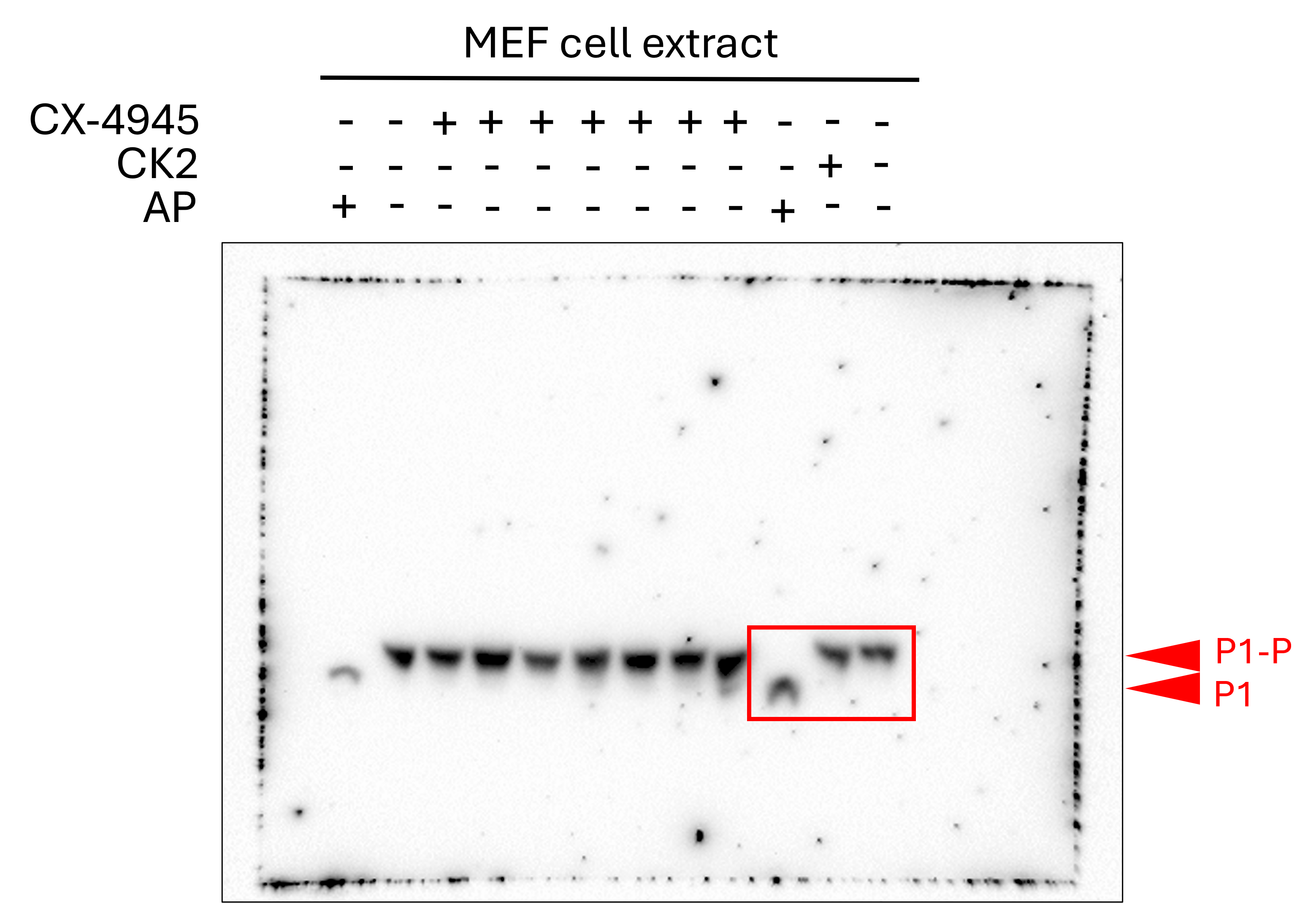

Supplement: Supplementary file 3 — Source data Fig. 2 [file 44319_2024_297_MOESM3_ESM.zip › Figure 2/Fig2A - Western blot MEF cell extract P1.tif]

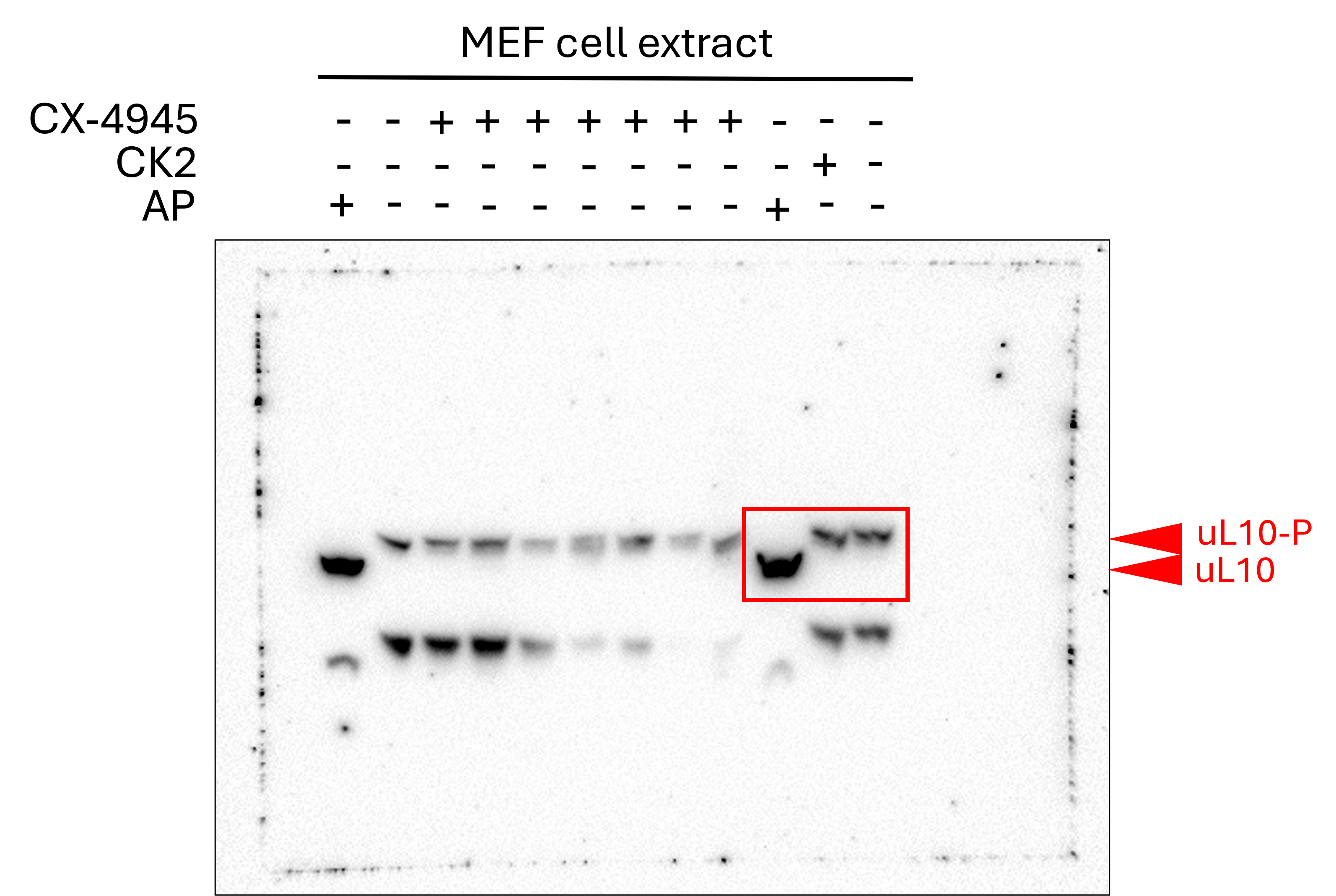

Supplement: Supplementary file 3 — Source data Fig. 2 [file 44319_2024_297_MOESM3_ESM.zip › Figure 2/Fig2A - Western blot MEF cell extract uL10.tif]

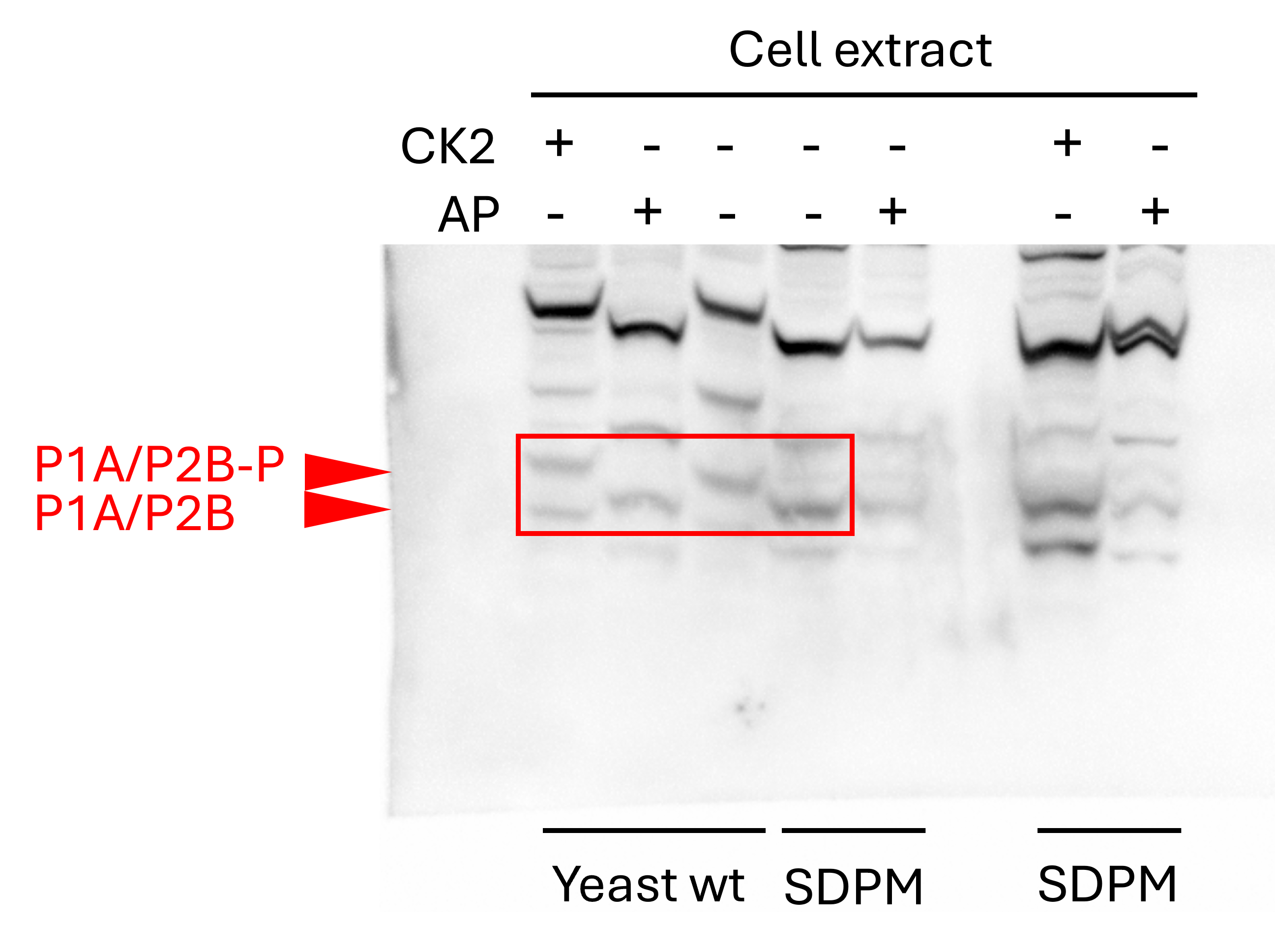

Supplement: Supplementary file 3 — Source data Fig. 2 [file 44319_2024_297_MOESM3_ESM.zip › Figure 2/Fig2A - Western blot yeast extract P1AP2B.tif]

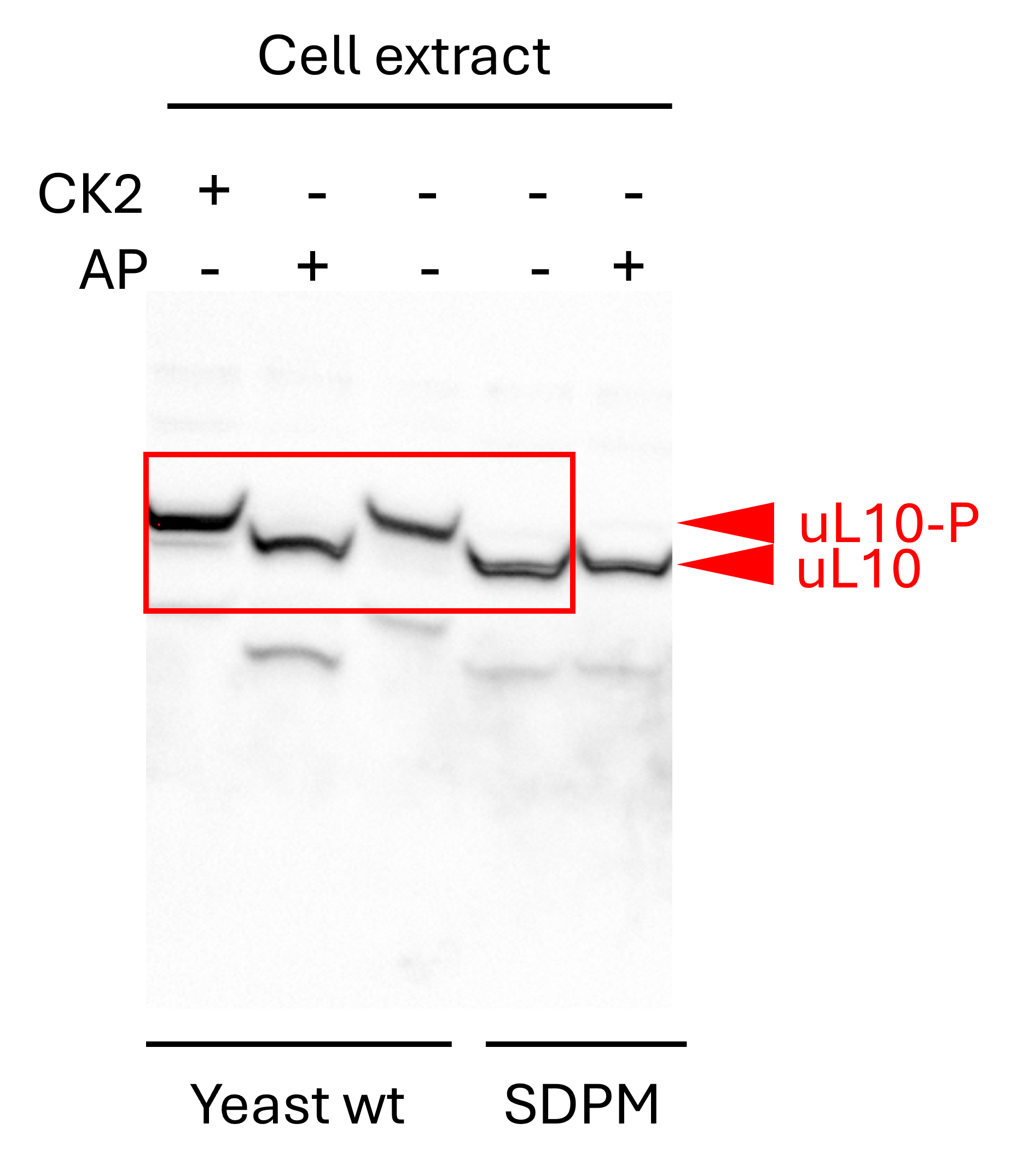

Supplement: Supplementary file 3 — Source data Fig. 2 [file 44319_2024_297_MOESM3_ESM.zip › Figure 2/Fig2A - Western blot yeast extract uL10.tif]

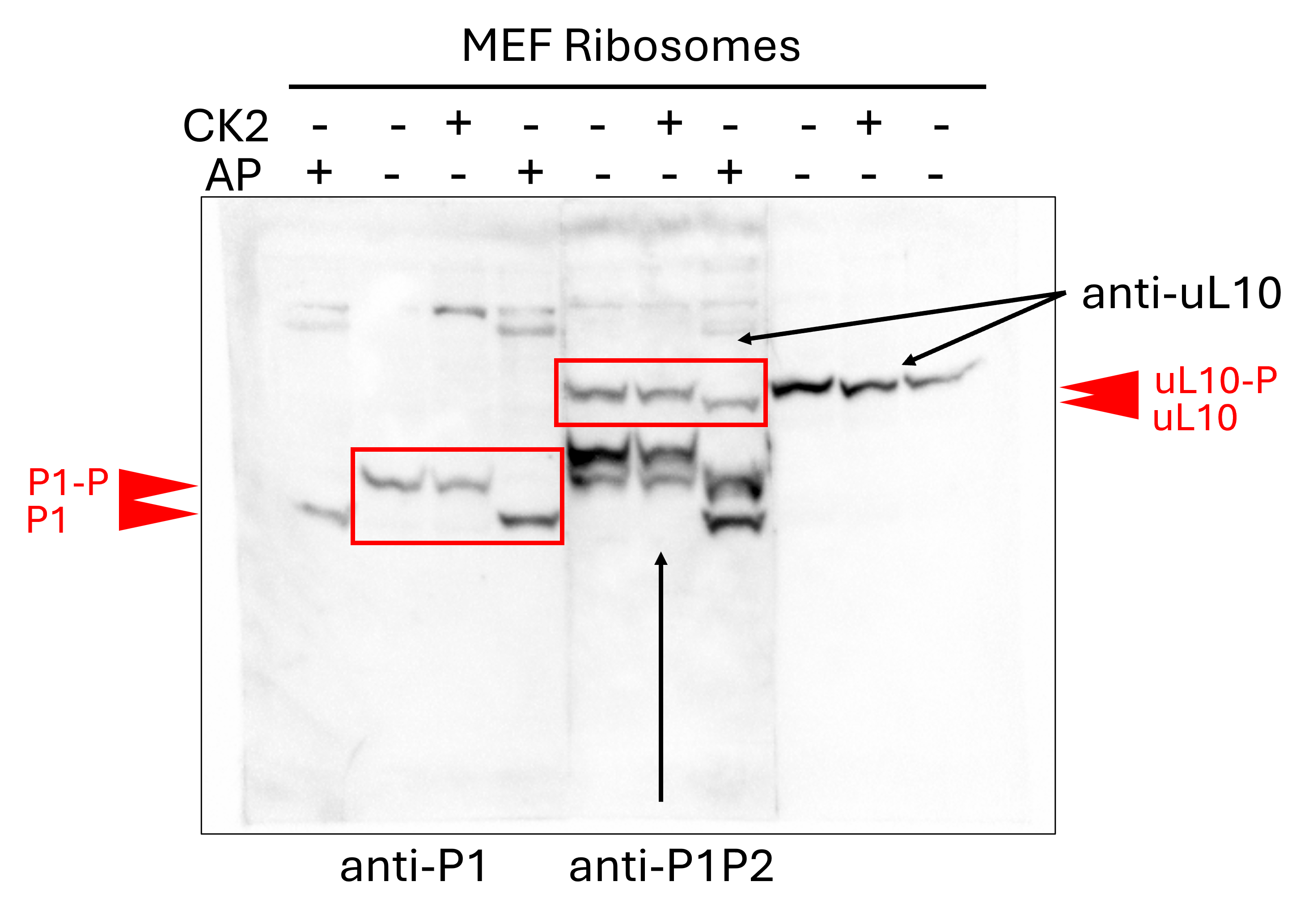

Supplement: Supplementary file 3 — Source data Fig. 2 [file 44319_2024_297_MOESM3_ESM.zip › Figure 2/Fig2B - Western blot MEF ribosome.tif]

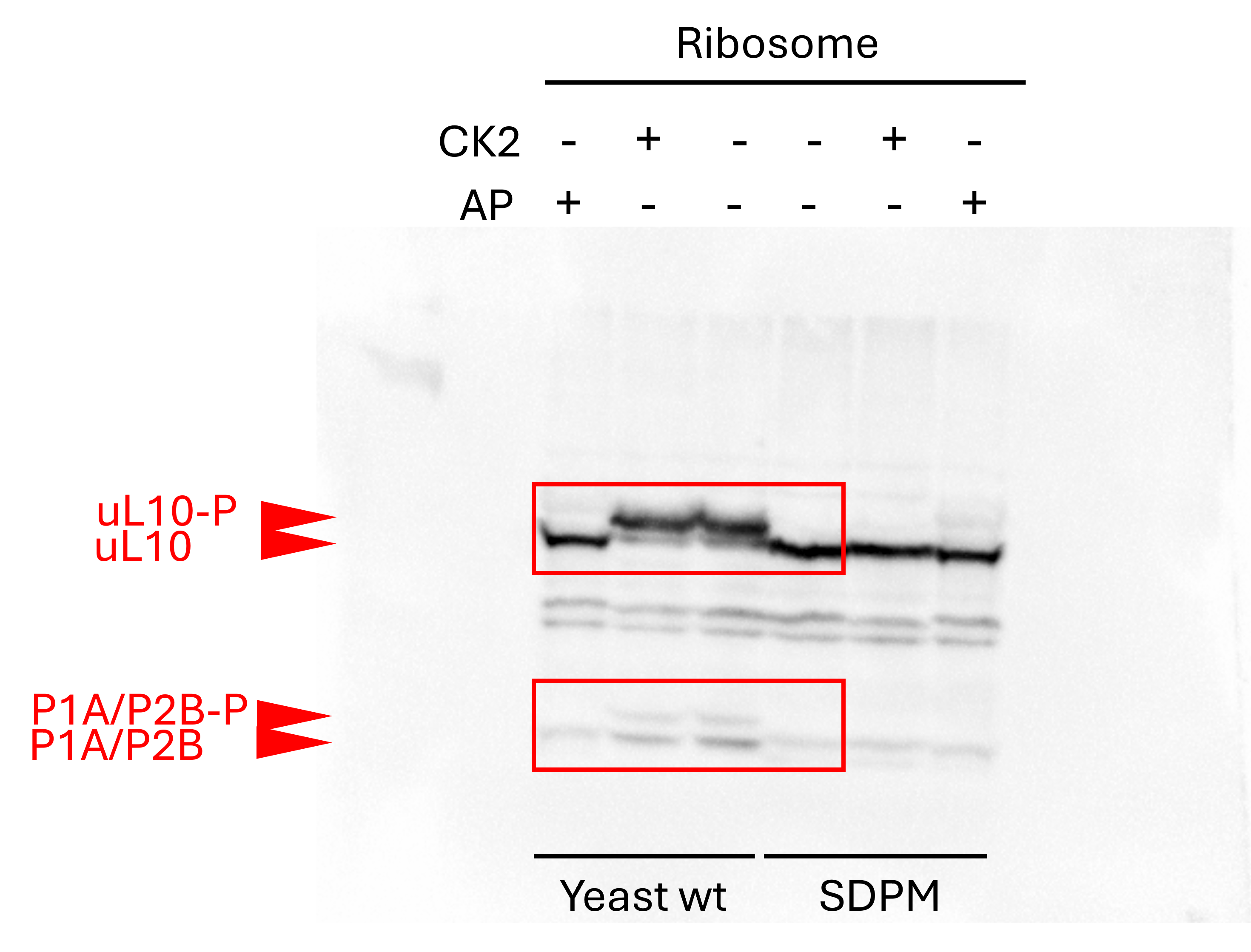

Supplement: Supplementary file 3 — Source data Fig. 2 [file 44319_2024_297_MOESM3_ESM.zip › Figure 2/Fig2B - Western blot yeast ribosome.tif]

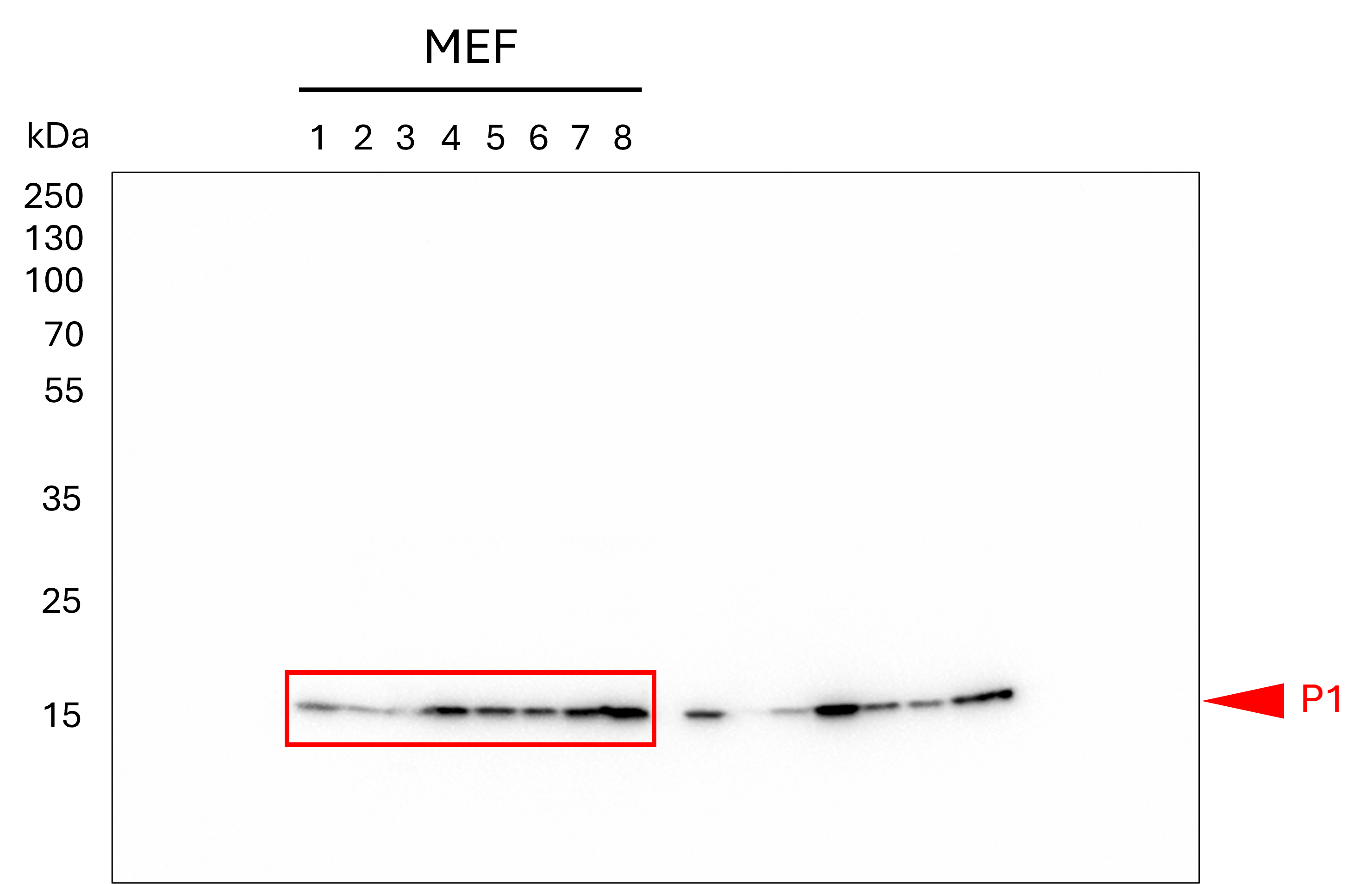

Supplement: Supplementary file 4 — Source data Fig. 3 [file 44319_2024_297_MOESM4_ESM.zip › Figure 3/Fig3A - Western blot P1 MEF.tif]

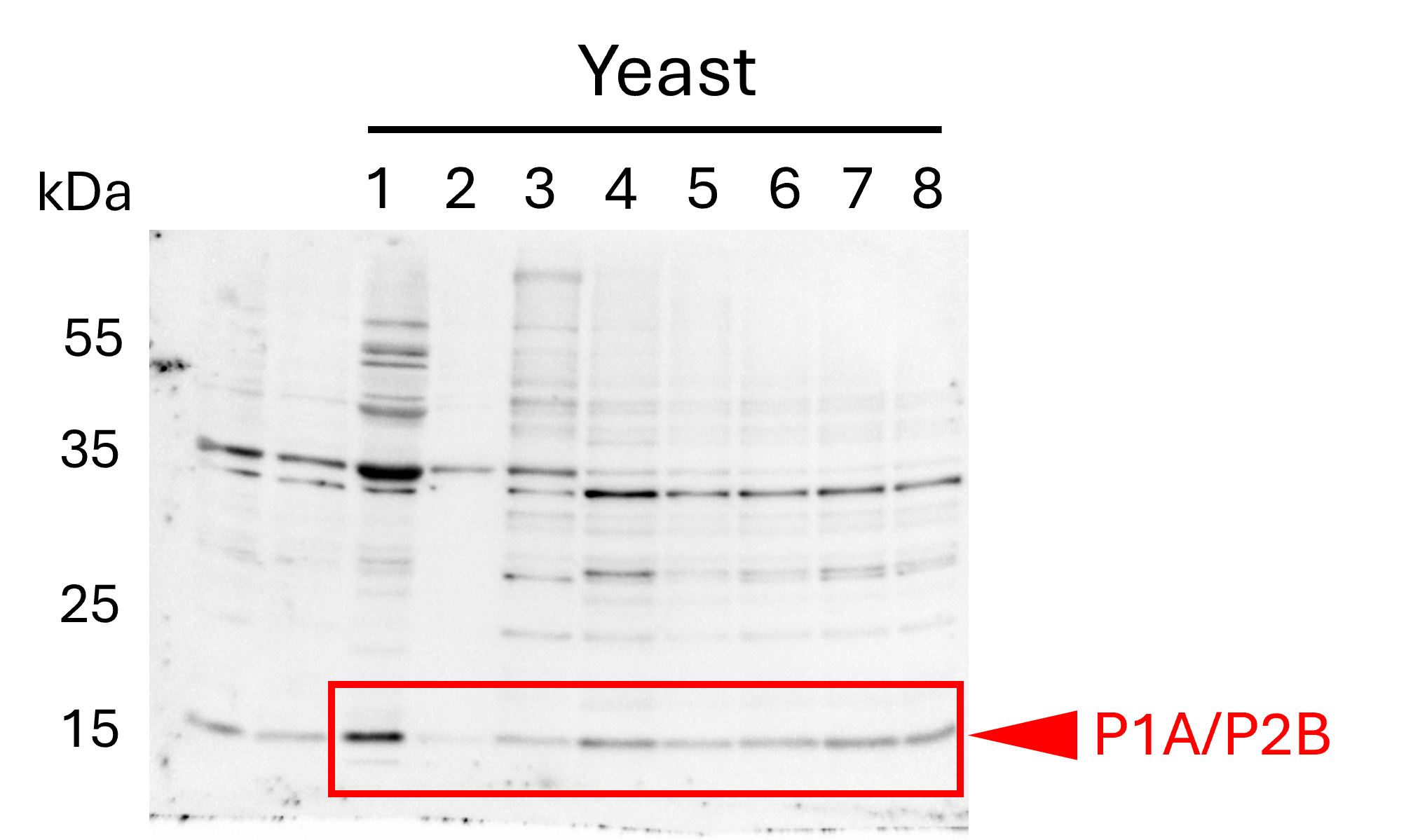

Supplement: Supplementary file 4 — Source data Fig. 3 [file 44319_2024_297_MOESM4_ESM.zip › Figure 3/Fig3A - Western blot P1AP2B Yeast.tif]

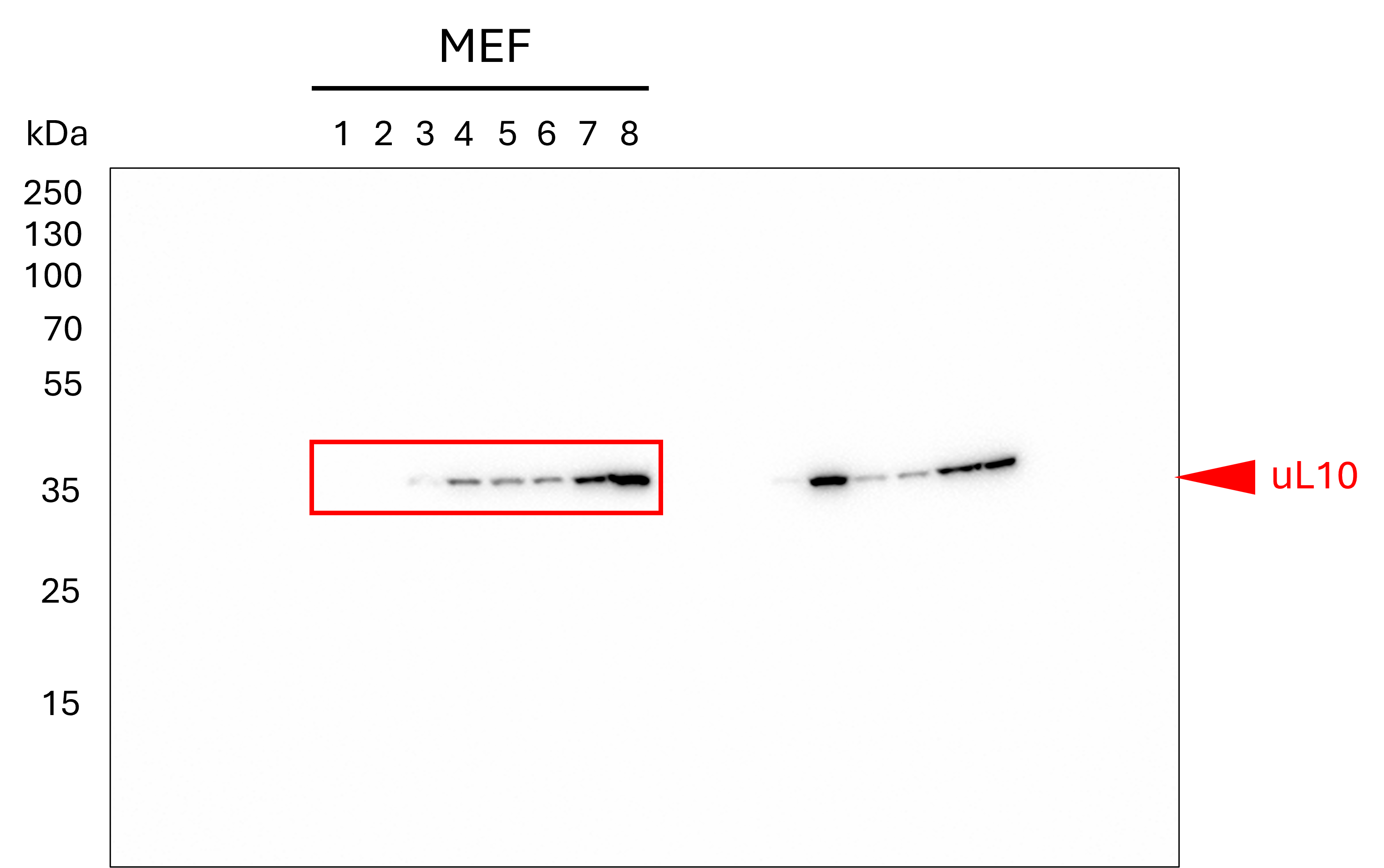

Supplement: Supplementary file 4 — Source data Fig. 3 [file 44319_2024_297_MOESM4_ESM.zip › Figure 3/Fig3A - Western blot uL10 MEF.tif]

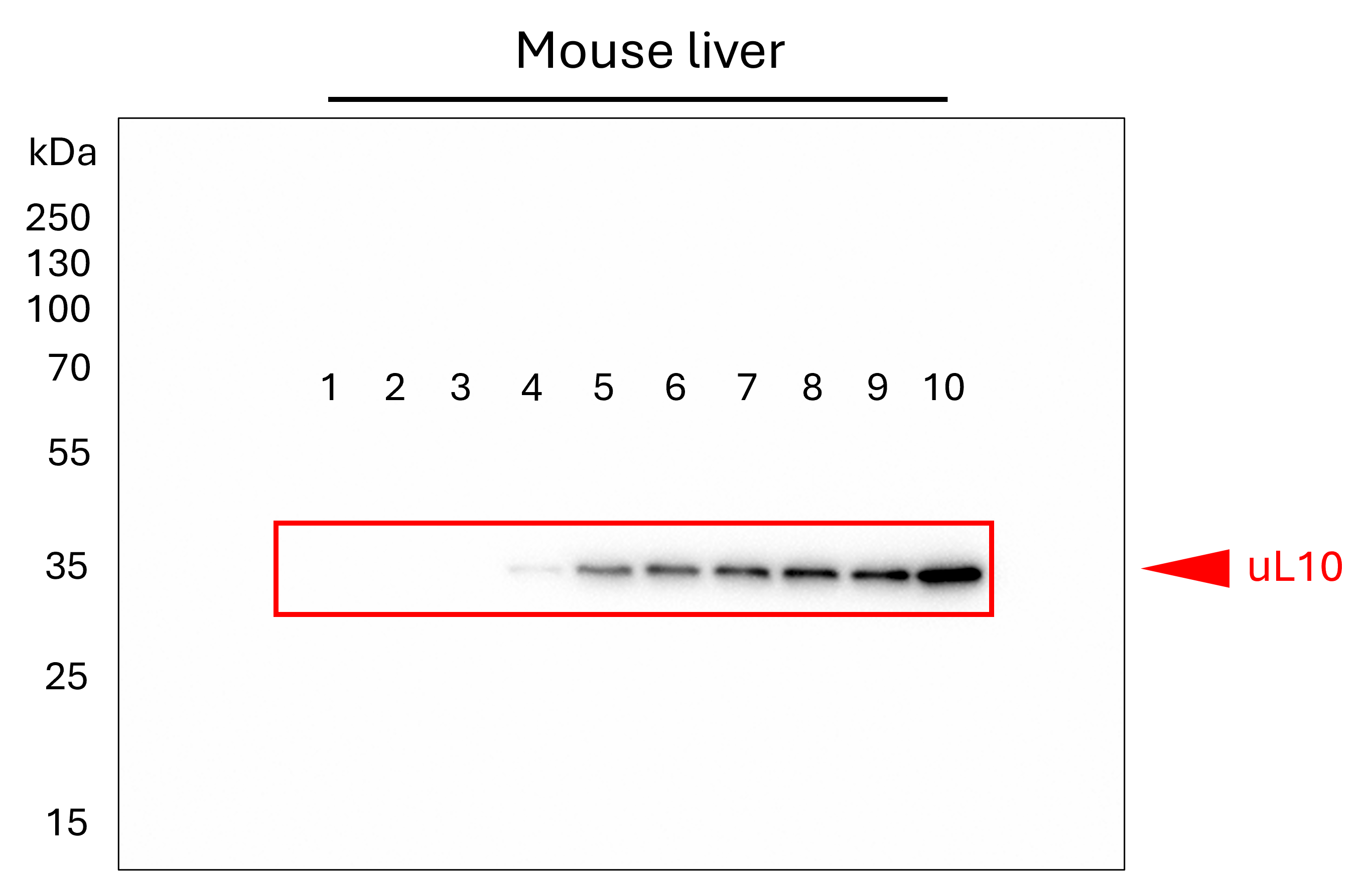

Supplement: Supplementary file 4 — Source data Fig. 3 [file 44319_2024_297_MOESM4_ESM.zip › Figure 3/Fig3A - Western blot uL10 mouse liver.tif]

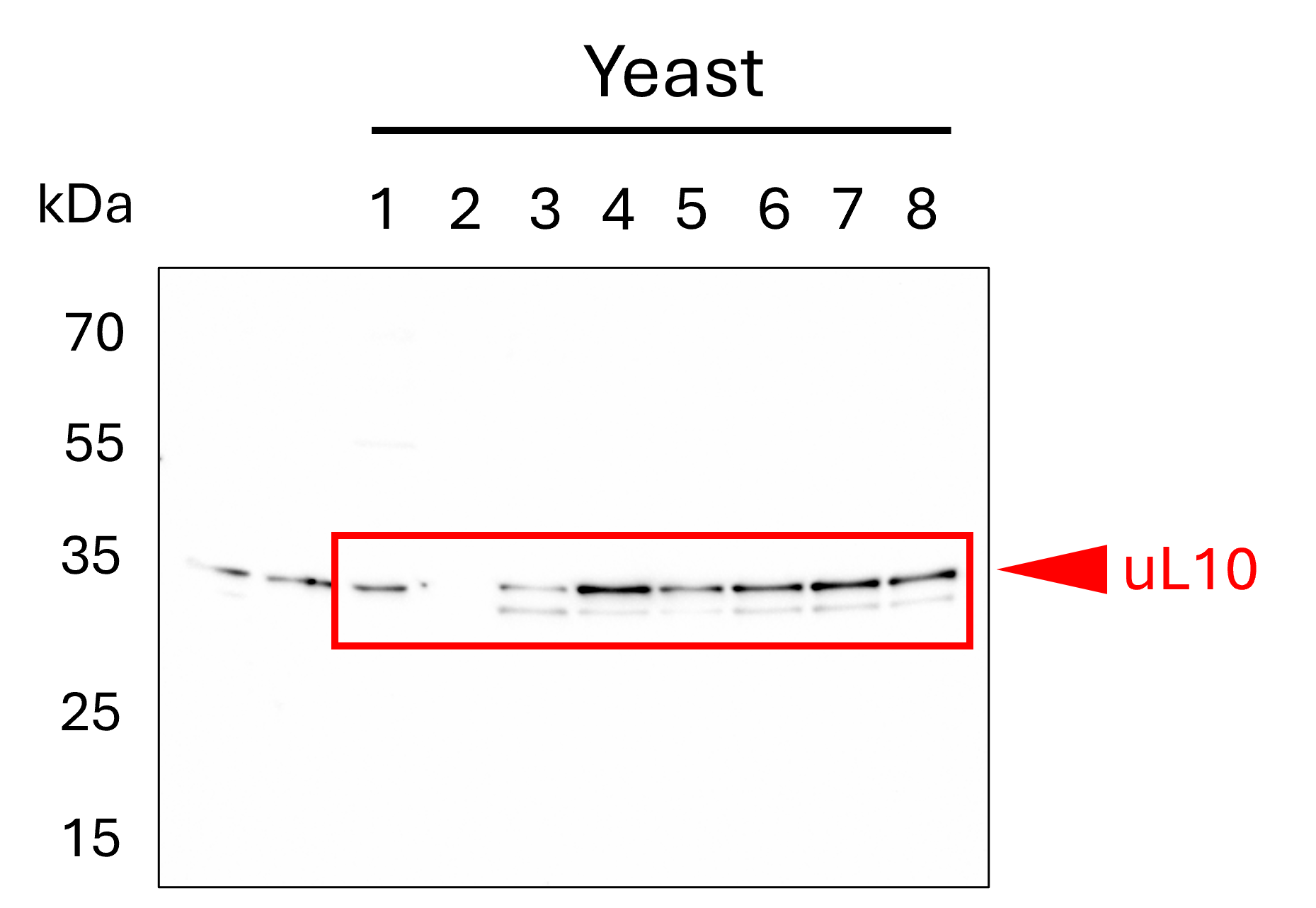

Supplement: Supplementary file 4 — Source data Fig. 3 [file 44319_2024_297_MOESM4_ESM.zip › Figure 3/Fig3A - Western blot uL10 Yeast.tif]

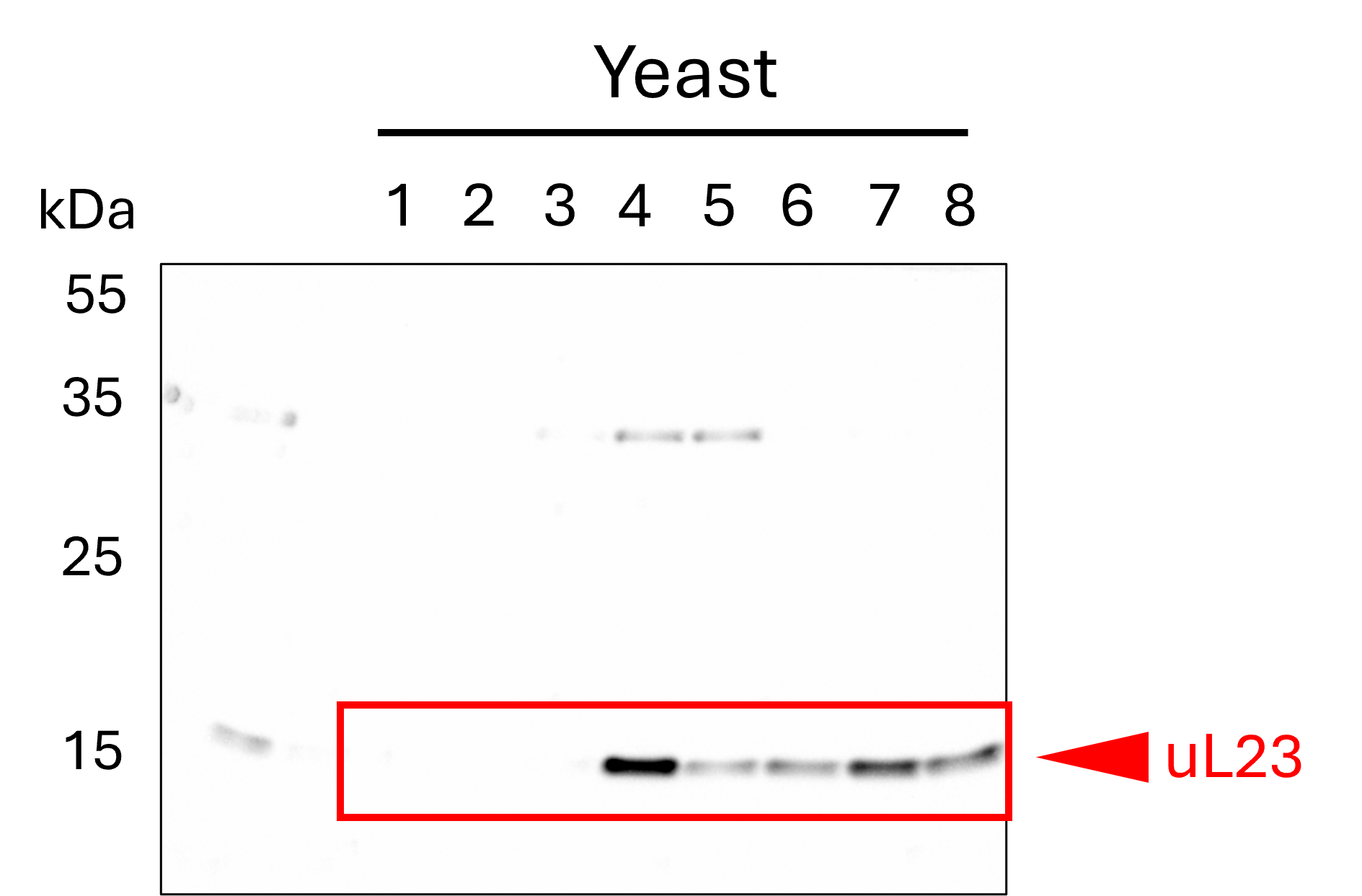

Supplement: Supplementary file 4 — Source data Fig. 3 [file 44319_2024_297_MOESM4_ESM.zip › Figure 3/Fig3A - Western blot uL23 Yeast.tif]

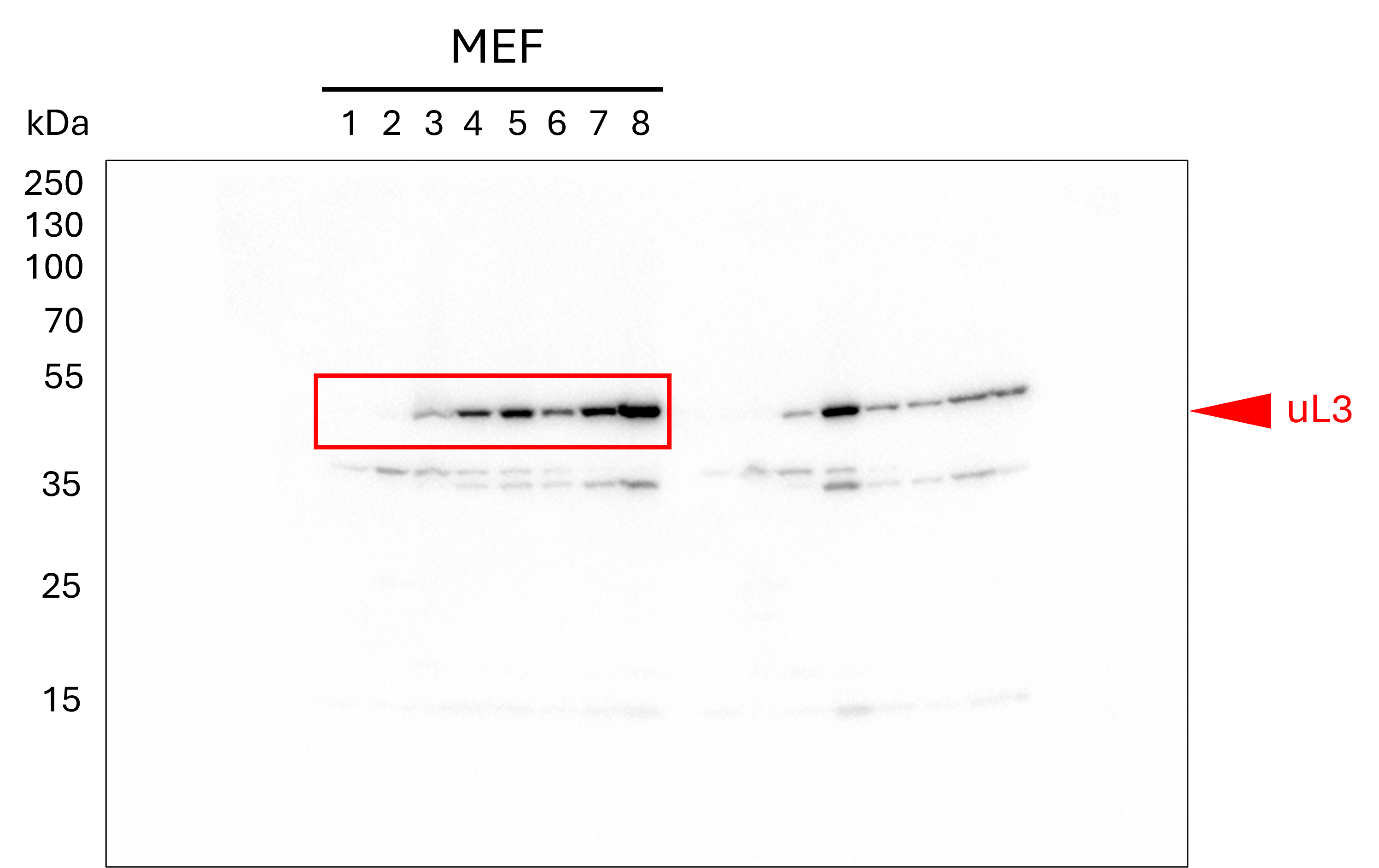

Supplement: Supplementary file 4 — Source data Fig. 3 [file 44319_2024_297_MOESM4_ESM.zip › Figure 3/Fig3A - Western blot uL3 MEF.tif]

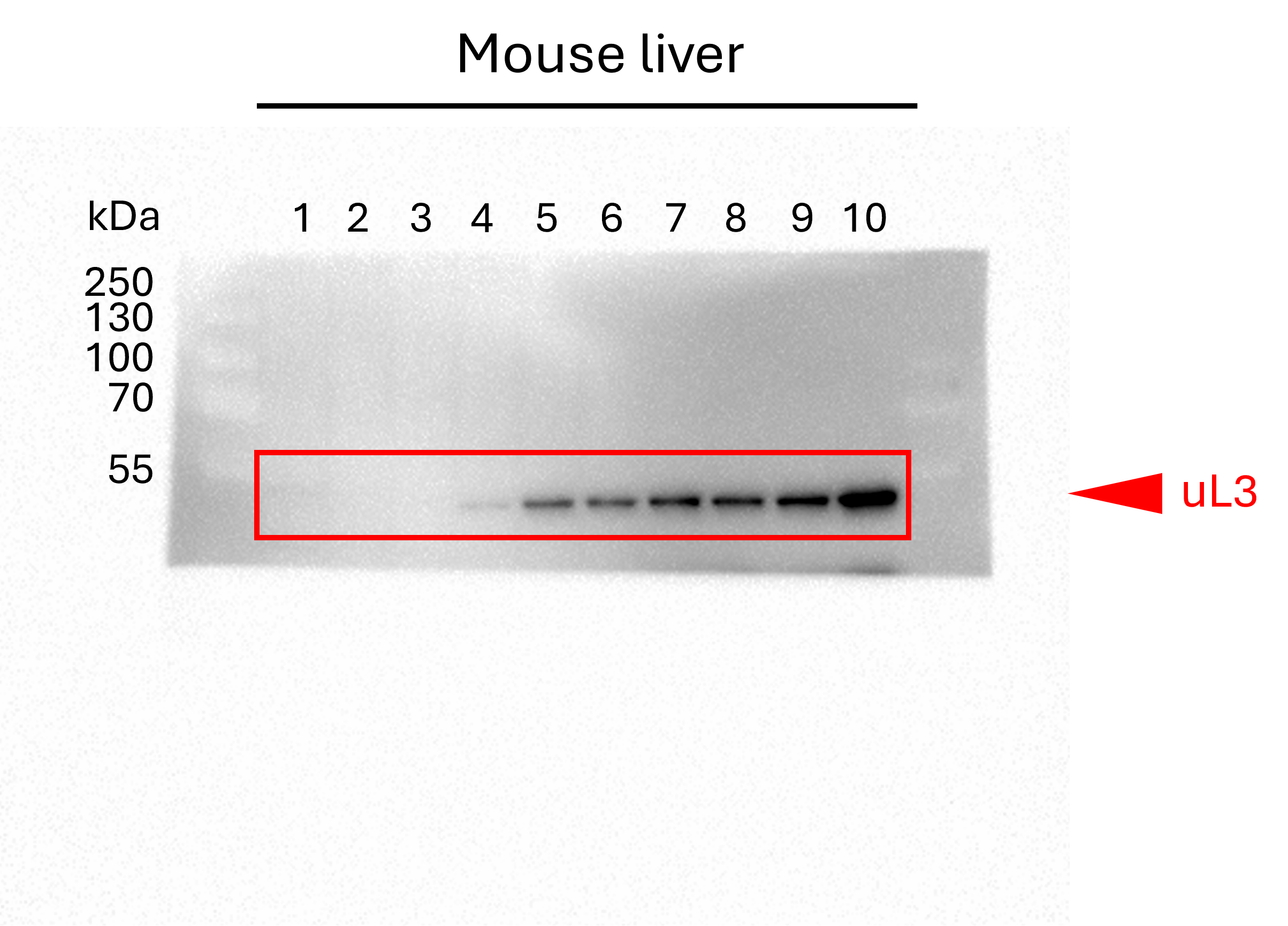

Supplement: Supplementary file 4 — Source data Fig. 3 [file 44319_2024_297_MOESM4_ESM.zip › Figure 3/Fig3A - Western blot uL3 mouse liver.tif]

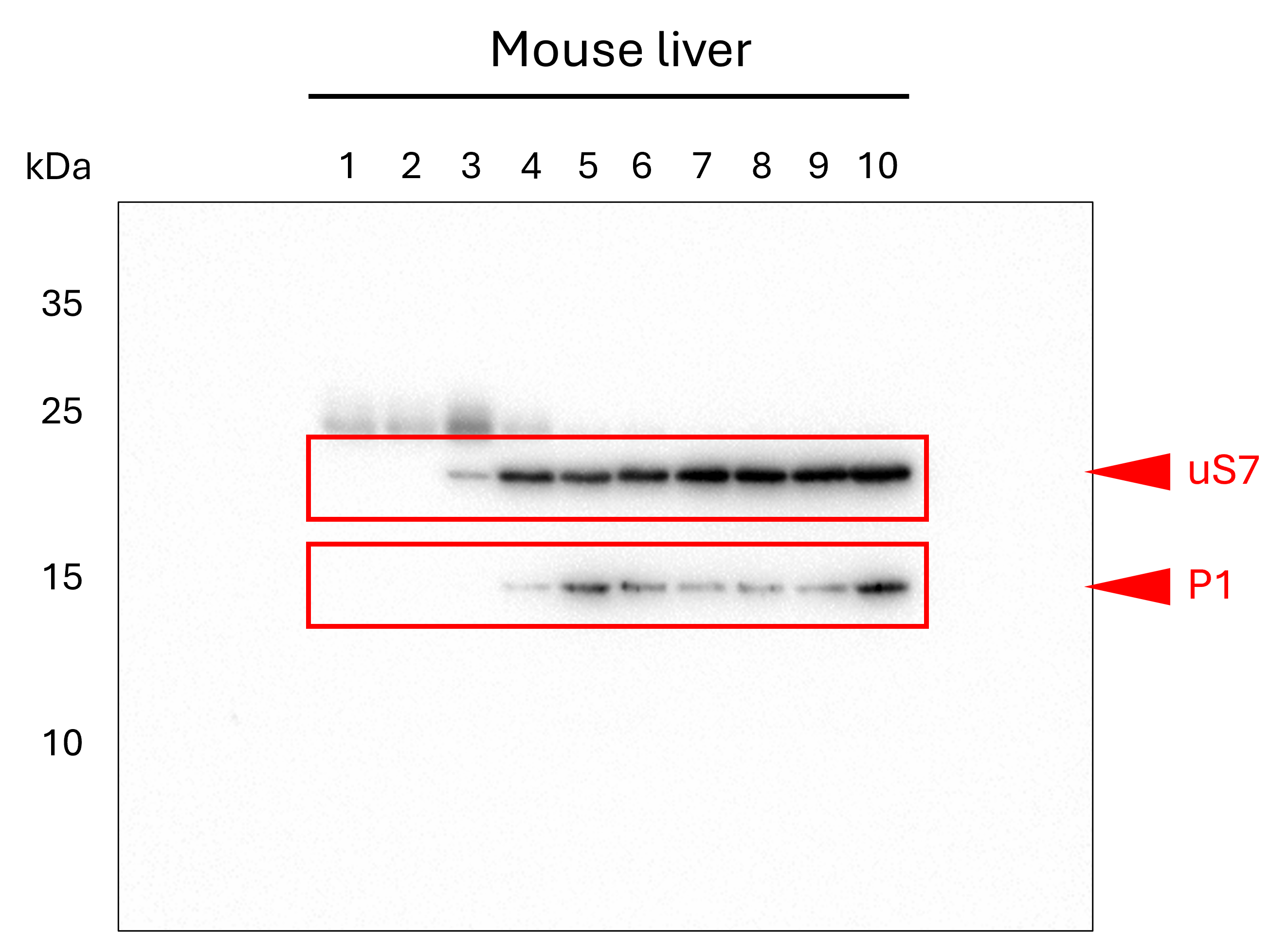

Supplement: Supplementary file 4 — Source data Fig. 3 [file 44319_2024_297_MOESM4_ESM.zip › Figure 3/Fig3A - Western blot uS7 and P1 mouse liver.tif]

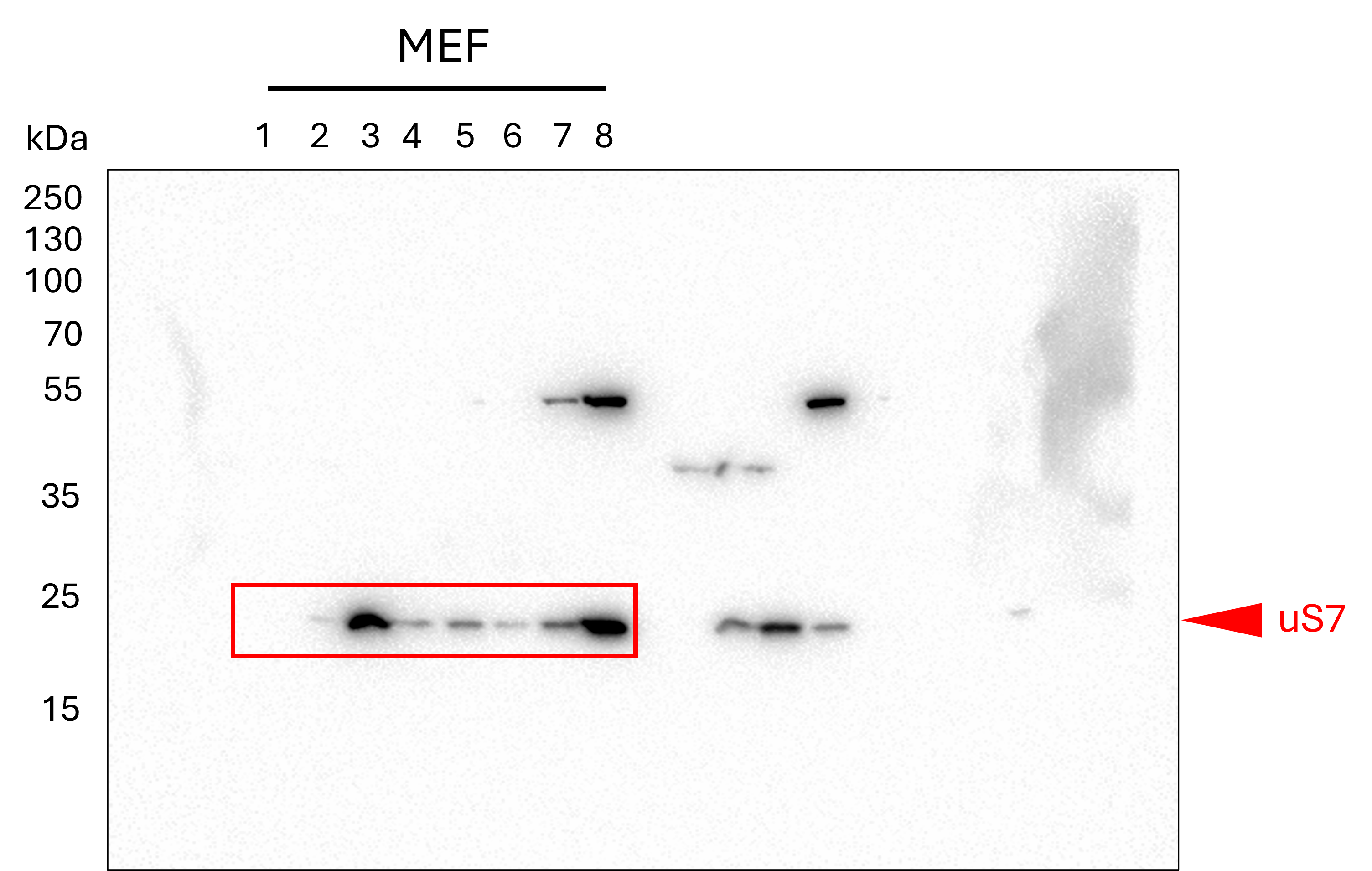

Supplement: Supplementary file 4 — Source data Fig. 3 [file 44319_2024_297_MOESM4_ESM.zip › Figure 3/Fig3A - Western blot uS7 MEF.tif]

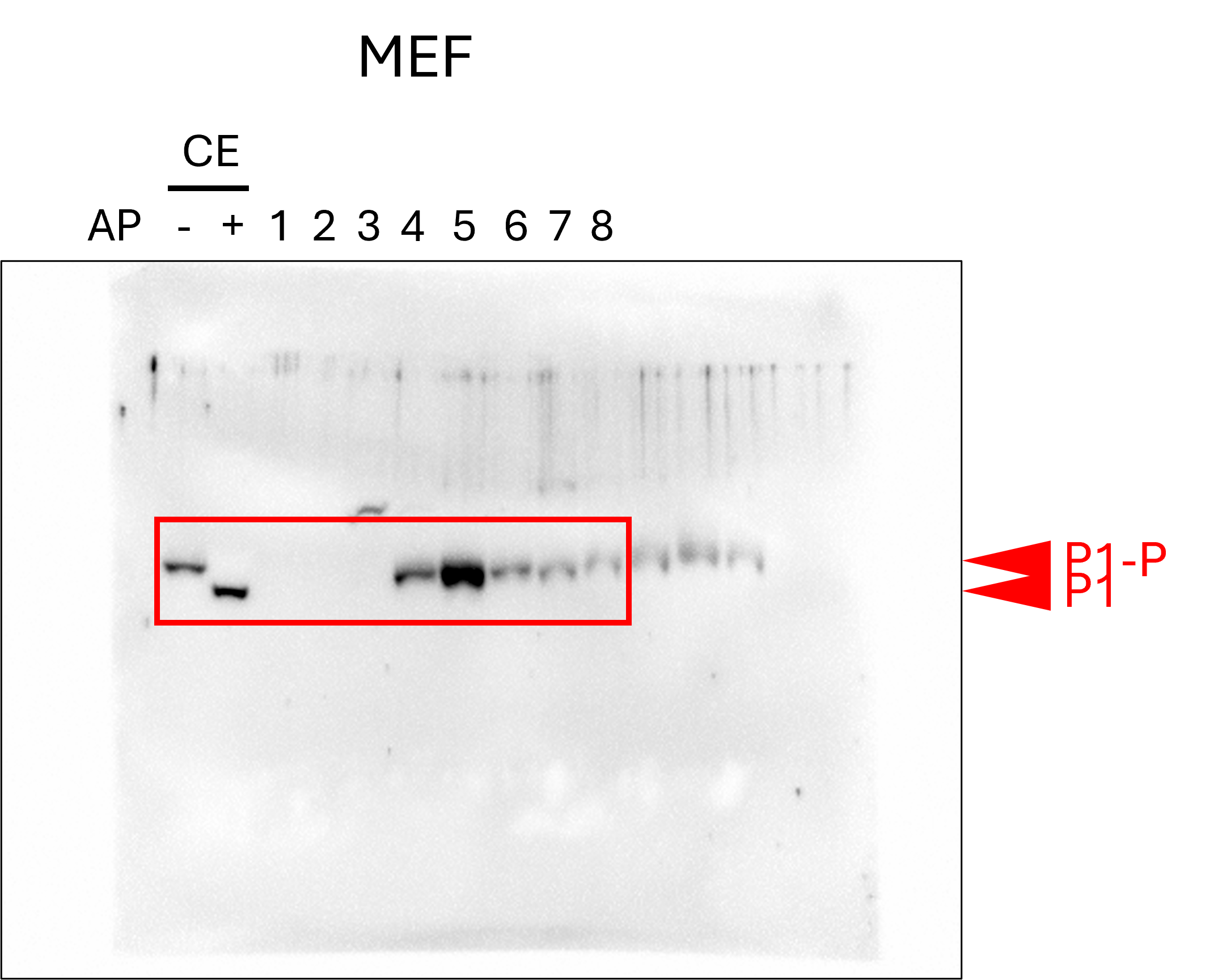

Supplement: Supplementary file 4 — Source data Fig. 3 [file 44319_2024_297_MOESM4_ESM.zip › Figure 3/Fig3B - Western blot MEF P1 polysomes.tif]

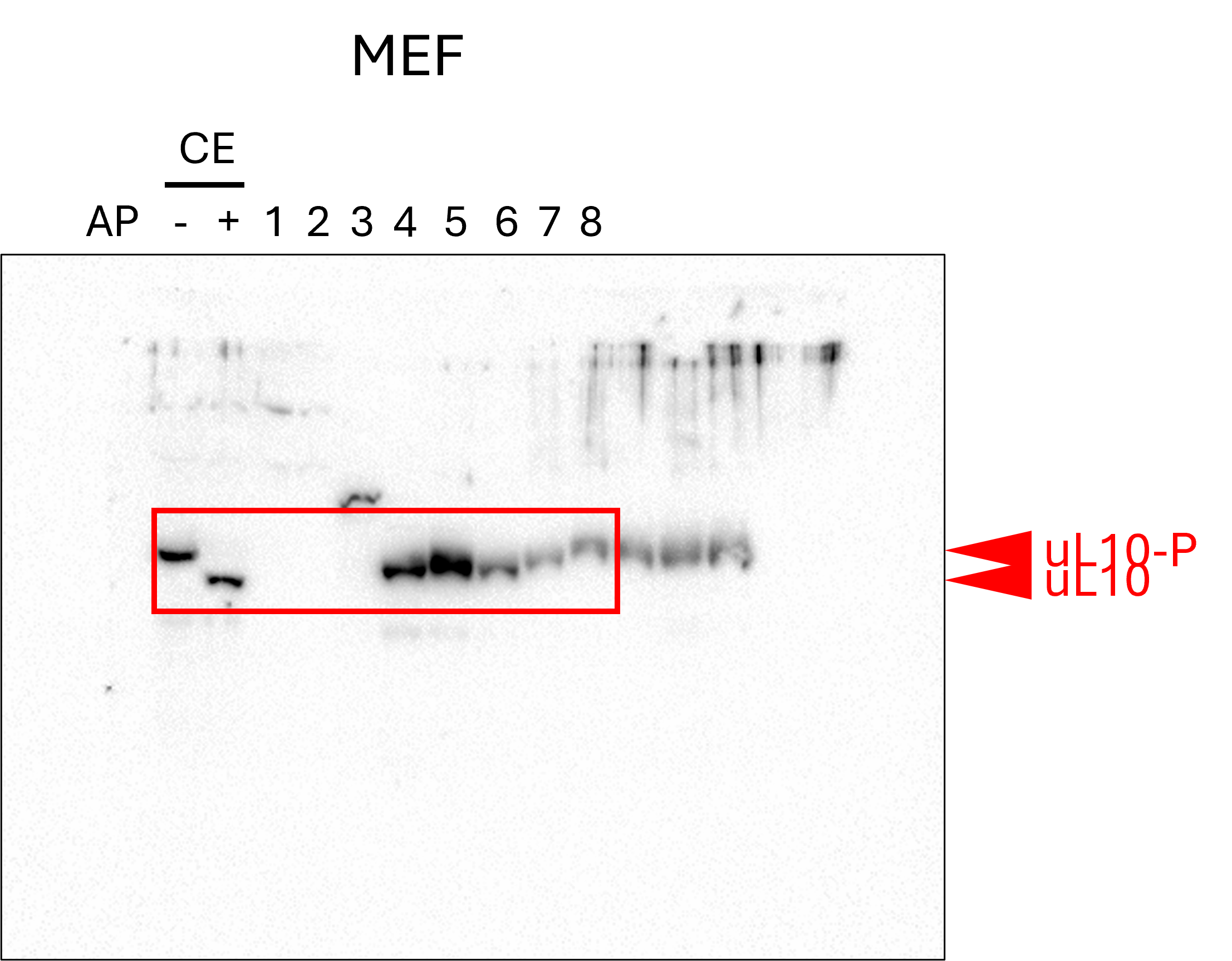

Supplement: Supplementary file 4 — Source data Fig. 3 [file 44319_2024_297_MOESM4_ESM.zip › Figure 3/Fig3B - Western blot MEF uL10 polysomes.tif]

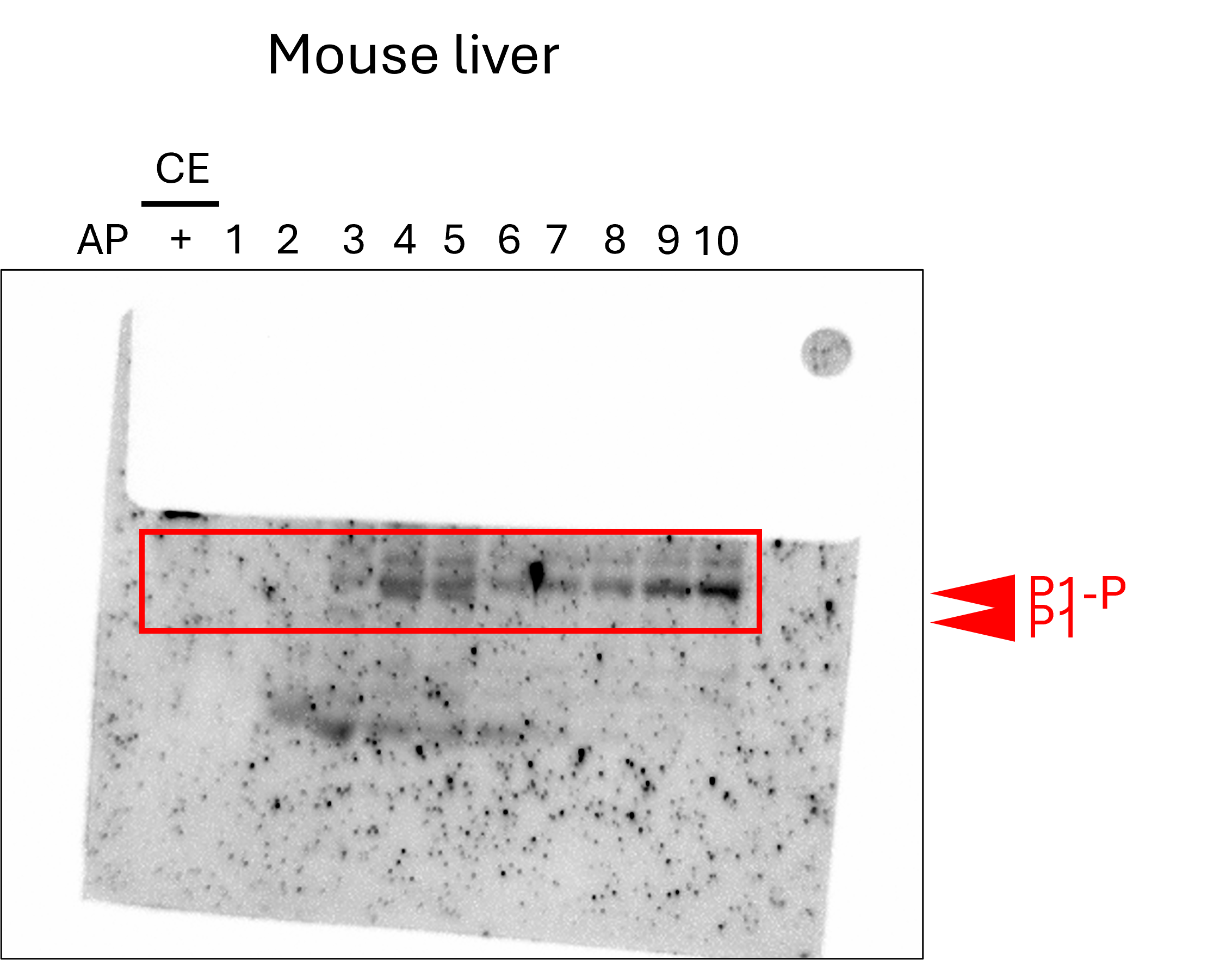

Supplement: Supplementary file 4 — Source data Fig. 3 [file 44319_2024_297_MOESM4_ESM.zip › Figure 3/Fig3B - Western blot mouse liver P1 polysomes.tif]

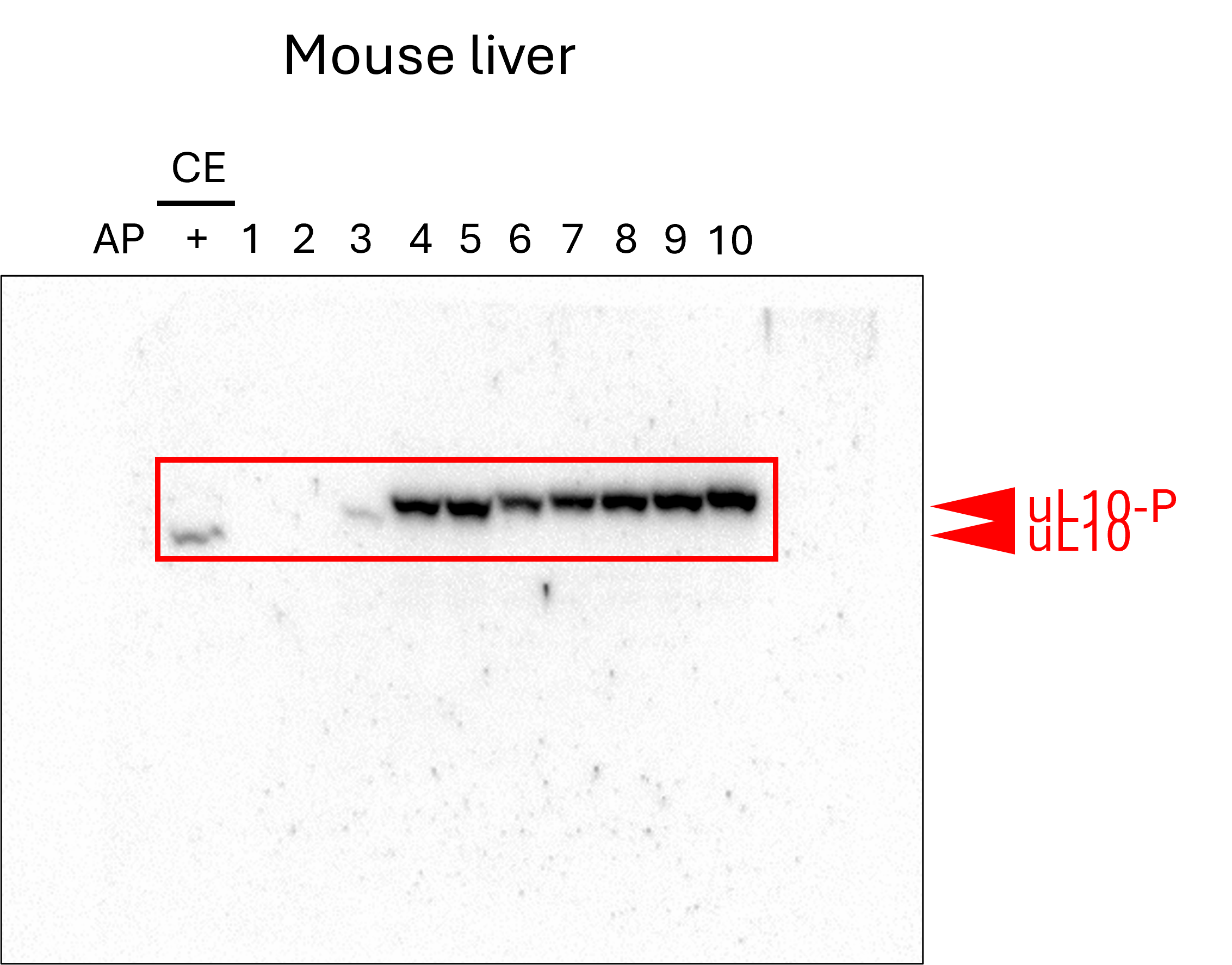

Supplement: Supplementary file 4 — Source data Fig. 3 [file 44319_2024_297_MOESM4_ESM.zip › Figure 3/Fig3B - Western blot mouse liver uL10 polysomes.tif]

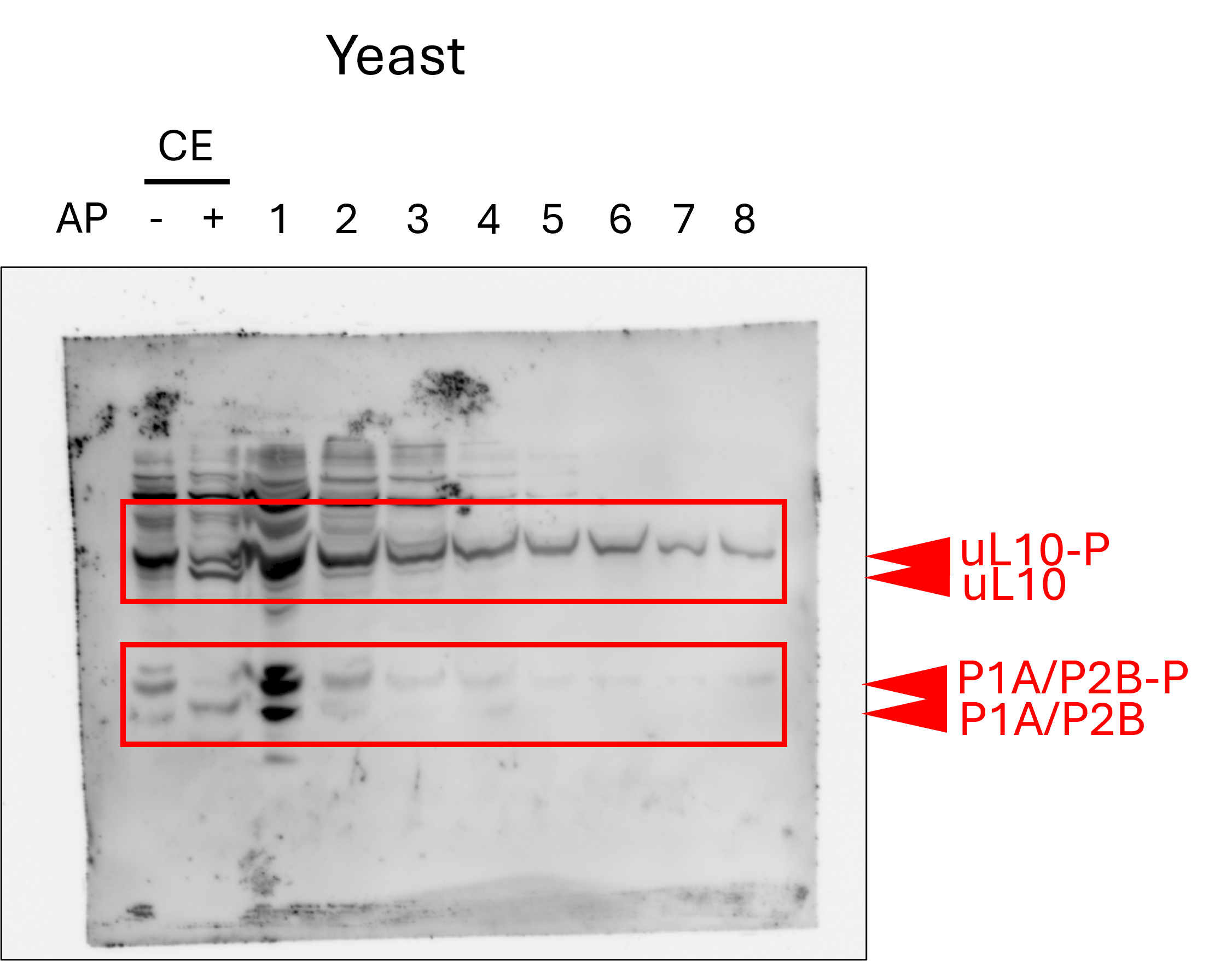

Supplement: Supplementary file 4 — Source data Fig. 3 [file 44319_2024_297_MOESM4_ESM.zip › Figure 3/Fig3B - Western blot yeast uL10 and P1AP2B polysomes.tif]

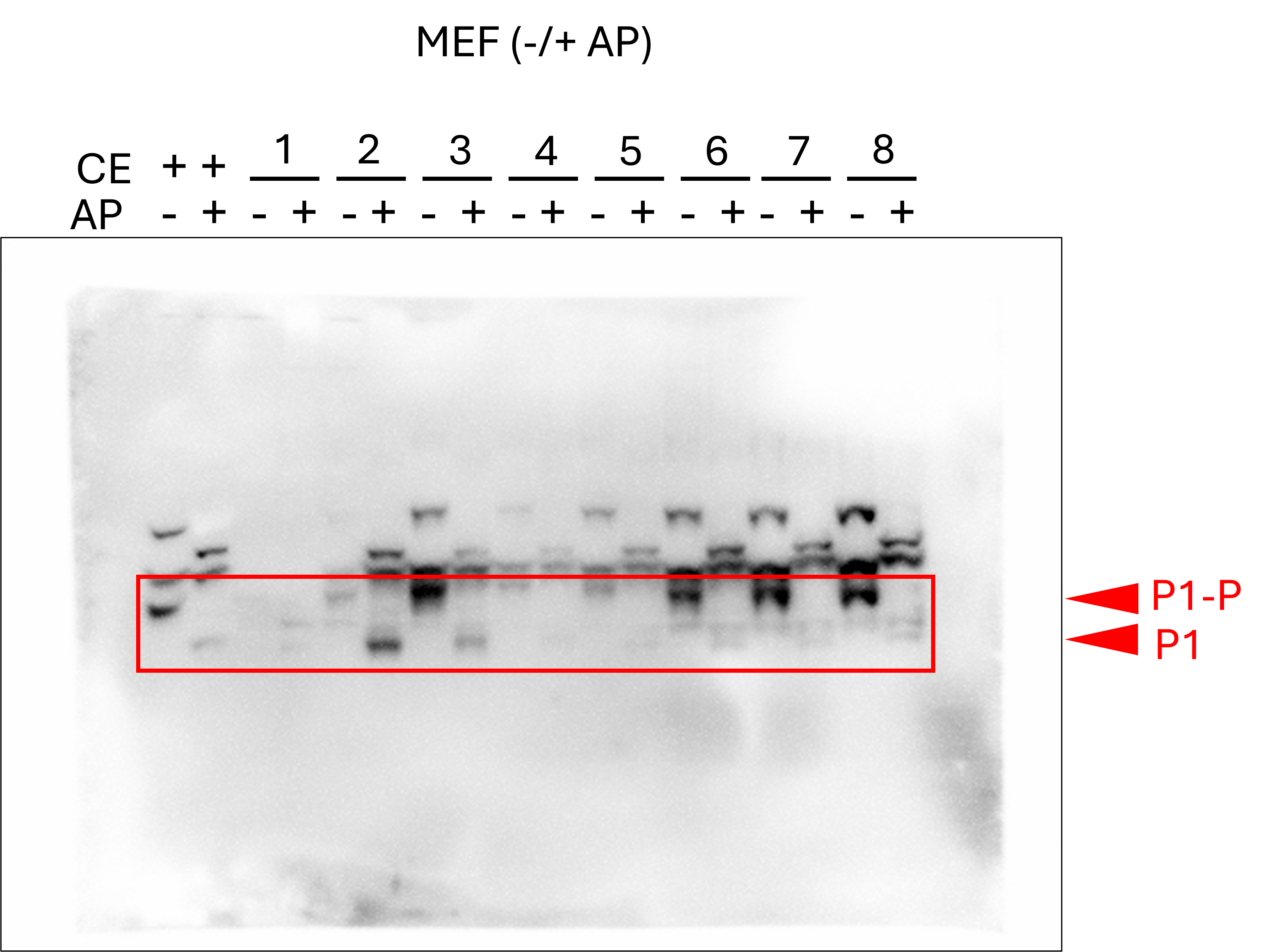

Supplement: Supplementary file 5 — Source data Fig. 4 [file 44319_2024_297_MOESM5_ESM.zip › Figure 4/Fig4B - Western blot MEF P1 polysomes phos-tag.tif]

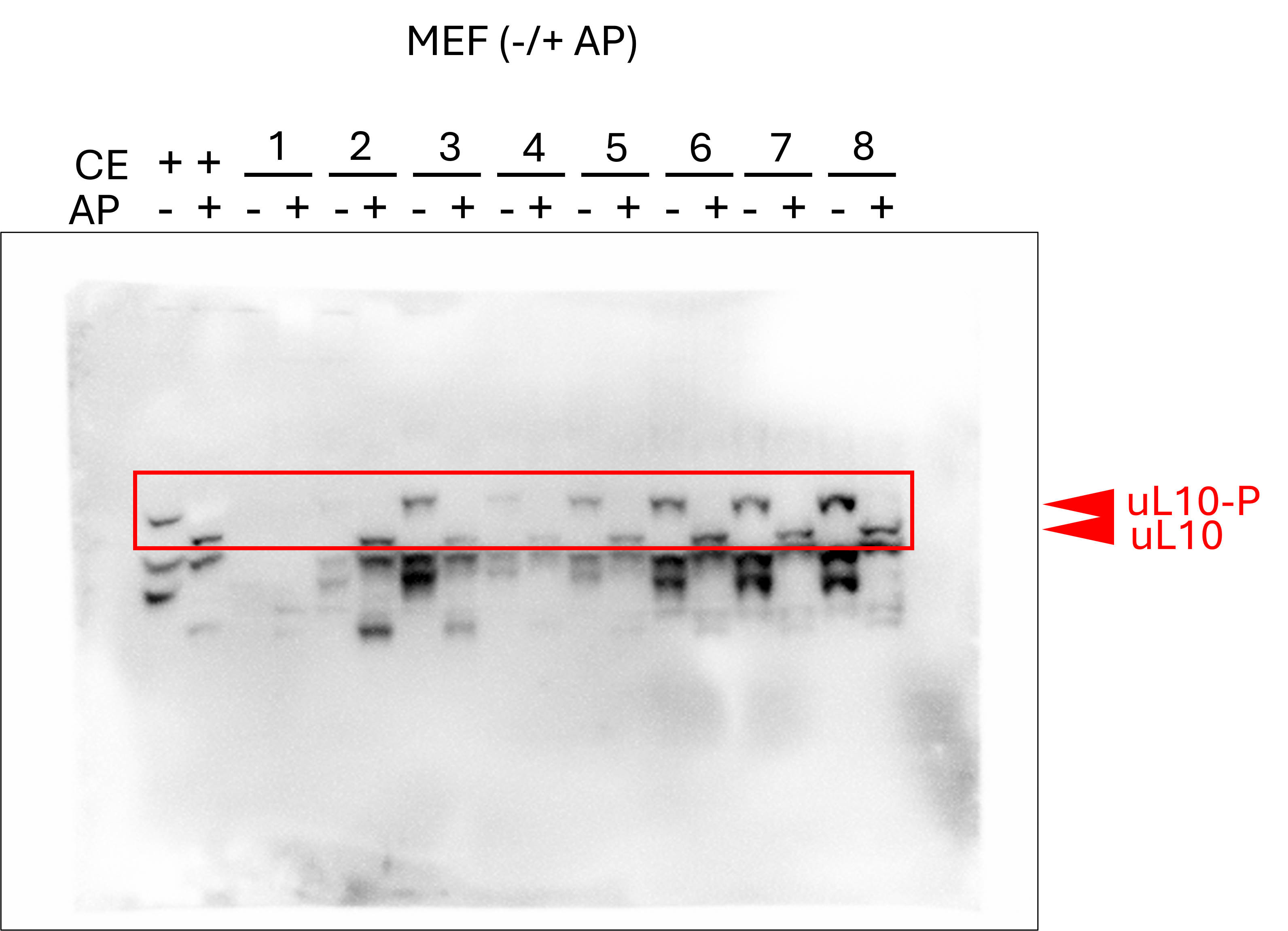

Supplement: Supplementary file 5 — Source data Fig. 4 [file 44319_2024_297_MOESM5_ESM.zip › Figure 4/Fig4B - Western blot MEF uL10 polysomes phos-tag.tif]

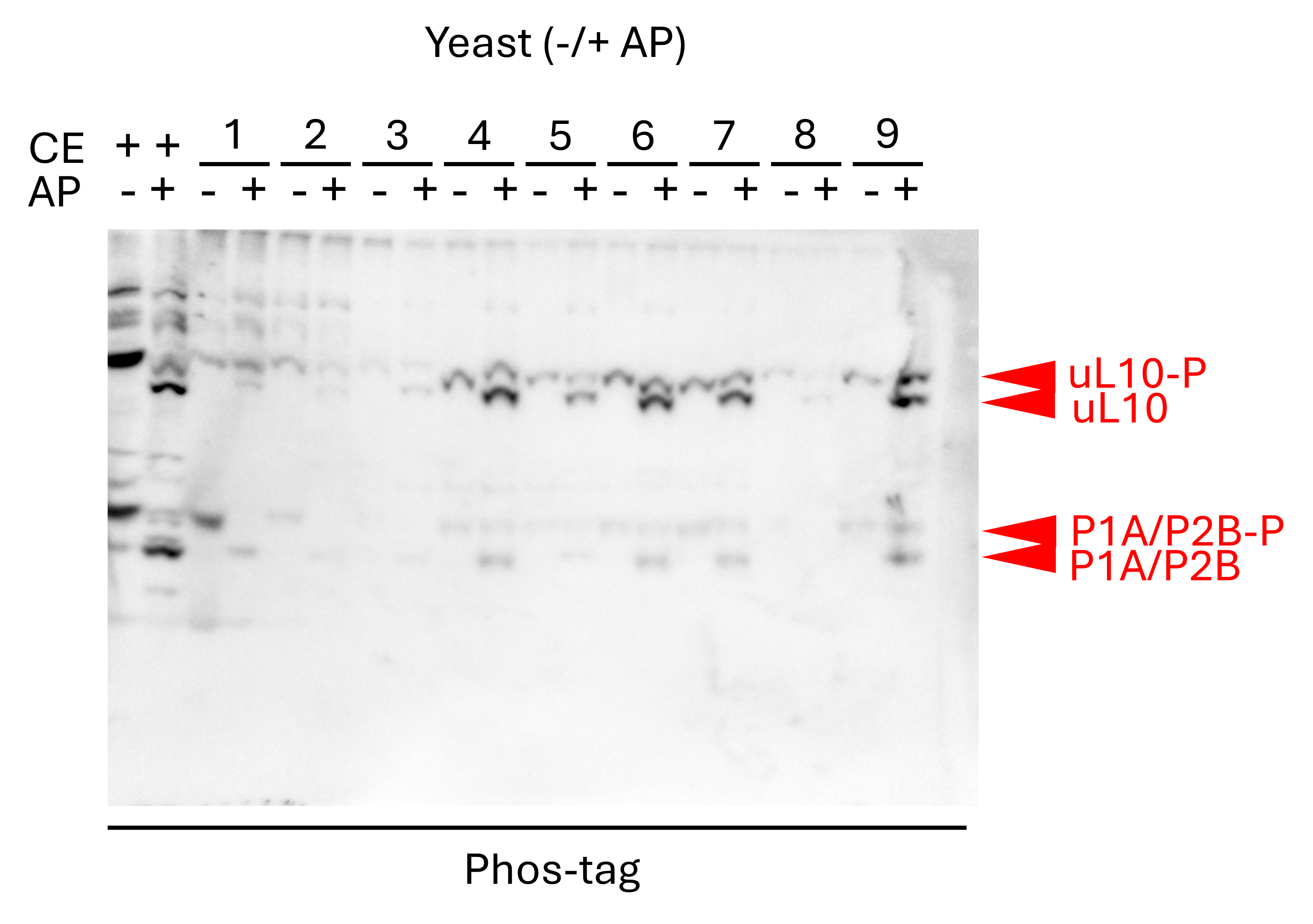

Supplement: Supplementary file 5 — Source data Fig. 4 [file 44319_2024_297_MOESM5_ESM.zip › Figure 4/Fig4B - Western blot yeast polysomes.png]

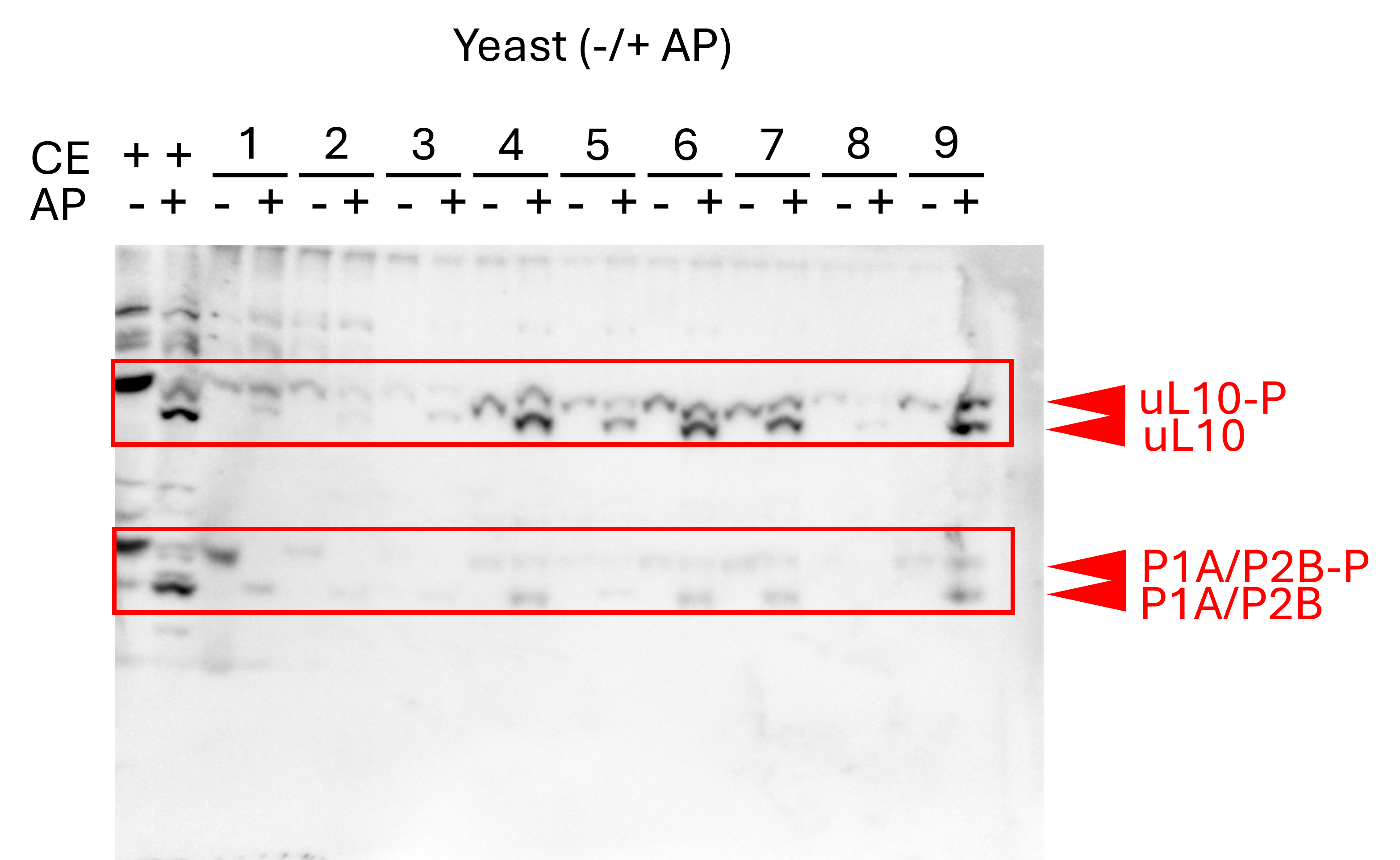

Supplement: Supplementary file 5 — Source data Fig. 4 [file 44319_2024_297_MOESM5_ESM.zip › Figure 4/Fig4B - Western blot yeast uL10 and P1AP2B polysomes phos-tag.tif]

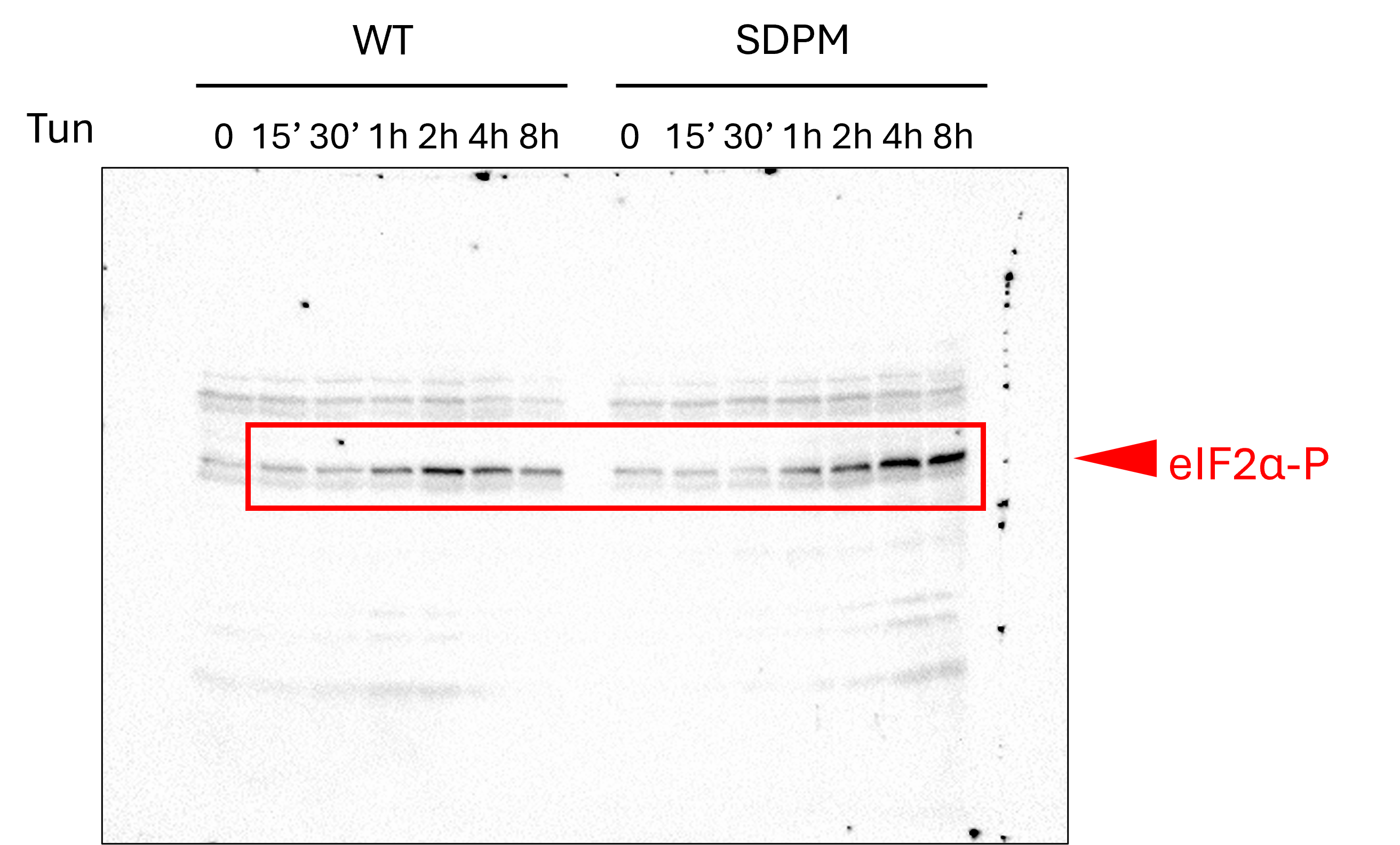

Supplement: Supplementary file 10 — Source data Fig. 9 [file 44319_2024_297_MOESM10_ESM.zip › Figure 9/Fig9A - Western blot yeast eIF2a.tif]

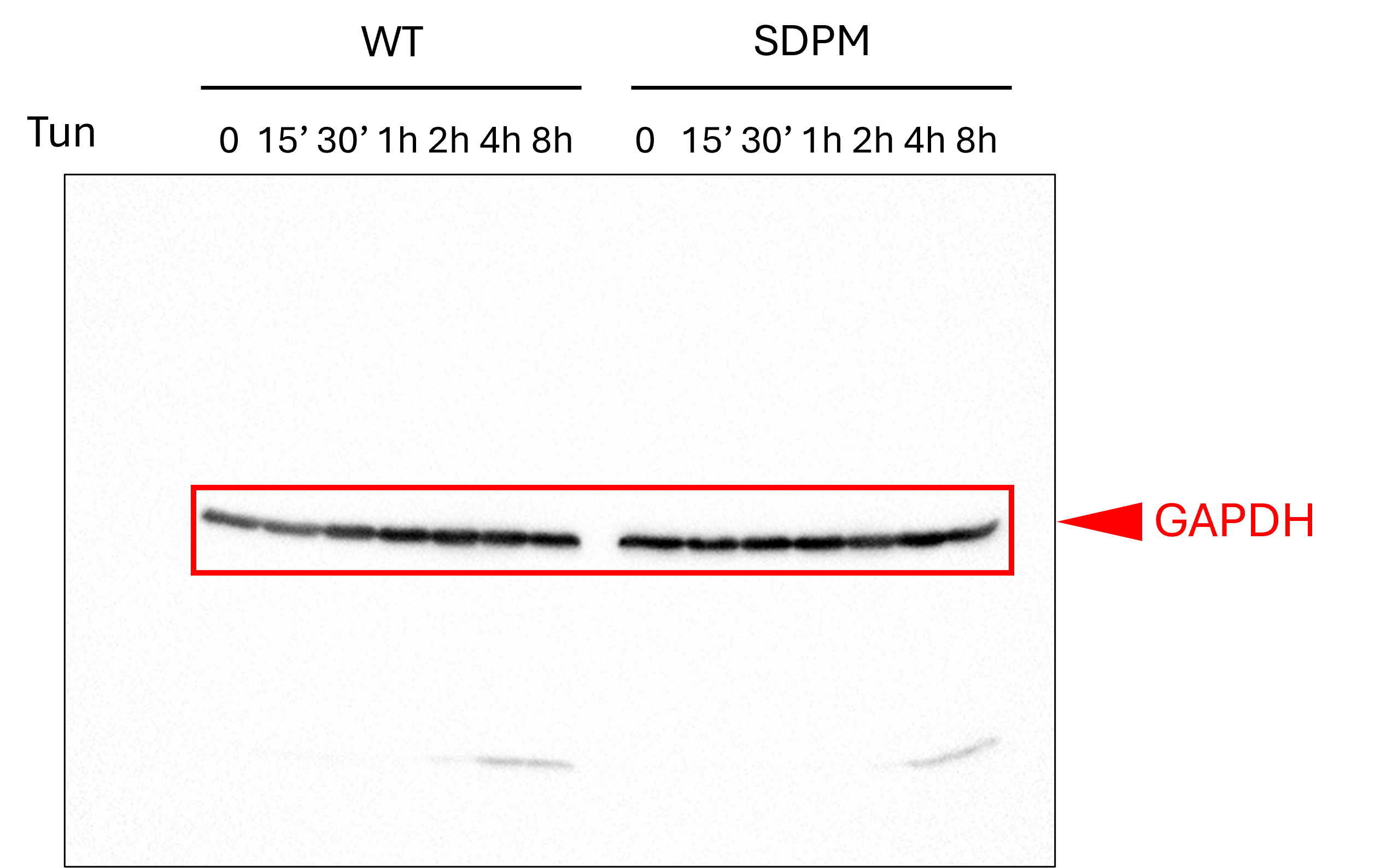

Supplement: Supplementary file 10 — Source data Fig. 9 [file 44319_2024_297_MOESM10_ESM.zip › Figure 9/Fig9A - Western blot yeast GAPDH.tif]

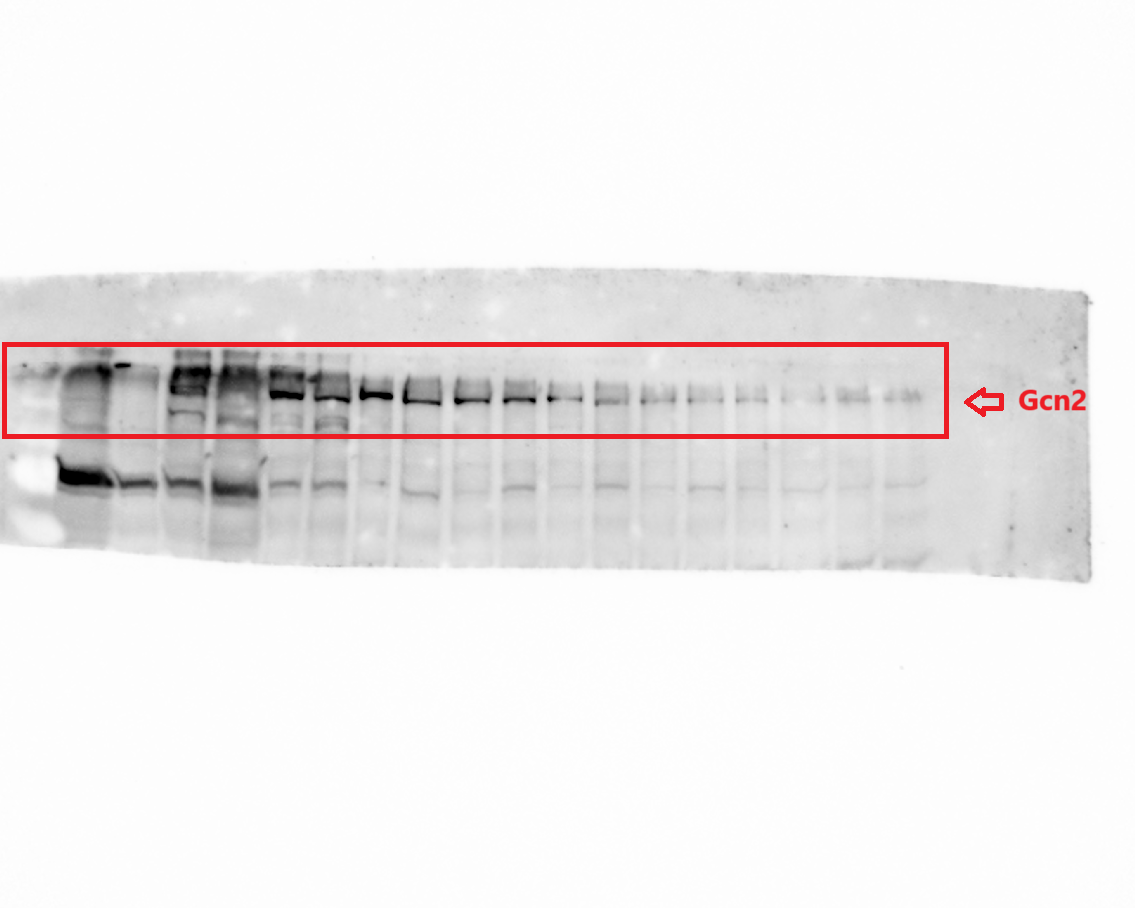

Supplement: Supplementary file 10 — Source data Fig. 9 [file 44319_2024_297_MOESM10_ESM.zip › Figure 9/Fig9B - Western blot SDPM polysomes GCN2.tif]

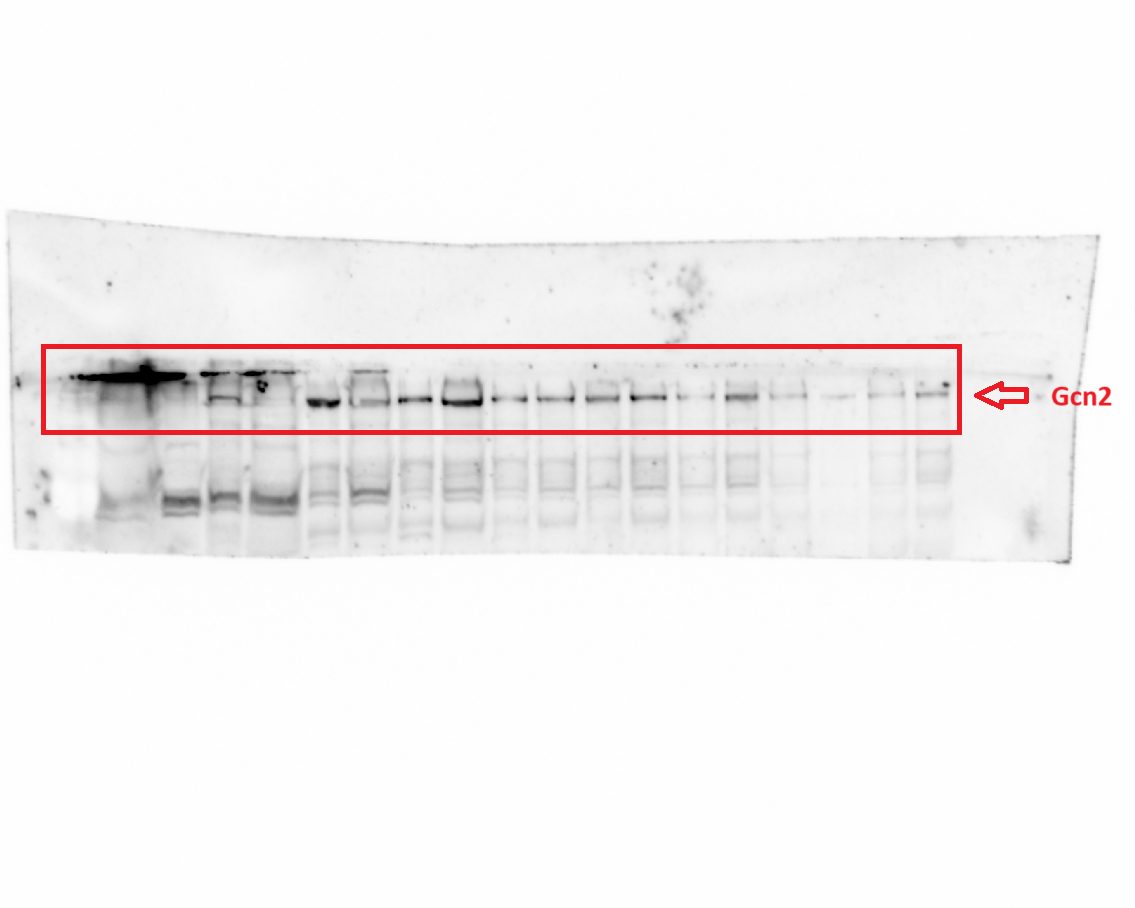

Supplement: Supplementary file 10 — Source data Fig. 9 [file 44319_2024_297_MOESM10_ESM.zip › Figure 9/Fig9B - Western blot WT polysomes GCN2.tif]

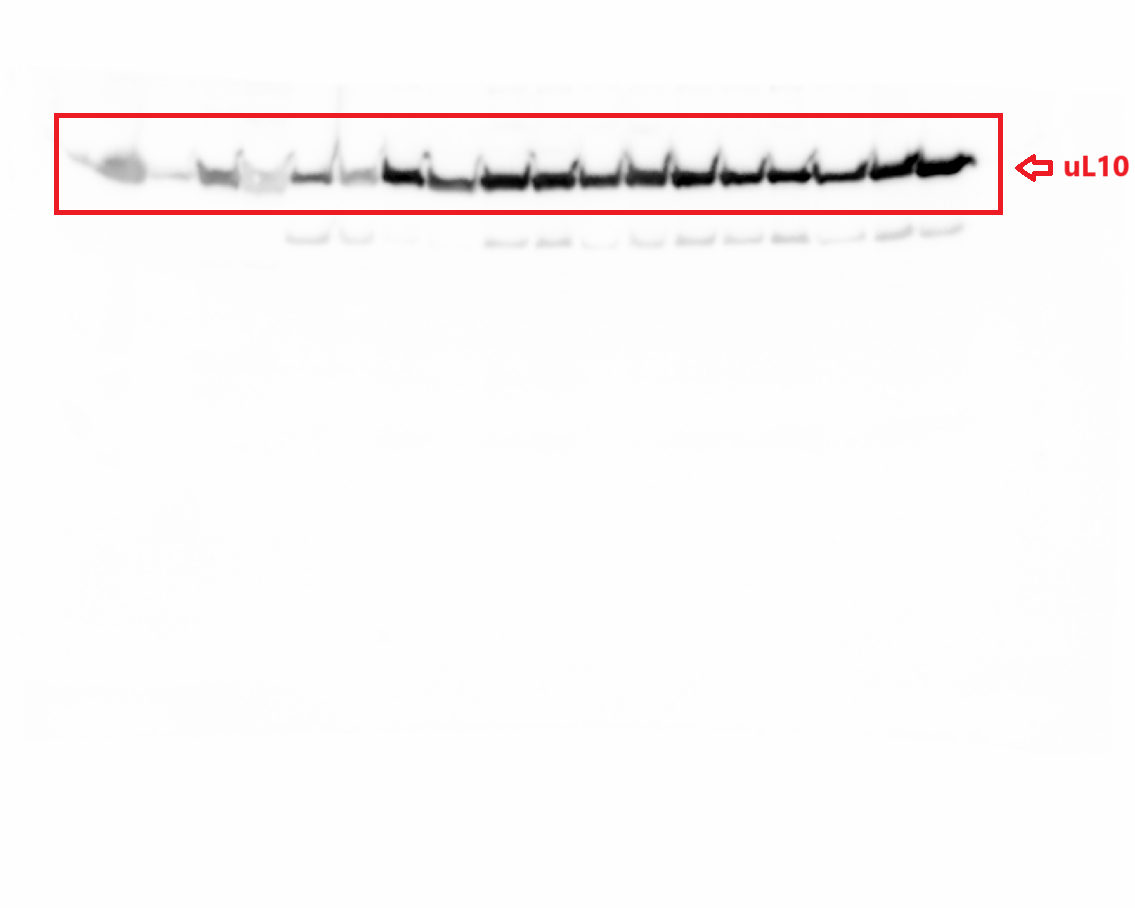

Supplement: Supplementary file 10 — Source data Fig. 9 [file 44319_2024_297_MOESM10_ESM.zip › Figure 9/Fig9B - Western blot SDPM polysomes uL10.tif]

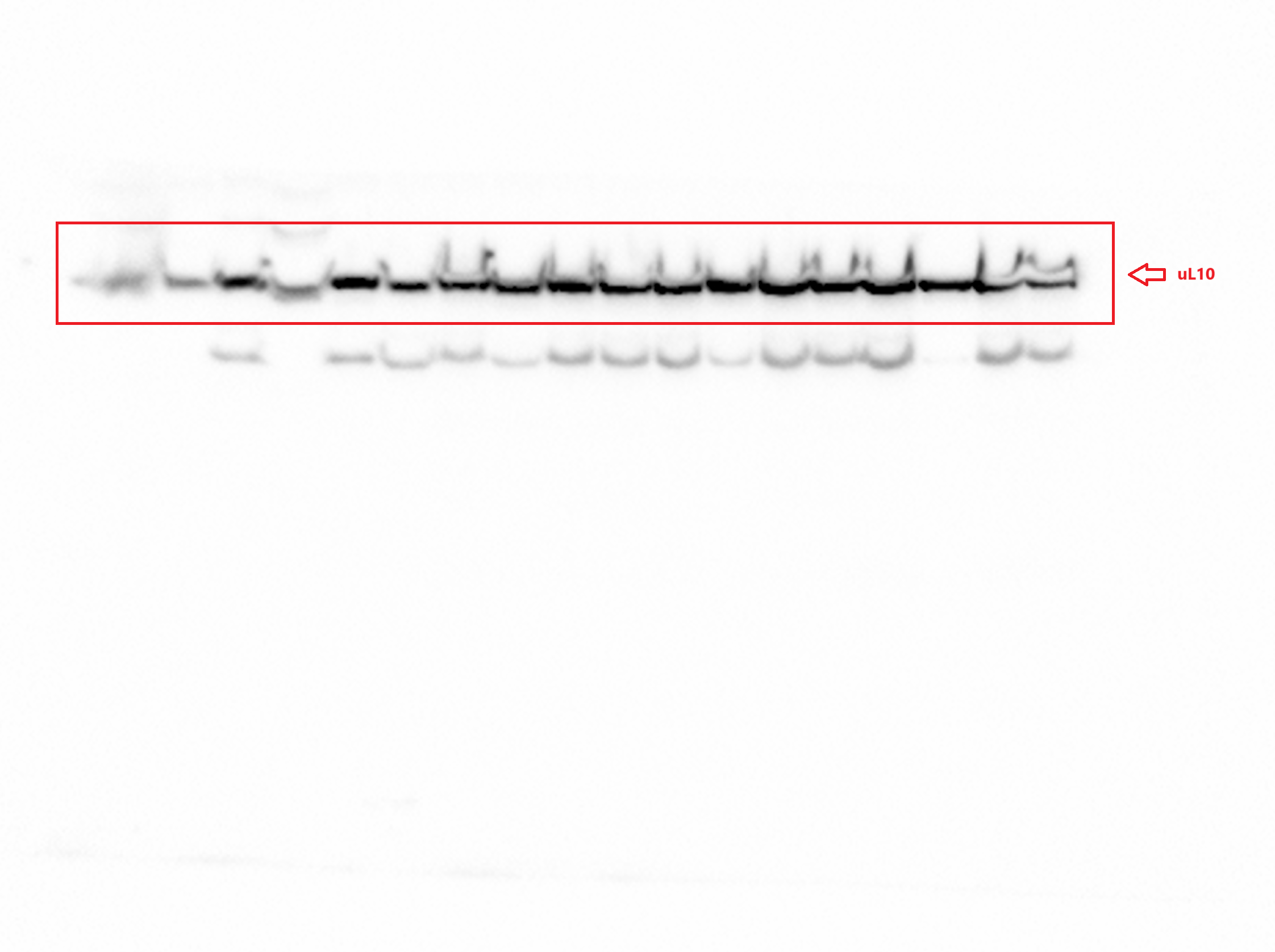

Supplement: Supplementary file 10 — Source data Fig. 9 [file 44319_2024_297_MOESM10_ESM.zip › Figure 9/Fig9B - Western blot WT polysomes uL10.tif]
