# Supplementary material for: Phosphorylation of P-stalk proteins defines the ribosomal state for interaction with auxiliary protein factors
Source: EMBO Rep. 2024 Oct 28;25(12):5478–506. doi: 10.1038/s44319-024-00297-1 (PMC11624264; doi:10.1038/s44319-024-00297-1)
Supplement: Supplementary file 11 — Expanded View Figures [file 44319_2024_297_MOESM11_ESM.pdf]

Expanded View Figures

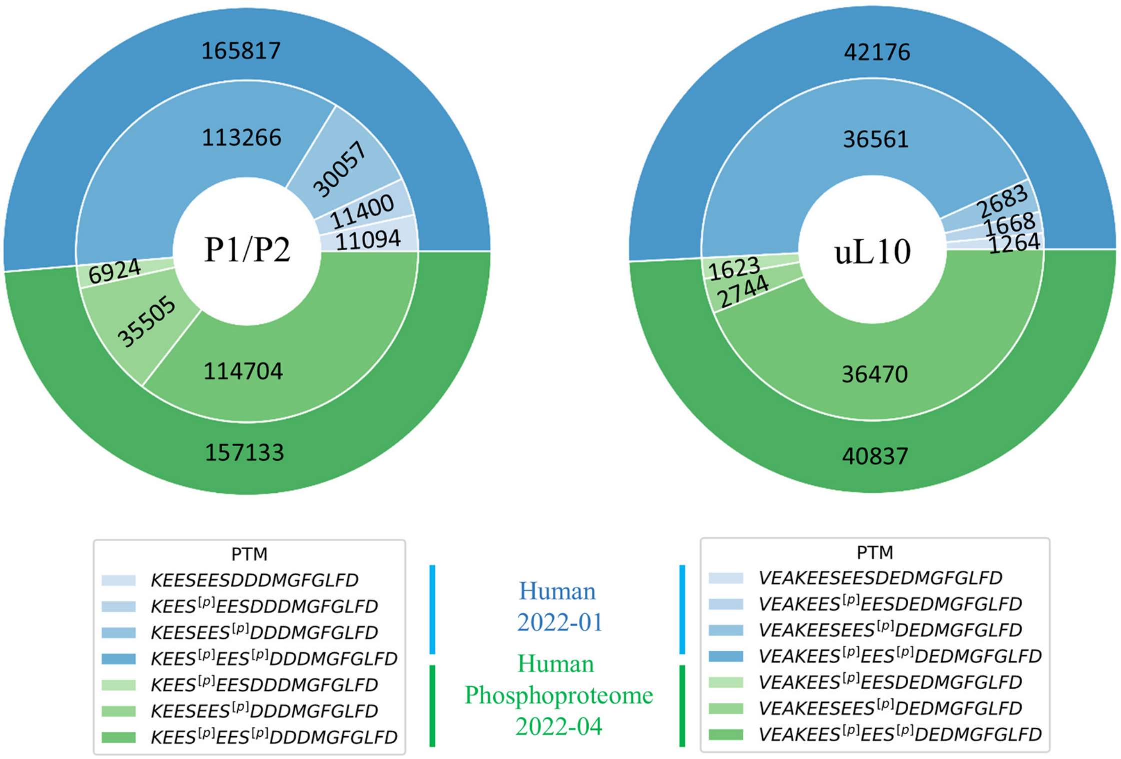

Figure EV1. Meta-analysis of CTD P-stalk protein phosphorylation in mammalian cells using mass spectrometry data implemented in the PeptideAtlas database.

The nested pie charts show the identified peptides of the P1/P2 (left chart) and uL10 (right chart) proteins. The upper, blue part of the plots represents the observation from the entire Human Proteome 2022-01 assembly. The lower, green part of the plots shows the results from the Human Phosphoproteome 2022-04 build. The Human Phosphoproteome 2022-04 contains only phosphorylated P1/P2 and uL10 proteins. The complete Human Proteome 2022-01 includes the observation for non-phosphorylated P-stalk proteins. The total number of peptide observations is shown in the outer ring of the plots. The inner ring shows the number of results with different patterns of serine residue phosphorylation. The higher the number of observations, the darker the color (blue or green). The lower panel shows the identified peptides within the two datasets, 2022-01 and 2022-04 builds, in the corresponding colors; phosphorylation site is marked with [p].

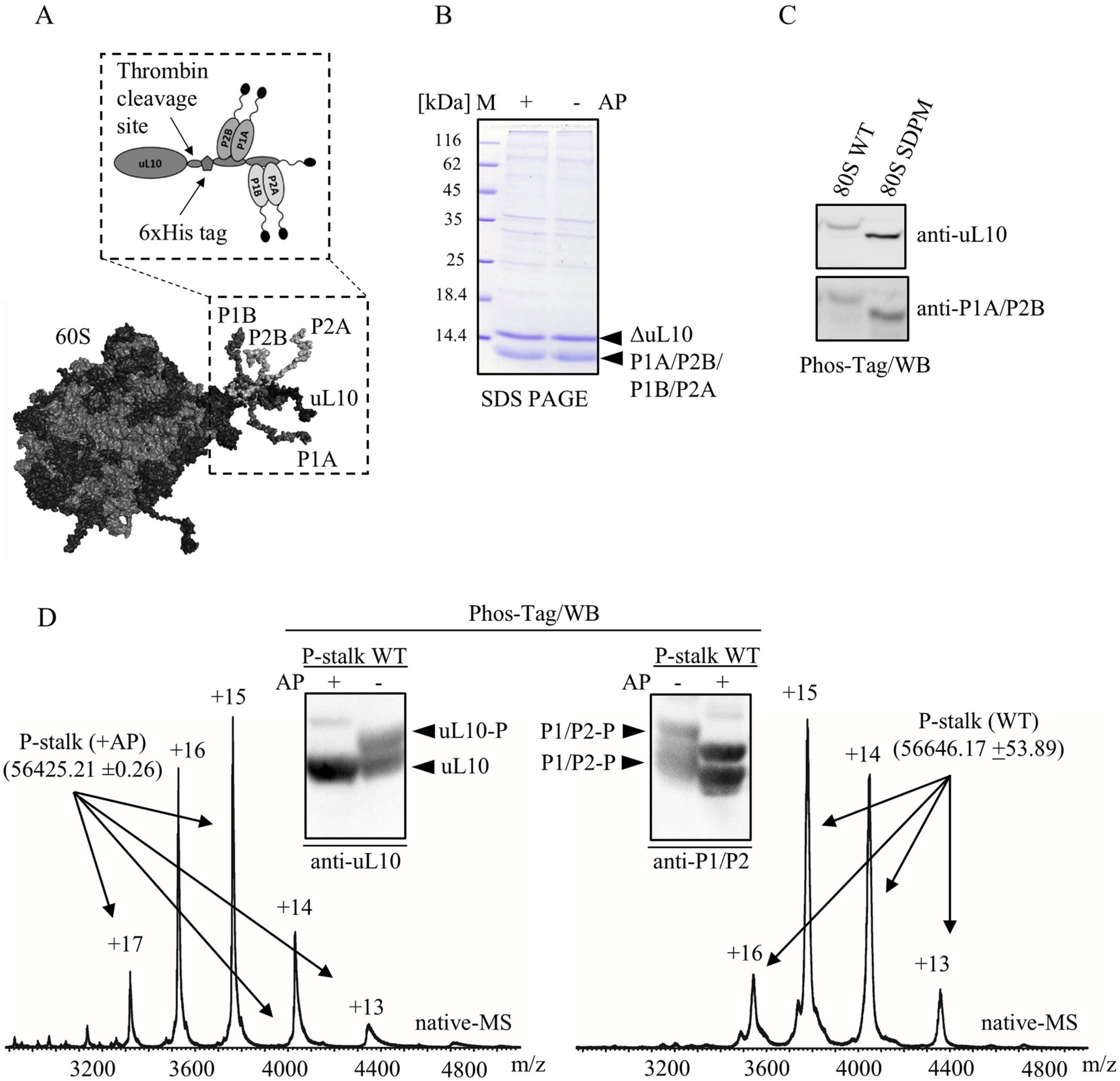

Figure EV2. Purification of phosphorylated (native) and unphosphorylated (AP-treated) P-stalk complexes and 80S ribosomal particles from WT and SDPM mutant strains.

(**A**) Schematic representation of the genetically engineered ribosomal particles with the P-stalk scheme. Specific thrombin cleavage site and 6xHis-tag used for P-stalk release and subsequent purification are indicated with arrows. (**B**) SDS-PAGE analysis of purified native (-AP) and dephosphorylated (+AP) stalk complexes. The positions of ΔuL10 and P1A/P2B/P1B/P2A are pointed with arrows. (**C**) Phos-tag analysis of purified 80S ribosomal particles; 80S WT - ribosomes from WT strain, 80S SDPM - ribsomes from SDPM mutant strain harboring S to A mutations within all P-protein; anti-uL10 and anti-P1A/P2B antibodies were used. (**D**) Native mass spectroscopy analysis (native-MS) of purified intact P-stalk complexes; left and right panels, MS spectra of the complexes of WT and +AP, respectively; numbers next to the peaks indicate the charge states of the complexes; molecular masses of the P-stalk complexes were calculated by MaxEnt deconvolution software (Waters), and are 56646 and 56425 Da for WT and +AP complexes, respectively; insert - phos-tag analysis of purified stalk complexes, WT and +AP. The complexes were analyzed by SDS-PAGE/phos-tag/WB and subsequently proteins were detected with specific antibodies against uL10 and P1/P2 proteins. The positions of phospho- and dephospho-forms are indicated with arrows.

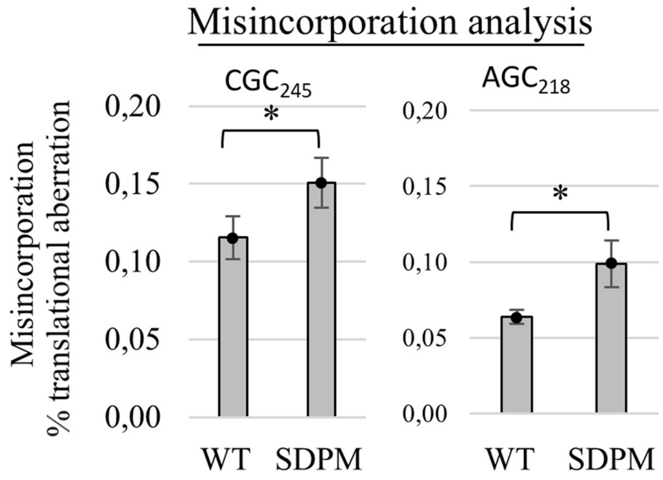

Figure EV3. Misincorporation analysis using a dual-luciferase reporter assay.

CGC_{245} and AGC_{218} describe near-cognate codons at positions 245 and 218 of the firefly reporter enzyme. All data are presented as the percentage of translational aberration; error bars, standard deviations ($n = 3$, technical replicates); $^*p < 0.05$ by Student's t-test is indicated by asterisks.

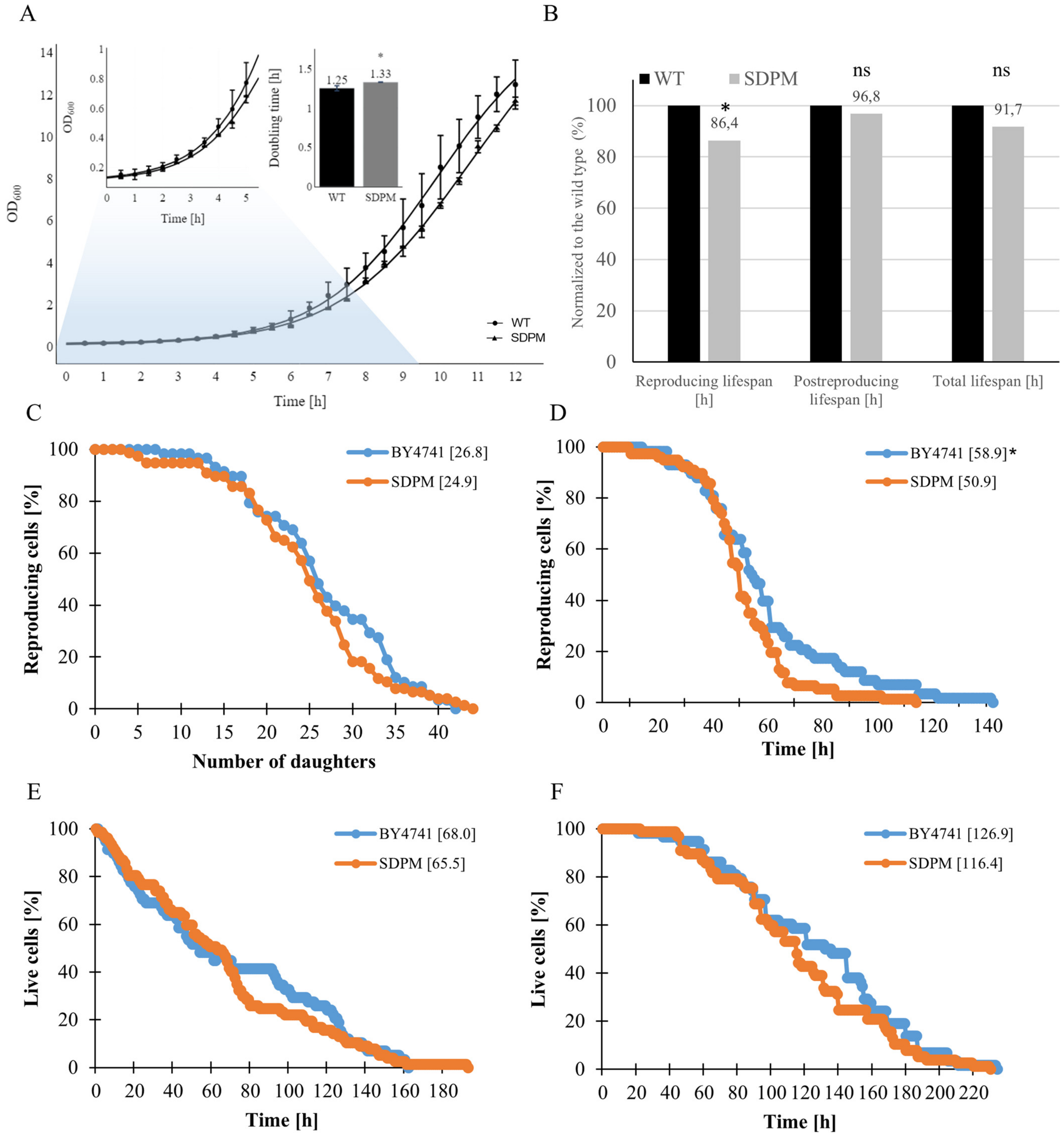

Figure EV4. Cellular fitness of the WT and SDPM yeast strains.

(**A**) Growth curves for WT and SDPM strains. The exponential fragment of growth curves used for doubling time calculation and average doubling time for WT and SDPM are shown in insets. The statistical analysis was done using one-tailed t-Welch test (*$p < 0.05$), data are presented as mean ± SEM of $n = 3$, technical replicates. (**B**) Yeast lifespan analysis of WT and SDPM strains on the single cell level; the values were normalized to the WT referred as 100%, using data presented in (**D–F**). To assess differences between the WT and SDPM strains, one-way ANOVA and Dunnett's post hoc tests were used (*$p < 0.05$), ns not significant. Comparison of the reproductive potential (**A**), reproductive lifespan (**B**), post-reproductive lifespan (**C**), and total lifespan (**D**) of the haploid reference yeast strain BY4741 (wild-type—WT) and the mutant SDPM strain. Statistical significances were assessed using ANOVA and Dunnett's post hoc test (*$p < 0.05$). The mean value for a total of 90 cells from two independent experiments is shown in parentheses.

 © The Author(s)

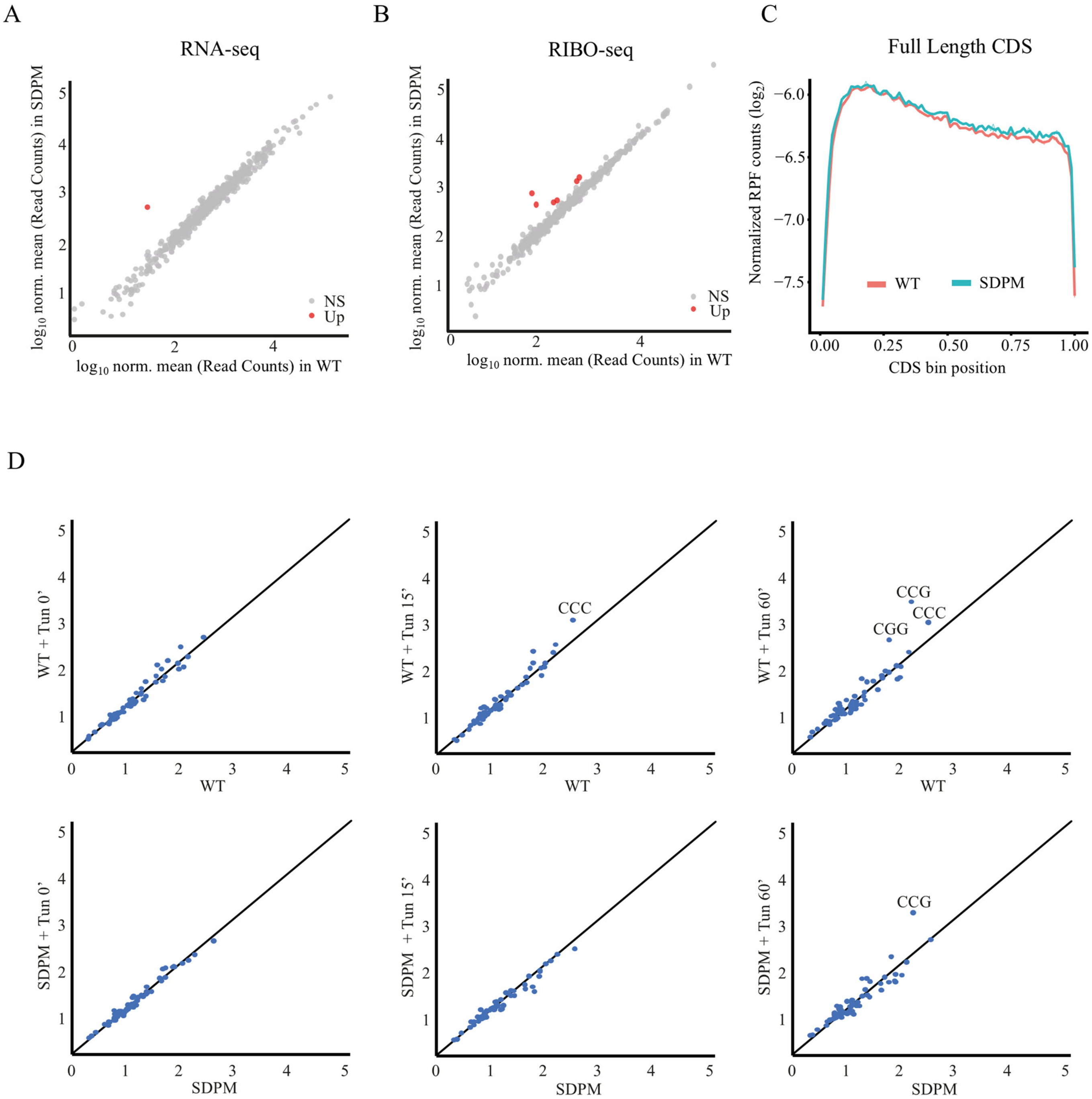

Figure EV5. The ribosomal footprints distribution obtained based on a genome-wide scale analysis of WT and SDPM yeast strains.

(**A**, **B**) Cross-correlation of gene expression analyses at the level of transcription (RNA-seq - **A**) and translation (RIBO-seq - **B**) for WT vs SDPM mutant strains; (**C**) average ribosome occupancy from all genes aligned from start to stop codons within the coding sequence (CDS) for WT (red line) and SDPM mutant strain (blue line); ribosome occupancy was normalized to show a mean value of 1 for each codon; the footprint occupancy was shown for the whole CDS. (**D**) The correlation of specific codon occupancies within 28 nt RPF. Specific overrepresented codons, CGG for arginine and CCG, CCC for proline are indicated.
